# Do prenatal factors shape the risk for dementia?: A systematic review of the epidemiological evidence for the prenatal origins of dementia

**DOI:** 10.1007/s00127-023-02471-7

**Published:** 2023-04-08

**Authors:** Aline Marileen Wiegersma, Amber Boots, Miranda W. Langendam, Jacqueline Limpens, Susan D. Shenkin, Aniko Korosi, Tessa J. Roseboom, Susanne R. de Rooij

**Affiliations:** 1 Epidemiology and Data Science, Amsterdam UMC Location University of Amsterdam, Meibergdreef 9, Amsterdam, The Netherlands; 2 Medical Library, Amsterdam UMC Location University of Amsterdam, Meibergdreef 9, Amsterdam, The Netherlands; 3 Geriatric Medicine, Usher Institute, University of Edinburgh, Edinburgh, Scotland UK; 4 Swammerdam Institute for Life Sciences, University of Amsterdam, Amsterdam, The Netherlands; 5 Obstetrics and Gynaecology, Amsterdam UMC Location University of Amsterdam, Meibergdreef 9, Amsterdam, The Netherlands; 6 Amsterdam Public Health Research Institute, Aging & Later Life, Health Behaviors & Chronic Diseases, Amsterdam, The Netherlands; 7 Amsterdam Public Health Research Institute, Methodology, Amsterdam, The Netherlands; 8 Amsterdam Reproduction and Development, Amsterdam, The Netherlands

**Keywords:** Alzheimer’s disease, Dementia, Developmental programming, Prenatal, Systematic review

## Abstract

**Purpose:**

Prenatal factors such as maternal stress, infection and nutrition affect fetal brain development and may also influence later risk for dementia. The purpose of this systematic review was to provide an overview of all studies which investigated the association between prenatal factors and later risk for dementia.

**Methods:**

We systematically searched MEDLINE and Embase for original human studies reporting on associations between prenatal factors and dementia from inception to 23 November 2022. Prenatal factors could be any factor assessed during pregnancy, at birth or postnatally, provided they were indicative of a prenatal exposure. Risk of bias was assessed using the Newcastle Ottawa Scale. We followed PRISMA guidelines for reporting.

**Results:**

A total of 68 studies met eligibility criteria (including millions of individuals), assessing maternal age (N = 30), paternal age (N = 22), birth order (N = 15), season of birth (N = 16), place of birth (N = 13), prenatal influenza pandemic (N = 1) or Chinese famine exposure (N = 1), birth characteristics (N = 3) and prenatal hormone exposure (N = 4). We observed consistent results for birth in a generally less optimal environment (e.g. high infant mortality area) being associated with higher dementia risk. Lower and higher birth weight and prenatal famine exposure were associated with higher dementia risk. The studies on season of birth, digit ratio, prenatal influenza pandemic exposure, parental age and birth order showed inconsistent results and were hampered by relatively high risk of bias.

**Conclusion:**

Our findings suggest that some prenatal factors, especially those related to a suboptimal prenatal environment, are associated with an increased dementia risk. As these associations may be confounded by factors such as parental socioeconomic status, more research is needed to examine the potential causal role of the prenatal environment in dementia.

**Supplementary Information:**

The online version contains supplementary material available at 10.1007/s00127-023-02471-7.

## Introduction

The number of people living with dementia worldwide is rising [1]. In addition to genetic factors, environmental and lifestyle factors like smoking, sedentary behavior and alcohol consumption are known risk factors for dementia [1–3]. Given that the majority of brain development occurs prenatally, and that the brain is highly vulnerable during the prenatal period, factors occurring during the earliest stages of life may also affect the development of dementia.

Many chronic disorders, including hypertension, type 2 diabetes mellitus (T2DM) and cardiovascular diseases (CVD) have known prenatal origins [4]. For instance, small size at birth, which is a marker for restricted fetal growth, has frequently been associated with a higher risk for hypertension, T2DM, CVD and CVD related mortality [4–6]. Also, exposure to undernutrition during fetal development has been associated with increased risks of cardiovascular and metabolic diseases in late adulthood [7–10]. These associations are thought to result from lasting adaptations to the fetal environment that permanently affected physiology and metabolism [11]. In addition to human studies, animal studies have provided a large body of evidence for the developmental origins of health and disease and mechanisms underlying these associations [12–14]. Adverse prenatal factors have been shown to induce cardiovascular and metabolic symptoms in many animal models, including hypertension, hyperglycemia and insulin resistance [14]. A potential role for prenatal factors in dementia has received relatively little attention. Studies have shown that prenatal factors can have lasting consequences for future brain size, structure and function [7, 15]. In studies in humans, undernutrition during fetal development has been associated with smaller brain size, increased BrainAGE (an MRI-based measure providing an estimate for the clinical aging of the brain) and worse cognitive function compared to individuals who were unexposed to undernutrition [16–23]. Furthermore, higher birth weight has been positively associated with larger brain size and better cognitive function in older age [24–26]. Smaller brain size, increased BrainAGE and worse cognitive function all relate to an increased risk for dementia and these findings thus suggest a role for prenatal factors in dementia [15, 27, 28]. In addition, animal studies have shown that prenatal and early postnatal factors can have a profound impact on aging, later cognitive function and the development of Alzheimer’s disease-specific neuropathological features [15, 29].

Two previous reviews have discussed early-life risk factors for Alzheimer’s disease (AD) in humans, one of which was conducted systematically [27, 28]. These studies mostly included postnatal factors and only included AD as an outcome, thereby missing studies regarding other types of dementia (e.g. vascular dementia). Therefore, it remains unclear if and how prenatal factors affect the risk for dementia in humans. The aim of this systematic review was to provide an overview of all studies which investigated the association between prenatal factors and clinical or autopsy proven diagnosis of dementia in humans.

## Methods

The protocol for the present study was registered at the international Prospective Register of Systematic Reviews (PROSPERO) (CRD42020165725). We slightly deviated from our initially registered protocol, a revised version has been uploaded. We followed PRISMA guidelines for reporting (see checklist in Online Resource 1).

### Eligibility criteria

We included human cohort, case–control and cross-sectional studies that addressed the association between any prenatal factor and a clinical and/or autopsy proven diagnosis of dementia or dementia registered as a cause of death. Although we did not expect any randomized controlled trials regarding this topic, they would theoretically also be eligible for inclusion in the current study. Dementia could be of any type or unspecified (e.g. Alzheimer’s disease, vascular dementia, lewy body dementia, frontotemporal dementia). Studies were eligible if they investigated specific prenatal exposures, prenatal socioeconomic factors, relevant parental factors (also prior to conception), pregnancy characteristics, birth outcomes (e.g. birth weight) and factors indicative of a specific prenatal exposure (e.g. digit ratio for prenatal sex hormone exposure). As we aimed to provide an overview of all prenatal factors studied in relation to dementia we did not pre-specify all factors that would adhere to our inclusion criteria.

We excluded studies investigating a population of individuals affected by genetic syndromes associated with dementia (e.g. Down’s syndrome), multiple sclerosis or dementias with a known established origin (e.g. Korsakoff’s syndrome or familial early-onset Alzheimer’s disease) as prenatal factors are not likely to play a major role in their development.

### Search strategy

An information specialist (JL) searched OVID MEDLINE and Embase from inception to November 23, 2022 (see the full search strategy in Online Resource 2). The search strategy comprised Subject Headings (i.e. MeSH-terms) and text words for 1. dementia and 2. prenatal exposure, general and specific (known) prenatal and birth related factors. We used methodological search filters to limit our search to published human trials and observational studies. No further restrictions (i.e. date or language) were applied. We cross-checked the reference list and the citing articles of the identified relevant papers in Web of Science and adapted the search in case of additional relevant papers. The retrieved bibliographic records were imported and de-duplicated in EndNote.

### Study selection and data extraction

Two review authors (AMW and AB) independently screened titles and abstracts of identified studies using Rayyan software [30]. Full texts were obtained and screened for all articles possibly meeting the inclusion criteria. Data extraction was performed independently by the same authors using a standardized form. Disagreements in study selection and data extraction were discussed and solved in the presence of a third reviewer (SdR). Extracted data items included the aim, study design, location, number of participants, publication year, the relevant exposure(s) studied, the relevant study outcomes, outcome assessment method (and diagnostic criteria used), mean age of participants at outcome assessment, possible confounders and mediators reported, statistical relationship and direction and size of the reported association(s) including subgroup results.

### Risk of bias

Risk of bias was assessed by two review authors independently (AMW and AB) with the Newcastle Ottawa Scale (NOS) for assessing the quality of observational studies [31]. We used an adapted version of the NOS to assess risk of bias for cross-sectional studies [32]. Disagreements were solved with a discussion, if necessary in the presence of a third reviewer (SdR). Regarding comparability of study samples, one star was awarded for controlling for age or year of birth in the design or analysis and one star was awarded if at least one other additional covariate was accounted for. As the NOS for cohort studies is more focused on prospective cohort studies, we used the NOS for cross-sectional studies for included retrospective cohort studies. For the purpose of summarizing the results, we adhered to a cut-off score of ≥ 7 points (or ≥ 6 for the cross-sectional NOS) to define low risk of bias, otherwise, studies were considered as having intermediate/high risk of bias. The risk of bias scores were taken into account in the discussion and conclusion of our review as we treated results of studies with intermediate or high risk of bias as being less certain.

### Data synthesis

Studies were categorized on the basis of the prenatal factor and outcomes. Studies with similar exposures and outcomes were assessed for homogeneity to be included in a meta-analysis. A meta-analysis was performed if there were at least three studies regarding a specific exposure and dementia sub-type, the necessary data was available (i.e. numerical and statistical results) and we considered these studies sufficiently comparable.

## Results

**Fig. 1:**
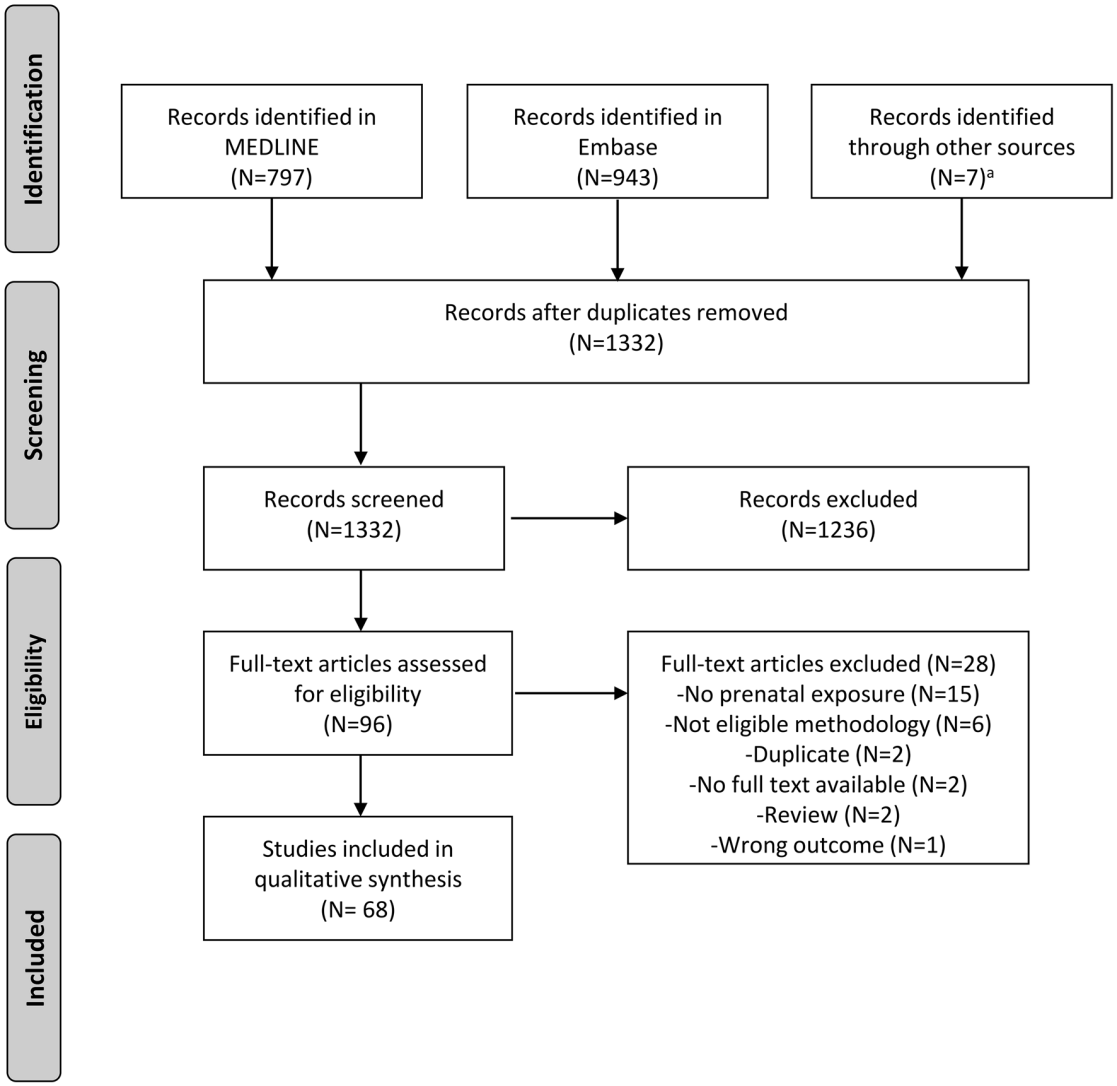
PRISMA Flowchart of study selection. ^a^Studies were identified by checking citing and cited studies of the included papers

The search identified 1332 unique studies, of which 1236 were excluded after title and abstract screening. The full-text of the remaining 96 articles was assessed on the eligibility criteria (Fig. 1). After full-text screening, 68 articles were included. Studies investigated the following prenatal factors: maternal age (N = 30), paternal age (N = 22), birth order (N = 15), season of birth (N = 16), place of birth (N = 13), prenatal influenza pandemic exposure (N = 1), prenatal famine exposure (N = 1), birth characteristics (N = 3) and indicators for prenatal sex hormone exposure (digit ratio or by comparing same- or opposite-sex twin pairs) (N = 4). Several studies investigated multiple prenatal factors.

**Fig. 2:**
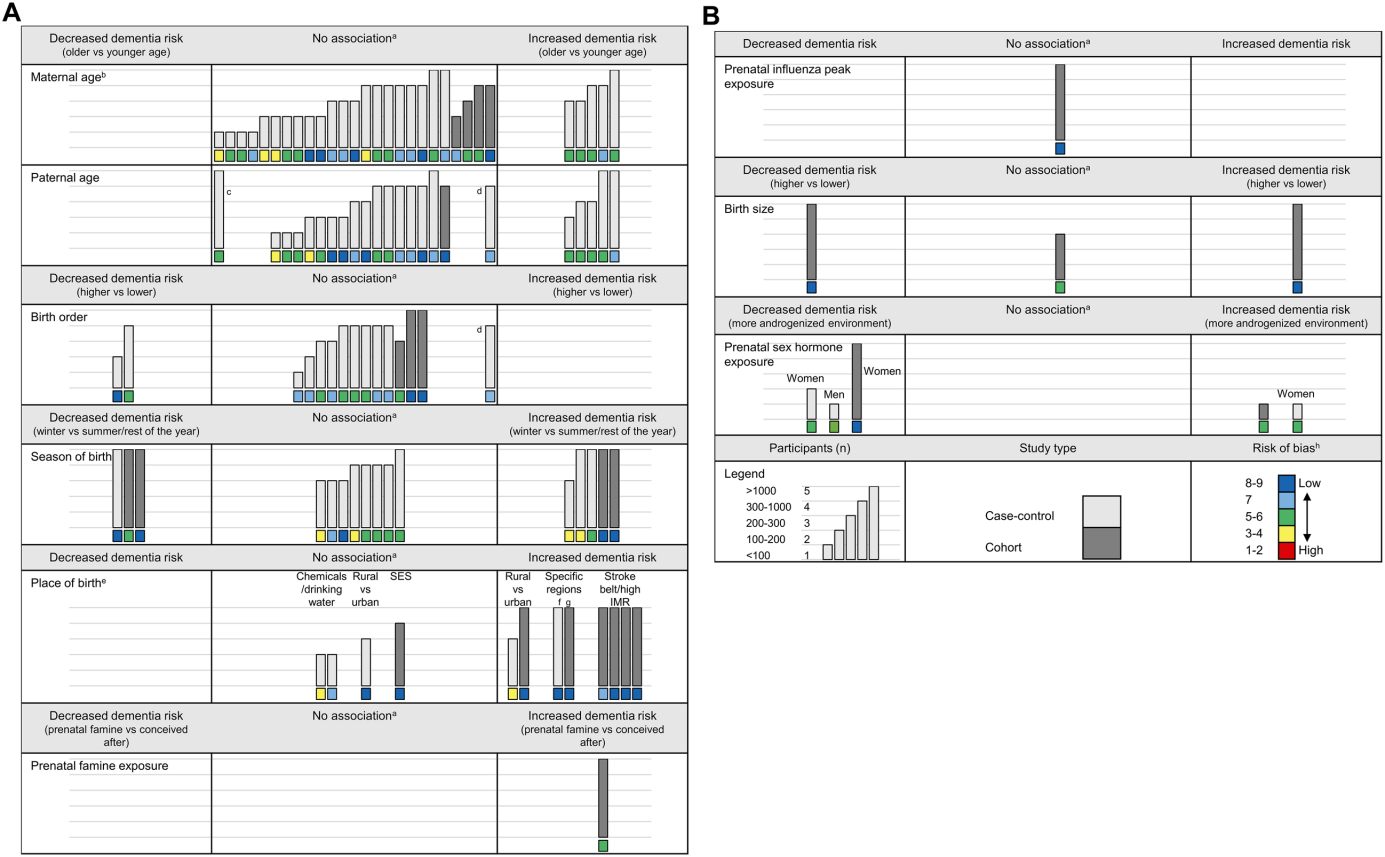
**a** and **b** Overview of included studies. Each bar represents a study and the height of the bar refers to the size of the study (see legend). The colors of the bars refer to case–control (light grey) and cohort (darker grey) studies and the colored boxes refer to the risk of bias scores (see legend). The placement of the bars (left, middle, right), refers to the direction of the crude associations, or the fully adjusted associations if the study did not report the results of a crude model. *SES* Socioeconomic status, *IMR* Infant mortality rate. ^a^No statistical significance at the p = 0.05 level. ^b^The study by Cohen et al. was not included in this figure as they did not provide any statistical results. ^c^The study was close to statistical significance at the p = 0.05 level in the direction of a decreased dementia risk with older fathers. ^d^The study was close to statistical significance at the p = 0.05 level in the direction of an increased dementia risk with older fathers and higher birth order. ^e^We did not report the results of Baker et al.as the direction of their association regarding rural compared to urban birth was unclear. ^f^Two Newfoundland (Canada) regions had high dementia mortality rates. There was an excess of persons with dementia from the north of one region which coincided with the lowest pH, highest concentration of aluminum and highest water color measurements in drinking water (indicating lower water quality) of the region. ^g^Southern Italy compared to north-western Italy. ^h^For retrospective cohort studies the maximum score was eight, therefore, one point was added to the risk of bias score

In the majority of studies (75%), a clinical diagnosis of dementia, mostly AD, was used as an outcome. In the remaining studies, the outcome was dementia as a cause of death (registry data), autopsy proven dementia or a combination of either of these and clinical diagnoses of dementia. Below, we describe the results per prenatal factor. An overview of the results is provided in Fig. 2 (more detailed information, risk of bias results, study characteristics and subgroup results can be found in Online Resources 3–5). We did not perform a meta-analysis due to the large heterogeneity (e.g. in methodology or setting) among the included studies investigating similar exposures and some studies did not report numerical results.

### Parental age and birth order

Maternal age, paternal age and birth order are correlated and were often studied together. We included 35 unique studies investigating associations between maternal age, paternal age and/or birth order and dementia (Fig. 2; Online Resource 4).

Maternal age was studied in 30 studies (26 case–control, 4 cohort). No statistically significant differences in maternal age between AD [33–52], vascular dementia [34], multi infarct dementia [53] or dementia [54] cases and controls were reported in 22 case–control studies, and 4 cohort studies (AD [55, 56], dementia [57, 58]). Cohen et al. did not report any statistical results, but did report a more than eight year higher mean maternal age for cases compared to controls [43]. Five studies, all with a case–control design, reported a statistically significant higher maternal age among AD cases compared to controls[53, 59–62].

Twenty-two studies additionally included paternal age (21 case–control, 1 cohort). No statistically significant differences in paternal age were found in 17 case–control studies regarding AD [35, 37–44, 46, 47, 51, 52, 61, 62], vascular dementia [34] or dementia [54] diagnosis and 1 cohort study regarding AD [56]. Two of these studies reported differences which came close to statistical significance at the p = 0.05 level, one reporting higher AD risk for those with older fathers (OR 4.50 p = 0.06) [60], while the other reported lower AD risk for those with older fathers (OR 0.88 95% CI 0.76–1.01) [33]. Five case–control studies reported a statistically significant higher paternal age among AD cases compared to controls [34, 46, 53, 59, 62].

Birth order was included in 15 studies (12 case–control, 3 cohort). In total, 10 case–control studies did not report a statistically significant difference in birth order among AD cases and controls[35, 50–52, 60–65]. One of these studies looked at birth order in twin-pairs. Additionally, the three cohort studies did not observe an association between birth order and dementia diagnosis [58, 66, 67], and also no association between the age difference with the next older sibling and risk for dementia [58]. However, the results of one of these studies came close to statistical significance, with higher odds of AD for individuals born as the fourth child or later (OR 1.88 p = 0.06) [60]. Two case–control studies showed a statistically significant association between birth order and AD with higher AD risk in those with lower birth order [39, 44]. One of these studies additionally reported a significantly lower maternal and paternal age for cases compared to siblings, they did not observe this difference compared to spouses or community control groups, therefore, this may be due to the lower birth order they observed for cases [44].

To summarize, the majority of studies did not report a statistically significant association between parental age or birth order and dementia. Regarding both maternal and paternal age, 5 studies (16.6% of maternal age studies; 22.7% of paternal age studies) reported an association with AD, all in the direction of older parental age being associated with a higher risk of AD. Furthermore, 2 of 15 birth order studies (13.3%) reported higher odds of AD for those with lower birth order. We scored 18 out of 35 (51.4%) parental age and birth order studies as having high or intermediate risk of bias (Online Resource 3). Studies had the highest risk of bias regarding non-response rate and ascertainment of the exposure. The non-response rate was often not clearly reported. For the ascertainment of exposure, many studies adhered to an interview conducted by an interviewer not blinded to case–control status. However, as parental age and birth order are relatively straightforward to measure, the risk of bias due to lack of blinding is likely not high. There were four studies with potential overlap in the participants included in their studies [33, 35, 52, 59].

### Season of birth

We included 16 unique studies investigating the association between season of birth and dementia (12 case–control, 4 cohort studies) (Fig. 2; Online Resource 4).

Eight case–control studies defined season of birth as birth in one of the quarters of the year (January–March; April–June; July–September; October–December). No statistically significant differences between the quarter of birth in AD cases and controls were observed in six studies [61, 68–72]. Four studies (additionally) investigated cyclic trends without comparison to a control population and found no peak period of births related to later AD [68, 69, 71, 73]. Two studies did observe significantly more births in the first quarter (winter) [73] or the first four months of the year (winter) [74], respectively, compared to birth in the rest of the year among AD cases compared to controls.

In six studies (Three case–control, three cohort), season of birth was defined as birth in the meteorological seasons (Winter: November or December–February; Spring: March–May; Summer: June–August; Autumn: September–October or November). Two case–control studies did not observe a seasonal pattern among cases and controls [36, 75]. However, in one study there was a tendency towards higher mean birth frequencies in the fall for vascular dementia patients and in winter for AD patients [75]. The study by Tolppanen et al., reported that AD cases were more often born in summer (specifically July), although the absolute difference was only 0.5% [76]. Two cohort studies observed a lower odds of developing dementia for those born in winter compared to those born in summer [77, 78]. In contrast, the cohort study by Mooldijk et al. observed a higher dementia risk, especially for AD, for participants born in winter and fall compared to those born in summer. The increased risk was most pronounced in those born in colder winters [79].

Vezina et al. looked at the specific months of birth and found significantly more births in February (winter) among AD cases compared to one of their control groups (based on universal healthcare data) and significantly less births among AD cases in May compared to both control groups [80]. When looking at daily birth distributions, the lower number of births in May remained statistically significant and extended to some days in June [80]. Hsu et al. showed statistically significant periodicity (by month of birth) for AD, with a relatively higher risk for those born in the autumn or winter months [81].

To summarize, eight studies did not report a statistically significant association regarding season of birth, three studies reported an increased dementia risk for those born in summer compared to birth in the winter or the rest of the year and five studies reported a higher risk of dementia for those born in winter compared to birth in summer or the rest of the year. We scored 10 out of 16 (62.5%) season of birth studies as having high or intermediate risk of bias. Risk of bias was highest regarding the non-response rate, ascertainment of the exposure, representativeness of the cases and not using the same method of exposure ascertainment for cases and controls. As season of birth is solely based on birthdate, flaws in the ascertainment of exposure may not be a major risk of bias.

### Place of birth

We included 13 studies investigating associations between place of birth and dementia (Fig. 2; Online Resource 4) (6 case–control studies, 7 cohort studies). Studies looked at place of birth in relation to being born in a rural versus urban area, being born inside or outside the stroke belt, being born inside or outside a high infant mortality state, place of birth related to the chemical composition of the soil or drinking water, birth county socioeconomic status and being born in specific areas of Italy.

Four studies investigated rural versus urban place of birth (three case–control, one cohort). Prince et al. found no place of birth effect on dementia [36]. Jean et al. found an increased risk for AD for rural born individuals and a decreased risk for AD for urban born individuals compared to a reference population [82]. The results of one particular study were unclear, as they reported a higher odds of AD for those born in an urban versus rural environment in the text, but reported an opposite effect in their tables [83]. In one cohort study investigating birth in a rural area, town or city, a linear trend was statistically significant with the highest risk for dementia among those born in rural places [84].

One study observed an increased dementia risk and two studies observed an increased dementia and AD mortality risk, respectively, for those born in high stroke mortality states in the United States compared to other states.[85–87].One study found that birth inside versus outside the highest quartile infant mortality rate states in the US was associated with higher dementia risk for African American people but not for white people in a fully adjusted model [88].

Three studies evaluated associations between the chemical composition of soil or drinking water at the place of birth and dementia (as a cause of death) [89], AD [90] and presenile AD [48]. Frecker et al. compared dementia mortality rates among different birth regions in Newfoundland, Canada [89]. Two regions had high dementia mortality rates. In one region dementia cases were further evaluated. There was a statistically significant excess of persons with dementia from the north of this region which coincided with the lowest pH, highest concentration of aluminum and highest water color measurements in drinking water (indicating lower water quality) of the region. However, two other areas in the region also had high aluminum concentrations but no corresponding dementia increase. In another study by Emard et al. no geochemical element (including aluminum) explained the spatial distribution of births of AD cases. Nevertheless, AD cases were more likely to have higher instead of lower lead, manganese and iron concentrations at their birthplace than the average municipal concentration [90]. Furthermore, Forster et al. found that presenile dementia cases were not born more often in areas with on average higher aluminum concentration in drinking water compared to controls [48].

Wilson et al. observed no statistically significant association between birth county socioeconomic level and AD [91]. Furthermore, Guaita et al. investigated associations between birth in specific parts of Italy and risk for overall dementia, AD or vascular dementia. Subjects born in southern Italy had significantly higher risk for both overall dementia and vascular dementia compared to those born in north-western Italy [92].

In summary, nine studies reported associations between place of birth and dementia. For one study the direction of the association was not clearly reported regarding rural or urban birth. In the remaining eight studies the risk of dementia was higher for those born in a rural versus urban environment (N = 2), in southern Italy compared to north-western Italy (N = 1), in two Newfoundland regions compared to the other regions (N = 1; of which some might be explained by poor drinking water quality) or being born inside compared to outside the stroke belt (N = 3) or a high infant mortality area (N = 1) respectively. Four studies did not report a statistically significant association between rural vs urban birth (N = 1), Birth county socioeconomic level (N = 1) and the chemical composition of the soil or drinking water (N = 2) and dementia. Only 3 out of 13 (23.1%) studies regarding place of birth had intermediate or high risk of bias. Similar to the previous exposure categories, studies scored the lowest on non-response rate and ascertainment of the exposure.

### Other prenatal factors

Six studies were performed regarding other prenatal factors (Fig. 2; Online Resource 4). Cocoros et al. investigated associations between in utero exposure to the 1918 influenza pandemic and dementia of any type and specific dementia types (AD, vascular dementia, other dementia) among individuals aged 62 years and older. Only the association between prenatal influenza pandemic exposure and ‘other dementia’ was statistically significant, although, the incidence rate ratio was only minimally increased [93].

Kang et al. observed that men and women prenatally exposed to the Chinese famine had a statistically significant higher risk for dementia compared to a group conceived after the famine. However, there was no significant difference compared to a group born before the famine [94].

Three studies evaluated the second to fourth finger length ratio (2D:4D) as an indication of prenatal sex hormone exposure. Lenz et al. found that normalized 2D:4D values were negatively associated with normalized deaths from AD and other dementias in men and women comparing averages of different countries, indicating a higher risk for prenatally more androgenized individuals [95]. Vladeanu et al. observed higher 2D:4D ratios in men diagnosed with AD compared to controls, indicating an association between lower levels of prenatal testosterone and higher levels of prenatal estrogen exposure and AD risk. For women, the opposite was observed, with lower 2D:4D ratios observed in women diagnosed with AD compared to controls [96]. Jiang et al. observed no difference in 2D:4D ratios comparing men diagnosed with dementia to controls. Women with a dementia diagnosis had higher 2D:4D ratios compared to controls, suggesting that a more feminine prenatal environment may predispose women to dementia [97]. In addition to finger length ratio, one study compared dementia risk in same- and opposite-sex dizygotic twin pairs as a marker for the prenatal hormone environment [98]. Women from opposite-sex pairs had significantly lower dementia risk than women from same-sex pairs, but only after age 70. In men, the risk for dementia did not differ among the different twin types. These findings suggest a lower dementia risk in women after exposure to a relatively masculine prenatal hormone environment [98].

The studies regarding prenatal famine exposure and digit length ratio had major limitations in their study design and method of analysis, and were thus scored as having high/intermediate risk of bias. The studies regarding twin-type and 1918 influenza pandemic exposure were scored as having low risk of bias.

### Birth characteristics

Three cohort studies were identified regarding size at birth and dementia [65, 99, 100] or AD specifically [100] (Fig. 2; Online Resource 4). Mosing et al. evaluated multiple measures of size at birth in twins: birth weight, head circumference at birth and birth length. Furthermore, they included gestational age and calculated birth weight and birth length relative to gestational age [65]. No statistically significant associations with dementia were found regarding being small for gestational age, length at birth, head circumference or gestational age. An increased risk for dementia was observed for low birth weight babies compared to normal weight babies. Furthermore, birth weight, head circumference and birth weight adjusted for gestational age (and sex) were inversely associated with dementia risk [65]. Syddall et al. found that higher birth weight was associated with higher all cause dementia mortality in men and higher AD mortality in women, respectively [100]. Matsushima et al. did not find an increased risk for dementia for low birth weight (< 2500 g) babies including premature babies [99]. Thus, in summary, one study did not report an association between birth weight and dementia and two studies did report an association between birth weight and dementia, however these associations were in opposite directions. Only the study by Matsushima et al. was scored as having intermediate/high risk of bias while the other studies had low risk of bias.

## Discussion

In this systematic review of 68 studies, we found evidence for associations between markers of a suboptimal prenatal environment and risk for dementia, with the most consistent evidence for an association between place of birth and risk for dementia. In general, birth in a less optimal environment (e.g. rural vs urban environment or birth inside vs outside high stroke mortality areas) was associated with a higher risk for dementia. An increased risk for dementia was also observed in those with either low or high birth weight and those prenatally exposed to famine. In general, these studies had relatively low risk of bias. Furthermore, there was some evidence for an association between lower 2D:4D ratios and higher risk for dementia, although the direction of the association was opposite for men in one study. There was little or highly inconsistent evidence for season of birth, prenatal influenza pandemic exposure, parental age and birth order being risk factors for dementia.

### Place of birth

Our findings regarding place of birth as being of significance for later risk for dementia are in line with a large body of evidence showing associations between place of birth and a child’s opportunities, health and wellbeing in later life, as well as life expectancy [101–106]. Measures of place of birth can be powerful indicators of the social and physical conditions that surround birth [88]. Place of birth may, for instance, relate to parental SES, maternal stress and maternal health behaviors such as smoking and poorer nutrition. In addition, place of birth could relate to environmental factors like poor air quality and toxins in the soil or water. These negative social and physical conditions could directly influence the risk for dementia by negatively affecting the prenatal and early life conditions in which the brain develops, limiting the growth and development of the brain. Prenatal air pollution has, for instance, been associated with markers of placental growth and function [107]. Diminished placental function limits the supply of oxygen and nutrients and thereby limits brain growth. Limited brain growth in early life may affect the brain’s reserve capacity in later life. A larger brain has been suggested to function as a buffer against the effects of age-related pathological damage to the brain as there is more tissue to lose [28]. Indeed, white matter microstructure, brain morphology and cognitive function in childhood have been associated with prenatal air pollution and it can be speculated that, on the long-term, this could impact the risk for dementia [108–110].

Furthermore, place of birth (irrespective of adult residence) can influence health behaviors (e.g. smoking), educational attainment and SES, and thereby affect dementia risk, for instance via the childhood social environment [85, 111]. Additionally, place of birth has been associated with risk for developing cardiovascular disease and diabetes, which are known risk factors for dementia [28, 112, 113]. Men and women born in the stroke belt in the US are, for instance, known to have an increased risk for cardiovascular disease. The same has been observed for those born in northern counties, industrial towns and Wales in a study conducted in England and Wales [85, 114]. Place of birth may thus indirectly increase the risk for dementia via increased prevalence of CVD and diabetes.

Place of birth may be related to adult residence, however, associations between place of birth and adult health have been shown to persist even when people moved to other areas later in life and are thus specific to early-life [85, 114]. Two studies in this review did include adult stroke belt residence in their model with dementia to account for later-life exposure. In one study this did not alter their results, suggesting an independent effect of birth in the stroke belt on the development of dementia [86]. In another study, adult stroke belt residence seemed to partly mediate the effects of birth in the stroke belt, however, selective migration outside the stroke belt by relatively healthier people may have produced bias [87]. Unfortunately, other place of birth studies did not explore mediation by adult residence. Furthermore, potential confounding and other mediating factors like parental SES and educational attainment were not investigated in most studies related to place of birth. Therefore, much uncertainty remains regarding the interpretation of these findings.

### Birth weight

Two studies observed opposite associations with either lower or higher birth weight being associated with an increased risk for dementia. Birth weight has been described as a risk factor for many chronic disorders and is known to often follow a u-shaped curve with both lower and higher birth weight resulting in increased risk for adverse health outcomes. A u-shaped association has, for instance, been established between birth weight and T2DM [5]. This could potentially explain the opposite associations we observed and would suggest that both lower and higher birth weight are an indication of less optimal developmental circumstances. However, the study reporting an association between lower birth weight and dementia only included twins, making it difficult to directly extrapolate their results to other populations.

### Prenatal famine exposure

One study observed that men and women prenatally exposed to the Chinese famine had significantly higher risk for dementia compared to a group conceived after the famine [94]. These findings are in line with the literature, prenatal famine exposure has previously been associated with smaller brain size, older appearing brains and worse cognitive function [16–22, 94]. The Chinese famine study was however hampered by not adequately correcting for age differences across the groups.

### Prenatal sex hormone exposure

Two studies reported an association between lower 2D:4D ratios (indicating higher levels of prenatal testosterone and lower levels of prenatal estrogen) and higher risk for dementia, in one study the association was opposite for men. In contrast, a third study regarding 2D:4D ratios, observed no difference in men, but observed higher 2D:4D ratios (indicating a more feminine prenatal environment) in women with dementia compared to controls [97]. In line with these findings, in a study including twins, women from opposite-sex pairs had significantly lower dementia risk than women from same-sex pairs after age 70. In men, the risk for dementia did not differ among the different twin types. This again suggests a higher dementia risk in women after exposure to a relatively feminine prenatal hormone environment [98]. The direction of effects was thus not completely consistent, possibly due to methodological problems and high risk of bias within some of these studies. Research regarding adult sex hormones and age-related cognitive decline and dementia has yielded mixed results [96]. One study regarding 2D:4D ratios found a statistically significant correlation between 2D:4D ratio and Mini-Mental State Examination scores in women, suggesting that lower prenatal exposure to testosterone is associated with lower cognitive decline [115]. Potential mechanisms behind associations of 2D:4D ratios with cognitive decline and dementia are currently not well understood.

### Season of birth, parental age and birth order

There was little evidence for a consistent role of season of birth, parental age and birth order in the risk for dementia. In general, the quality of these studies was limited and their methods and setting were very heterogeneous, possibly explaining their mixed results. Many studies failed to account for relevant covariates or select a control group adequately representative for the population where the cases originated form. Most studies regarding parental age included maternal or paternal age as linear continuous measure, thereby potentially overlooking a u-shaped association, as both younger and older parental ages could result in higher risk for adverse outcomes like dementia [116].

As the studies that did show an association between paternal and maternal age and dementia reported associations in the same direction (i.e. higher risk for those with older mothers and fathers), advanced parental age may play a role in the risk for dementia, perhaps dependent on other factors. Advanced maternal age is a risk factor for gestational diabetes mellitus, gestational hypertension, preeclampsia, small for gestational age offspring, spontaneous late preterm delivery and cesarean section, all of which are conditions reflecting a less optimal prenatal environment and that may negatively affect brain development [117]. Furthermore, a potential mechanism for both maternal and paternal age effects could be via genetic mutations and epigenetic changes [34, 118]. Independent effects of paternal age on offspring health have been observed, for instance in relation to mental health problems and lower intelligence in offspring [118].

Season of birth is a very broad and indirect measure of the prenatal and early-life environment and could represent numerous exposures (e.g. infection pressure, scarcity of resources, in utero vitamin D exposure) dependent on the specific setting (e.g. latitude, living circumstances). Our results showed no association between season of birth and dementia in half of the studies. Five included studies, including one based on 29 million people, reported a higher dementia risk for those born in winter. These findings are in line with the observation is a large study from Europe that provided evidence for an association between winter birth and lower cognitive function in later life [119]. However, three relatively large season of birth studies included in our review (all including more than 100,000 participants), conducted in Finland, Germany and China respectively, all observed an increased risk for dementia for those born in summer. We were not able to detect any differences in setting explaining these opposite associations.

As the effect of season of birth is highly dependent on setting and place of birth, it is possible that it does play a clear role in the risk for dementia in specific areas across the globe with larger weather differences or major differences in food availability across the seasons. An example of such a location could be rural Gambia, were many associations between season of birth and offspring health have been established [120]. Most season of birth studies in this review were not conducted in locations where there are such large differences.

### Strengths and limitations

This systematic review provides an extensive overview of the literature regarding prenatal factors and dementia. By including studies irrespective of the specific prenatal exposure or dementia subtype, we made an effort to include all available literature regarding this topic. Other strengths of this study included the comprehensive search strategy and the independent data extraction.

This systematic review has limitations of which some are a consequence of the available literature and the quality of included studies. We included some prenatal factors for which it is difficult to disentangle prenatal and (early) postnatal circumstances. For instance, place of birth is highly correlated with childhood residence. However, place of birth is likely still a good representation of the early life environment and does show the importance of factors playing in early life.

Furthermore, many included studies were of low quality and had intermediate or high risk of bias. Methods of included studies were heterogeneous, making it difficult to directly compare study results. We were unable to perform a meta-analysis and estimate effect sizes due to this heterogeneity among studies. Additionally, many studies did not report complete numerical results. The consequential limitation of our review is the focus on statistically significant versus non-significant results of individual studies instead of the pooled results of similar studies. To provide some additional context to our results, we did make an effort to summarize study findings in relation to study-type, study size and risk of bias score in Fig. 2. Another limitation is that we used a summary/cut-off score to summarize the risk of bias results, as not all sections of the Newcastle Ottawa Scale are equally important. We did describe the largest risks of bias in the results section and provided the full risk of bias evaluation in Online Resource 3.

Lastly, although we adhered to an extensive search strategy and checked citing and cited studies, it is possible we have missed articles in our review and the possibility of publication bias remains. Unfortunately, we were not able to investigate publication bias with the help of a funnel plot.

In addition to the limitations of our review, there are some general limitations that apply to most studies included in this review. For instance, dementia diagnosis typically occurs when pathology overwhelms function and a diagnostic threshold is reached [28]. Factors like SES and educational level may influence the timely recognition and diagnosis of dementia and could thereby bias the results presented in this review, with missing or delayed diagnoses in lower SES individuals. Many studies did not report or account for SES in their analysis and we were therefore not able to further investigate its effects. As adverse prenatal factors may lower an individual’s SES (e.g. lower education or work participation) [121, 122], this may have led to an underestimation of the associations between adverse prenatal factors and an increased risk for dementia.

Furthermore, many studies accounted for age and sex, however, residual confounding may remain. Other (unmeasured) prenatal factors may (partly) explain the observed associations with dementia for some prenatal factors included in this review. For instance, parental SES may partly explain the associations regarding place of birth and dementia. As often no other prenatal factors were measured, the precise role of other factors can only be speculated on.

### Recommendations for future research

Our results have shown an increased risk for dementia for those born in less optimal environments. Nevertheless, the exact exposures or circumstances that may be detrimental were difficult to establish given that most investigated exposures were indirect measures of the prenatal environment. Future studies focusing on clear, direct exposures linked to a less optimal environment such as maternal smoking, alcohol use and stress may provide valuable information underlying pathways and potential targets for societal improvement. Furthermore, to answer questions about specific critical time widows in the prenatal period for the development of dementia, prenatal factors that play a role in a short time frame are especially of interest.

By taking on a life-course approach, cohort studies can make an effort to improve the understanding of pathways between prenatal factors and risk for dementia. It would be of great value to include mediating factors and other covariates in future studies to understand the role of factors like education, SES and comorbidities like cardiovascular disease, T2DM, depression and anxiety in the association between prenatal factors and dementia. Understanding pathways between prenatal factors and dementia will help us to better understand who is at the highest risk for developing dementia, how we could potentially intervene to prevent or delay dementia as well as shape future research strategies. In addition, trial studies could be of importance to evaluate if those exposed to adverse prenatal circumstance benefit more from strategies to lower risk for dementia compared to those not exposed to adverse prenatal circumstance, for instance with strategies of cognitive stimulation, exercise and healthy nutrition [15].

In this review, we focused on dementia diagnosis, regardless of age of onset. Age of onset may also be an important outcome measure since prenatal exposures may not only affect risk for dementia diagnosis, but also age at symptom onset and age at diagnosis. Unfortunately, the majority of included studies did not specifically report age of dementia onset as an outcome. Age of onset would be an important outcome to consider in future studies as earlier age of dementia onset would lead to a major increase in total disease burden.

As setting up new prospective cohort studies from preconception up to old age will not give us results in the near future, future studies could focus on retrospective and well registered measures of the prenatal environment. Currently ongoing cohort studies in which participants have reached an old enough age to develop dementia could include dementia in new follow-up rounds. Alternatively, studies could focus on earlier occurring outcomes predictive of future dementia risk, including (self-perceived) cognitive decline and early brain markers of dementia.

## Conclusion

We identified a large body of literature, showing that some prenatal factors are associated with later risk for dementia. Our findings suggest that prenatal factors that are markers for a suboptimal prenatal environment, especially those related to place of birth, are associated with an increased dementia risk. As this association may be confounded by factors such as parental socioeconomic status, more research is needed to examine the potential causal role of the prenatal environment in dementia.

## Supplementary Information

Below is the link to the electronic supplementary material.

Supplementary file1 (PDF 81 KB)

Supplementary file2 (PDF 204 KB)

Supplementary file3 (PDF 659 KB)

Supplementary file4 (PDF 858 KB)

Supplementary file5 (PDF 326 KB)

## Data Availability

All relevant data are within the manuscript and its Supporting Information files. The corresponding author may be contacted for further information.
