# Supplementary material for: Do prenatal factors shape the risk for dementia?: A systematic review of the epidemiological evidence for the prenatal origins of dementia
Source: Soc Psychiatry Psychiatr Epidemiol. 2023 Apr 8;60(5):977–91. doi: 10.1007/s00127-023-02471-7 (PMC12119685; doi:10.1007/s00127-023-02471-7)
Supplement: Supplementary file 2 — Supplementary file2 (PDF 204 KB) [file 127_2023_2471_MOESM2_ESM.pdf]

Database(s): **Ovid MEDLINE(R) and Epub Ahead of Print, In-Process, In-Data-Review & Other Non-Indexed Citations and Daily** 1946 to November 23, 2022

#	Searches	Results
1	dementia/ or alzheimer disease/ or dementia, vascular/ or dementia, multi-infarct/ or frontotemporal dementia/ or lewy body disease/	172433
2	(alzheimer* or MGAD or ((senile or presenile or PSD) adj2 AD) or lewy body).tw,kf. or (dement*.tw,kf. not dement* pr?ecox*.ti.)	267963
**3**	**1 or 2 [Alzheimer / Dementia]**	287112
4	prenatal exposure delayed effects/ or maternal exposure/ or paternal exposure/ or prenatal nutritional physiological phenomena/ or obesity, maternal/	43339
5	fetal development/ or fetal organ maturity/	11528
6	fetal weight/ or gestational age/ or gestational weight gain/ or maternal-fetal exchange/ or pregnancy outcome/ or pregnancy, high-risk/ or maternal age/ or parity/ or paternal age/ or exp pregnancy trimesters/ or exp pregnancy, multiple/ or sexual development/ or sex determination processes/ or apgar score/	252418
7	perinatal care/ or preconception care/ or prenatal care/	38533
8	pregnancy complications/ or exp diabetes, gestational/ or exp fetal diseases/ or exp hypertension, pregnancy-induced/ or exp fetal membranes, premature rupture/ or exp obstetric labor, premature/ or exp placenta diseases/ or pregnancy complications, cardiovascular/ or pregnancy complications, hematologic/ or pregnancy complications, infectious/ or pregnancy complications, parasitic/ or pregnancy in diabetics/ or prenatal injuries/	309993
9	exp birth weight/ or exp infant, low birth weight/ or exp infant, premature/	124092
10	(cephalometry/ or leg/ag or head/ah) and (exp infant/ or fetus/ or parturition/ or birth rate/ or live birth/ or ((birth not (birth adj6 (cohort* or defect* or control or match* or adjust*))) or births* or childbirth* or born or early-life or parturit*).tw,kf.)	4319
11	birth certificates/ or birth intervals/ or birth order/ or exp birth setting/ or ((seasons/ or residence characteristic/ or geography/ or rural population/ or suburban population/ or urban population/ or rural health/ or suburban health/ or urban health/ or urbanization/ or *"emigrants and immigrants"/ or life change events/ or exp nutrition disorders/) and (parturition/ or birth rate/ or live birth/ or ((birth not (birth adj6 (cohort* or defect* or control or match* or adjust*))) or births* or childbirth* or born or early-life or parturit*).tw,kf.))	42500
12	"influenza pandemic, 1918-1919"/ or ((china/ or epidemics/) and (malnutrition/ or starvation/ or famine/ or hunger/)) or ((socioeconomic factors/ or economic status/ or exp educational status/ or employment/ or unemployment/ or income/ or occupations/ or exp poverty/ or social class/ or education/ or exp educational measurement/ or exp mining/ or exp occupational diseases/ or exp environmental pollution/ or environmental health/ or endocrine disruptors/ or exp environmental pollutants/ or exp pesticides/ or exp metals, heavy/ or exp particulate matter/ or occupational health/ or exp substance-related disorders/ or drinking behavior/ or alcohol drinking/ or exp "marijuana use"/ or exp smoking/ or exp "tobacco use"/ or exp disease outbreaks/ or infectious disease transmission, vertical/ or "influenza a virus"/ or influenza, human/ or cytomegalovirus infections/ or cytomegalovirus/ or exp flavivirus infections/ or exp flavivirus/ or famine/ or hunger/ or nutrition disorders/ or malnutrition/ or exp avitaminosis/ or starvation/ or overnutrition/ or obesity/ or obesity, abdominal/ or obesity, morbid/) and (parents/ or fathers/ or mothers/ or uterus/ or parturition/ or birth rate/ or live birth/ or ((birth adj2 (patient* or date or rate)) or	88764

	followed-from-birth or before-birth* or after-birth* or ("at-birth" not (life expectan* adj2 birth*)) or ((across-life or throughout-life or ((across or follow*) adj3 life course)) and (early or birth))).tw,kw. or ((f?etus* or f?etal) not f?etal bovine serum).mp.))	
13	fingers/ah, pa	3600
14	(intra-uterin* or intrauterin* or utero or antenat* or ante-nat* or prenat* or pre-nat* or perinat* or peri-nat* or preconceptional* or pre-conceptional* or periconcept* or peri-concept* or postconcept* or post-concept* or pre-birth* or prebirth*).tw,kf.	312615
15	(gestational or (SGA adj3 (infant* or neonat* or newborn* or neo-nat* or new*-born* or pregnan* or gestat* or birth* or weight*))).tw,kf.	135238
16	(DOHAD* or FOAD*).tw,kf. or (development* adj2 origin* adj3 (health* or diseas*)).tw,kf,jw.	2814
17	(((f?etal or f?etus* or embryo* or pregnan* or early or developmental or neurodevelop* or nutrition*) adj9 programming) or ((f?etal or hormon*) adj3 priming)).tw,kf.	7195
18	(f?etal adj3 (period* or life or expos* or famine or nutri* or undernutr* or malnutr* or overnutrit* or obes* or stress* or origin* or start* or begin* or (root* not dorsal root*) or antecedent* or hypothes*)).tw,kf.	21255
19	(((f?etal or f?etus*) adj3 (growth or compromis* or preterm or pre-term* or prematur* or pre-matur*)) or IUGR* or FGR* or SFGR* or SIUGR* or previab* or pre-viab* or "before viab*" or (placent* adj3 (insufficien* or d*sfunct*))).tw,kf.	36719
20	((PROM not prospective memor*) or PPROM* or EPPROM*1 or ((prematur* or pre-matur* or i?matur* or preterm* or pre-term*) adj6 (ruptur* or labor or labour or labo?ring or contraction* or birth or births or baby or babies or childbirth* or delivery or deliveries or parturit* or infant* or neonat* or neo-nat* or new*born* or new*-born*)) or VPTB* or MPTB* or prelabo?r or pre-labo?r).tw,kf.	138059
21	((early adj (labor or labour or parturit* or deliver* or birth or births or childbirth*)) or ((gestat* or age) adj2 ("at birth" or "at deliver*"))).tw,kf.	11567
22	(((birth or births or baby or babies or neonat* or neo-nat* or new*born* or new*-born* or f?etal) adj3 (underweight* or weight* or size or length* or height* or BMI or body mass*)) or birthweight* or LBW* or VLBW* or ELBW*).tw,kf.	104989
23	((((head or skull*) adj2 (circumfere* or size* or small*)) or ((leg or limb*) adj2 (size* or length* or small*)) or cephalometr*) adj9 (birth or births or baby or babies or neonat* or neo-nat* or new*born* or new*-born* or postnat* or postnat* or early-life* or infant* or child*)).tw,kf.	5271
24	(interpregnan* or inter-pregnan* or ((pregnan* or birth*) adj3 interval*)).tw,kf.	4777
25	(HELPP or preeclam* or eclamp*).tw,kf.	42483
26	(((maternal or mother* or gestat* or pregnanc* or pregnant or gravid* or trimester*) adj4 (expos* or radiat* or irradiat* or addict* or substance abus* or smoking or tobacco or cigarett* or nicotin* or alcohol* or caffein* or drug* or psychotrop* or narcotic* or mari*uana or hash* or cocain* or amphetamin* or amfetamin* or MDMA or opium or opiat* or heroin* or GHB or ketamin* or LSD or antidepres* or anti-depres* or SSRI* or SNRI* or (serotonin* adj3 reuptake inhibitor*) or cipramil or lexapro or prozac or fevarin* or seroxat or zoloft or cymbalta or efexor or effexor or pristiq or fetzima or ixel or savella or milnacipran or monoamine oxidase inhibitor* or MAOIs or MAO-inhibitor* or analgesic* or painkiller* or anti-inflammator* or aspirin* or ((cox* or cyclooxygenase or cyclo-oxygenas*) adj3 (inhibitor* or block* or antagon*)) or coxib* or celecoxib or diclofenac or	217992

	ibuprofen or indomethicin* or naproxen or acetaminophen* or acetylsalicylic or aspirin* or antidiabetic* or metformin* or cortico* or cortisol or hydrocort* or steroid* or glycosteroid* or glycocortico* or dexameth* or prednis* or betameth* or infect* or influenz* or virus* or viral or CMV or cytomegalovir* or toxin* or heavy-metal* or lead or Pb or mercury or Hg or arsen* or cadmium or chromium or Cr or Nickel or Ni or pollut* or chemic* or endocrine disrupt* or BPA or BPAs or bisphenol* or PFOA or PFOAS or PFTE or teflon or perfluoro* or per-fluoro* or polychlor* or PCB or PCBs or biphenyl* or phalat* or perchlorat* or plastic* or pesticid* or asbest* or solvent* or thinner* or nutrit* or undernutrit* or malnutrit* or famine or hunger or obes* or overnutrit* or supplement* or vitamin* or multivitam* or vit-D or vit-B12 or vit-B6 or vit-C or vit-A or vit-E or retinol or ascorbic* or ascorbat* or tocopherol* or alphatocopherol* or tocotrienol* or cobalamin* or pyridoxin* or folic acid or folate or iron or calcium or polyunsaturat* or poly-unsaturat* or monounsaturat* or mono-unsaturat* or MUFA or MUFAs or PUFA or PUFAs or LCPUFA* or LCP or LCPs or docosahex?eno* or DHA or eicosapent?en* or icosapent?en* or EPA or omega-3* or omega-6* or omega3* or omega6* or n3 or n6 or n-3 or n-6 or linolenic or linolenate* or alphalinolen* or gammalinolen* or GLA or DGLA or arachidon* or ARA or weight* or BMI or body mass* or diet or diets or dietary or stress or diabet* or GDM or hyperten* or blood pressur* or an?emi*)) or (maternal adj2 offspring*)).tw,kf.	
27	((birth or births) adj2 (record* or chart* or certificat* or index)).tw,kf.	6487
28	((maternal or paternal or parent* or mother* or father*) adj3 (age or ages)).tw,kf.	41947
29	((born or birth or child* or infant*) adj3 (older or old or young*) adj2 (mother* or father* or parent*)).tw,kf.	4832
30	((maternal or mother*) adj3 (parity or multipar* or nullipar* or primipar*)).tw,kf.	5743
31	(((maternal or paternal or parent* or mother* or father*) adj2 educat*) or ((maternal or paternal or parental or parents or mother* or father*) adj5 (social status or socioeconomic* or economic* or SEP or cSEP or SES or cSES or income*1 or poverty or occupat* or employ* or unemploy* or mining or miners or coal or industr*))).tw,kf.	42472
32	(((condition* or characteristic* or circumstanc* or origin* or expos* or etiol* or aetiol* or (caus* not all-caus*-death*) or factor* or environment* or social status or socioeconom* or econom* or SEP or cSEP or SES or cSES) adj9 (birth or births or childbirth*)) not ((condition* or characteristic* or circumstanc* or origin* or expos* or etiol* or aetiol* or caus* or factor* or environment* or social status or socioeconom* or econom* or SEP or cSEP or SES or cSES) adj11 (birth cohort* or birth defect* or birth control))).tw,kf.	46129
33	(((condition* or characteristic* or circumstanc* or origin* or expos* or etiol* or aetiol* or caus* or social-status or socioeconom* or econom* or SEP or SES or cSES or program* or hunger or famine or nutritional deficien* or program* or event*) adj3 early-life) or (early life adj1 (factor* or variable* or environment* or precursor* or stress or residence)) or early life risk factor* or early exposur* or early famine or ((chinese or world war) adj3 famine) or spanish flu or 1918-influenza*).tw,kf.	13898
34	(((season* or winter* or summer* or autumn* or spring or springtime or month or quarter*) adj4 (birth or births or birthrate* or childbirth* or born)) or "time-of-birth*").tw,kf.	9778
35	(birth year not (match* adj6 birth year)).tw,kf.	1556
36	(((later or earlier or order) adj2 (birth or births or childbirth* or borns or born or sibling* or sibship*)) or ((first or 1st or second* or 2nd or third or	9644

	3rd or fourth or 4th) adj (born* or sibling*)) or ((number or size) adj2 (sibship* or sibling*)) or firstborn* or (each-additional adj2 (child or infant* or born))).tw,kf. not (twin or twins).mp.	
37	(((state or states or country or countries or county or counties or place or residenc* or region* or ((area or areas) not (surface adj3 area*)) or location* or overseas or foreign* or nativ* or rural or urban or cities or suburban or residential or industrial or mine or mines or coalmine*) adj3 ((birth not (birth adj6 (cohort* or defect* or control or match* or adjust*))) or births or childbirth* or born)) or (birthplace* not ((adjust* or match*) adj6 birthplac*))).tw,kf.	18371
38	(((second-to-fourth or index-to-ring) adj4 (finger* or digit*)) or ((digit or finger length*) adj3 (ratio* or 2d-4d or 2d?4d)) or ((2d-4d or 2d?4d or index finger* or ring finger*) adj4 (ratio or ratios or length* or male* or female* or gender))).tw,kf.	1334
39	((f?etal or fetus*) adj3 (hormon* or testosteron or androgen* or ?estrogen* or cortisol* or corticoster*)).tw,kf.	3257
**40**	**or/4-39 [prenatal origin/exposure]**	1039012
**41**	**3 and 40 [Alzheimer / Dementia -prenatal exposure/factors]**	1789
42	(exp animals/ not humans/) or (animal* or veterinar*).jw. or exp veterinary medicine/ or exp animal diseases/ or cattle/ or exp pregnancy, animal/ or exp animals, genetically modified/ or mice, knockout/ or exp animals, laboratory/ or animals, newborn/ or exp rodentia/ or (((primates or ape or apes or monkey* or baboon* or macaq* or pig or pigs or porcine or goat or goats* or sheep or lamb or lambs or ovine or cattle or bovine or cow or cows or horse or horses or mare or calve or calves or dog or dogs or canine or bitch* or cat or cats or feline or rodent* or rabbit* or mice or mouse or murine* or rat or rats or frog or frogs or zebra* or drosophila* or chick* or bee or bees).ti,ot. or (dam or dams or pups or pup or ewe or ewes or sow or sows or mice or mouse or murine or rat or rats or wistar or sprague or transgenic or Tx* or C57BL* or Balb-c or Balbc or wistar or sprague or dawley).tw,kw.) not (human* or patient* or infants or neonates or women or men).ti.) [animals not humans]	6607589
43	(down adj2 (syndrom* or model*)).ti,ot.	10821
**44**	**or/42-43 [exclusion animals; down syndrome model]**	6617321
**45**	**41 not 44 [human studies on AD/prenatal factors not Down syndrome]**	1338
46	(editorial or "systematic review").pt. or exp guideline/ or (committee or editorial or reply or guideline* or systematic or (case-report not case-report-survey) or two-cases).ti. or cochrane.jw. or ((review.pt. or case reports/ or (review or overview).ti. or (search* adj12 (literature* or ((electronic or medical or biomedical) adj3 database*) or exhaustive or systematic or medline or pubmed or embase or psychinfo or (CENTRAL and cochrane) or "Central Register of Controlled Trials")).tw,kf.) not (exp records/ or (case-control* or cohort* or retrospective or prospectiv* or crosssection* or cross-section* or population-based).ti,ot. or exp cohort studies/ or cross-sectional studies/ or case-control studies/ or ((chart* or record* or retrospectiv*) adj3 review*).tw,kf.)) [filter for original studies]	6184185
**47**	**45 not 46 [original human studies on AD/prenatal factors]**	815
**48**	**remove duplicates from 47 [original human studies on AD/prenatal factors - dedub]**	797

Database(s): **Embase Classic+Embase 1947 to 2022 November 23**

#	Searches	Results
1	dementia/ or alzheimer disease/ or diffuse lewy body disease/ or frontotemporal dementia/ or multiinfarct dementia/ or presenile dementia/ or senile dementia/	356501
2	(alzheimer* or MGAD or ((senile or presenile or PSD) adj2 AD) or lewy body).tw,kw. or (dement*.tw,kw. not dement* pr?ecox*.ti.)	374579
**3**	**1 or 2 [AD]**	437395
4	maternal exposure/ or paternal exposure/ or perinatal drug exposure/ or prenatal drug exposure/ or prenatal exposure/ or maternal obesity/	49141
5	perinatal period/ or prenatal period/	50832
6	perinatal care/ or maternal nutrition/ or prepregnancy care/ or prenatal care/ or fetal malnutrition/	79807
7	prenatal development/ or fetus development/ or fetal well being/ or prenatal growth/ or fetus growth/ or fetus maturity/	96043
8	"parameters concerning the fetus, newborn and pregnancy"/ or apgar score/ or exp low birth weight/ or birth weight/ or exp very low birth weight/ or crown rump length/ or fetus outcome/ or fetus risk/ or fetus weight/ or gestation period/ or gestational age/ or litter size/ or perinatal morbidity/ or placenta weight/ or *pregnancy outcome/ or first trimester pregnancy/ or mother fetus relationship/ or second trimester pregnancy/ or third trimester pregnancy/	401154
9	early life stress/ or perinatal stress/	4813
10	gestational weight gain/ or *pregnancy disorder/ or fetomaternal transfusion/ or fetus disease/ or prenatal disorder/ or prenatal injury/ or prenatal stress/ or exp chorioamnionitis/ or fetal alcohol syndrome/ or fetal malnutrition/ or fetotoxicity/ or fetus distress/ or fetus hypoxia/ or intrauterine growth retardation/ or macrosomia/ or exp multiple pregnancy/ or placenta disorder/ or placenta insufficiency/ or exp *pregnancy complication/ or *pregnancy toxemia/ or exp *"eclampsia and preeclampsia"/ or *maternal hypertension/ or exp *"immature and premature labor"/ or premature fetus membrane rupture/ or exp *pregnancy diabetes mellitus/	338968
11	exp parental age/ or parity/ or exp parental smoking/	94758
12	(head circumference/ or leg length/ or cephalometry/) and (exp infant/ or fetus/ or birth/ or birth rate/ or live birth/ or ((birth not (birth adj6 (cohort* or defect* or control or match* or adjust*))) or births* or childbirth* or born or early-life or parturit*).tw,kw.)	13807
13	birth certificate/ or birthplace/ or birth order/ or birth setting/ or birth season/ or ((exp season/ or geographic distribution/ or environment/ or rural area/ or exp urban area/ or rural population/ or suburban population/ or urban population/ or urban rural difference/ or urbanization/ or *immigrant/ or life event/ or hunger/ or exp food deprivation/) and (birth/ or birth rate/ or live birth/ or ((birth not (birth adj6 (cohort* or defect* or control or match* or adjust*))) or births* or childbirth* or born or early-life or parturit*).tw,kw.))	42927
14	spanish influenza/ or (hunger/ and (china/ or chinese/)) or ((age/ or educational status/ or education/ or academic achievement/ or socioeconomics/ or exp economic status/ or exp income group/ or poverty/ or social status/ or social class/ or exp employment status/ or coal mining/ or agricultural worker/ or coal worker/ or industrial worker/ or manual labor/ or income/ or occupation/ or occupational exposure/ or occupational hazard/ or nutritional status/ or hunger/ or nutritional deficiency/ or exp vitamin deficiency/ or malnutrition/ or exp vitamin intake/ or exp folic acid derivative/ or thiamine/ or exp pollution/ or exp heavy metal/ or endocrine disruptor/ or environmental exposure/ or environmental disease/ or pesticide/ or particulate matter/ or exp radiation exposure/ or epidemic/ or pandemic/ or endemic/ or exp congenital infection/ or exp influenza A/ or exp influenza virus/ or cytomegalovirus infection/ or	151260

	cytomegalovirus/ or exp "substance use"/) and (parent/ or mother/ or expectant mother/ or father/ or expectant father/ or uterus/ or *birth/ or childbirth/ or birth rate/ or live birth/ or ((birth adj2 (patient* or date or rate)) or followed-from-birth or before-birth* or after-birth* or ("at-birth" not (life expectan* adj2 birth*)) or ((across-life or throughout-life or ((across or follow*) adj3 life course)) and (early or birth))).tw,kw. or (f?etus* or f?etal).mp.))	
15	exp digit ratio/	284
16	(intra-uterin* or intrauterin* or utero or antenat* or ante-nat* or prenat* or pre-nat* or perinat* or peri-nat* or preconceptional* or pre-conceptional* or periconcept* or peri-concept* or postconcept* or post-concept* or pre-birth* or prebirth*).tw,kw.	426746
17	(gestational or (SGA adj3 (infant* or neonat* or newborn* or neo-nat* or new*-born* or pregnan* or gestat* or birth* or weight*))).tw,kw.	194683
18	(DOHAD* or FOAD*).tw,kw. or (development* adj2 origin* adj3 (health* or diseas*)).tw,kw,jw.	2607
19	(((f?etal or f?etus* or embryo* or pregnan* or early or developmental or neurodevelop* or nutrition*) adj9 programming) or ((f?etal or hormon*) adj3 priming)).tw,kw.	8542
20	(f?etal adj3 (period* or life or expos* or famine or nutri* or undernutr* or malnutr* or overnutrit* or obes* or stress* or origin* or start* or begin* or (root* not dorsal root*) or antecedent* or hypothes*)).tw,kw.	29911
21	(((f?etal or f?etus*) adj3 (growth or compromis* or preterm or pre-term* or prematur* or pre-matur*)) or IUGR* or FGR* or SFGR* or SIUGR* or previab* or pre-viab* or "before viab*" or (placent* adj3 (insufficien* or d*sfunct*))).tw,kw.	54091
22	((PROM not prospective memor*) or PPROM* or EPPROM*1 or ((prematur* or pre-matur* or i?matur* or preterm* or pre-term*) adj6 (ruptur* or labor or labour or labo?ring or contraction* or birth or births or baby or babies or childbirth* or delivery or deliveries or parturit* or infant* or neonat* or neo-nat* or new*born* or new*-born*)) or VPTB* or MPTB* or prelabo?r or pre-labo?r).tw,kw.	194012
23	((early adj (labor or labour or parturit* or deliver* or birth or births or childbirth*)) or ((gestat* or age) adj2 ("at birth" or "at deliver*"))).tw,kw.	18273
24	(((birth or births or baby or babies or neonat* or neo-nat* or new*born* or new*-born* or f?etal) adj3 (underweight* or weight* or size or length* or height* or BMI or body mass*)) or birthweight* or LBW* or VLBW* or ELBW*).tw,kw.	146896
25	((((head or skull*) adj2 (circumfere* or size* or small*)) or ((leg or limb*) adj2 (size* or length* or small*)) or cephalometr*) adj9 (birth or births or baby or babies or neonat* or neo-nat* or new*born* or new*-born* or postnat* or postnat* or early-life* or infant* or child*)).tw,kw.	7074
26	(interpregnan* or inter-pregnan* or ((pregnan* or birth*) adj3 interval*)).tw,kw,dq.	5649
27	(HELPP or preeclam* or eclamp*).tw,kw.	65720
28	(((maternal or mother* or gestat* or pregnanc* or pregnant or gravid* or trimester*) adj4 (expos* or radiat* or irradiat* or addict* or substance abus* or smoking or tobacco or cigarett* or nicotin* or alcohol* or caffein* or drug* or psychotrop* or narcotic* or mari*uana or hash* or cocain* or amphetamin* or amfetamin* or MDMA or opium or opiat* or heroin* or GHB or ketamin* or LSD or antidepres* or anti-depres* or SSRI* or SNRI* or (serotonin* adj3 reuptake inhibitor*) or cipramil or lexapro or prozac or fevarin* or seroxat or zoloft or cymbalta or efexor or effexor or pristiq or fetzima or ixel or savella or milnacipran or monoamine oxidase inhibitor* or MAOIs or MAO-inhibitor* or analgesic* or painkiller* or anti-inflammator* or aspirin* or ((cox* or	310981

	cyclooxygenase or cyclo-oxygenas*) adj3 (inhibitor* or block* or antagon*)) or coxib* or celecoxib or diclofenac or ibuprofen or indomethicin* or naproxen or acetaminophen* or acetylsalicylic or aspirin* or antidiabetic* or metformin* or cortico* or cortisol or hydrocort* or steroid* or glycosteroid* or glycocortico* or dexameth* or prednis* or betameth* or infect* or influenz* or virus* or viral or CMV or cytomegalovir* or toxin* or heavy-metal* or lead or Pb or mercury or Hg or arsen* or cadmium or chromium or Cr or Nickel or Ni or pollut* or chemic* or endocrine disrupt* or BPA or BPAs or bisphenol* or PFOA or PFOAS or PFTE or teflon or perfluoro* or per-fluoro* or polychlor* or PCB or PCBs or biphenyl* or phalat* or perchlorat* or plastic* or pesticid* or asbest* or solvent* or thinner* or nutrit* or undernutrit* or malnutrit* or famine or hunger or obes* or overnutrit* or supplement* or vitamin* or multivitam* or vit-D or vit-B12 or vit-B6 or vit-C or vit-A or vit-E or retinol or ascorbic* or ascorbat* or tocopherol* or alphatocopherol* or tocotrienol* or cobalamin* or pyridoxin* or folic acid or folate or iron or calcium or polyunsaturat* or poly-unsaturat* or monounsaturat* or mono-unsaturat* or MUFA or MUFAs or PUFA or PUFAs or LCPUFA* or LCP or LCPs or docosahex?eno* or DHA or eicosapent?en* or icosapent?en* or EPA or omega-3* or omega-6* or omega3* or omega6* or n3 or n6 or n-3 or n-6 or linolenic or linolenate* or alphalinolen* or gammalinolen* or GLA or DGLA or arachidon* or ARA or weight* or BMI or body mass* or diet or diets or dietary or stress or diabet* or GDM or hyperten* or blood pressur* or an?emi*)) or (maternal adj2 offspring*)).tw,kw.	
29	((birth or births) adj2 (record* or chart* or certificat* or index)).tw,kw.	8377
30	((maternal or paternal or parent* or mother* or father*) adj3 (age or ages)).tw,kw.	62650
31	((born or birth or child* or infant*) adj3 (older or old or young*) adj2 (mother* or father* or parent*)).tw,kw.	5972
32	((maternal or mother*) adj3 (parity or multipar* or nullipar* or primipar*)).tw,kw.	8260
33	(((maternal or paternal or parent* or mother* or father*) adj2 educat*) or ((maternal or paternal or parental or parents or mother* or father*) adj5 (social status or socioeconomic* or economic* or SEP or cSEP or SES or cSES or income*1 or poverty or occupat* or employ* or unemploy* or mining or miners or coal or industr*))).tw,kw.	52703
34	(((condition* or characteristic* or circumstanc* or origin* or expos* or etiol* or aetiol* or (caus* not all-caus*-death*) or factor* or environment* or social status or socioeconom* or econom* or SEP or cSEP or SES or cSES) adj9 (birth or births or childbirth*)) not ((condition* or characteristic* or circumstanc* or origin* or expos* or etiol* or aetiol* or caus* or factor* or environment* or social status or socioeconom* or econom* or SEP or cSEP or SES or cSES) adj11 (birth cohort* or birth defect* or birth control))).tw,kw.	62931
35	(((condition* or characteristic* or circumstanc* or origin* or expos* or etiol* or aetiol* or caus* or social-status or socioeconom* or econom* or SEP or SES or cSES or program* or hunger or famine or nutritional deficien* or program* or event*) adj3 early-life) or (early life adj1 (factor* or variable* or environment* or precursor* or stress or residence)) or early life risk factor* or early exposur* or early famine or ((chinese or world war) adj3 famine) or spanish flu or 1918-influenza*).tw,kw.	17734
36	(((season* or winter* or summer* or autumn* or spring or springtime or month or quarter*) adj4 (birth or births or birthrate* or childbirth* or born)) or "time-of-birth*").tw,kw.	13282
37	(birth year not (match* adj6 birth year)).tw,kw.	2207
38	(((later or earlier or order) adj2 (birth or births or childbirth* or borns or born or sibling* or sibship*)) or ((first or 1st or second* or 2nd or third or 3rd or fourth or 4th) adj (born* or sibling*)) or ((number or size) adj2 (sibship* or	12095

	sibling*)) or firstborn* or (each-additional adj2 (child or infant* or born))).tw,kw. not (twin or twins).mp.	
39	(((state or states or country or countries or county or counties or place or residenc* or region* or ((area or areas) not (surface adj3 area*)) or location* or overseas or foreign* or nativ* or rural or urban or cities or suburban or residential or industrial or mine or mines or coalmine*) adj3 ((birth not (birth adj6 (cohort* or defect* or control or match* or adjust*))) or births or childbirth* or born)) or (birthplace* not ((adjust* or match*) adj6 birthplac*))).tw,kw.	22980
40	(((second-to-fourth or index-to-ring) adj4 (finger* or digit*)) or ((digit or finger length*) adj3 (ratio* or 2d-4d or 2d?4d)) or ((2d-4d or 2d?4d or index finger* or ring finger*) adj4 (ratio or ratios or length* or male* or female* or gender))).tw,kw.	1664
41	((f?etal or fetus*) adj3 (hormon* or testosteron or androgen* or ?estrogen* or cortisol* or corticoster*)).tw,kw.	4397
**42**	**or/4-41 [prenatal factors/exposure]**	1406392
**43**	**3 and 42 [AD - prenatal exposure/factors]**	3538
44	((exp animal/ or nonhuman/) not human/) or (animal* or veterinar*).jw. or animal experiment/ or exp animal model/ or exp experimental animal/ or exp female animal/ or exp domestic cattle/ or exp rodent/ or (((primates or ape or apes or monkey* or baboon* or macaq* or pig or pigs or porcine or goat or goats* or sheep or lamb or lambs or ovine or cattle or bovine or cow or cows or horse or horses or mare or calve or calves or dog or dogs or canine or bitch* or cat or cats or feline or rodent* or rabbit* or mice or mouse or murine* or rat or rats or frog or frogs or zebra* or drosophila* or chick* or bee or bees).ti,ot. or (dam or dams or pups or pup or ewe or ewes or sow or sows or mice or mouse or murine or rat or rats or wistar or sprague or transgenic or Tx* or C57BL* or Balb-c or Balbc or wistar or sprague or dawley).tw,kw.) not (human* or patient* or infants or neonates or women or men).ti.)	9423627
45	(down adj2 (syndrom* or model*)).ti,ot.	13780
**46**	**44 or 45 [exclusion animals not humans, and down syndrome in title]**	9435971
**47**	**43 not 46 [human studies on AD/prenatal factors not Down syndrome]**	2649
48	editorial/ or "systematic review"/ or practice guideline/ or consensus development/ or (editorial or conference abstract or conference review or note).pt. or (committee or editorial or reply or guideline* or systematic or (case-report not case-report-survey) or two-cases).ti. or cochrane.jw. or ((review.pt. or review/ or case report/ or (review or overview).ti. or (search* adj12 (literature* or ((electronic or medical or biomedical) adj3 database*) or exhaustive or systematic or medline or pubmed or embase or psychinfo or (CENTRAL and cochrane) or "Central Register of Controlled Trials")).tw,kw.) not (exp medical record/ or cohort analysis/ or longitudinal study/ or prospective study/ or retrospective study/ or exp case control study/ or cross-sectional study/ or (case-control* or cohort* or retrospective or prospectiv* or crosssection* or cross-section* or population-based).ti,ot. or ((chart* or record* or retrospectiv*) adj3 review*).tw,kw.)) [Filter for original studies]	12564024
**49**	**47 not 48 [original human studies on AD + prenatal factors]**	1144
**50**	**remove duplicates from 49 [original human studies on AD + prenatal factors - dedub]**	1114
**51**	**50 not medline.cr. [human AD + prenatal factors - deduplicated - embase records only]**	943
