# Supplementary material for: Do prenatal factors shape the risk for dementia?: A systematic review of the epidemiological evidence for the prenatal origins of dementia
Source: Soc Psychiatry Psychiatr Epidemiol. 2023 Apr 8;60(5):977–91. doi: 10.1007/s00127-023-02471-7 (PMC12119685; doi:10.1007/s00127-023-02471-7)
Supplement: Supplementary file 3 — Supplementary file3 (PDF 659 KB) [file 127_2023_2471_MOESM3_ESM.pdf]

Online Resource 3: Tables including the risk of bias results

Social Psychiatry and Psychiatric Epidemiology

Do prenatal factors shape the risk for dementia?: A systematic review of the epidemiological evidence for the prenatal origins of dementia

Aline Marileen Wiegersma*, Amber Boots, Miranda W. Langendam, Jacqueline Limpens, Susan D. Shenkin, Aniko Korosi, Tessa J. Roseboom, Susanne R. de Rooij

*Corresponding author: Amsterdam UMC location University of Amsterdam, Epidemiology and Data Science, Meibergdreef 9, Amsterdam, The Netherlands, e-mail: a.m.wiegersma@amsterdamumc.nl

Parental age and birth order

Case-control studies

Author	Ref.	Selection				Comparability	Exposure			
		Is the case definition adequate?	Representativeness of the cases	Selection of controls	Definition of controls	Comparability of cases and controls on the basis of the design or analysis[a]	Ascertainment of exposure	Same method of ascertainment for cases and controls[b]	Non-Response rate	Overall risk of bias
Max		*	*	*	*	**	*	*	*	
**Parental age and birth order**										
Amaducci	60	*	*	*	*	**		*		Low
Bertram	45	*		*	*	**				Intermediate/high
Chandra	63	*	*		*	**	*	*		Low
Clarnette	46	*	*	*	*			*		Intermediate/high
Cohen	43	*	*	*	*					Intermediate/high
Corkin	42	*		*	*			[c]		Intermediate/high
De Braekeleer 1988	44	*	*	*	*	**	*	*		Low
English	47	*		*	*				*	Intermediate/high
Farrer 1991	33	*	*	*	*	**				Intermediate/high
Farrer 1997	59	*	*	*	*	**				Intermediate/high
Forster	48	*	*	*	*	**		*		Low
Fratiglioni	61	*		*	*	**		*		Intermediate/high
Graves	49	*		*	*	**		*		Intermediate/high
Heyman	41	*	*	*	*	*				Intermediate/high
Hofman	50	*	*	*	*	**		*		Low
Jouan-flahault	54	*		*	*	**	*	*	*	Low
Knesevich	51	*		*	*	*	*	*	*	Low
Li	64	*	*	*	*	**		*		Low

Lindsay	40	*	*	*	*	**		*	*	Low
Moceri – 2000	52	*		*	*	**		*		Intermediate/high
Moceri – 2001	35	*		*	*	**	*	*		Low
Prince	36	*	*	*	*	**		*	*	Low
Ptok 2000	37	*	*	*	*	**		*		Low
Tsolaki	38	*		*	*			*		Intermediate/high
Urakami	53	*	*	*	*		*	*		Intermediate/high
Whalley 1982	62	*			*	**	*	*		Intermediate/high
Whalley 1995	34	*	*		*	**	*	*		Low
White	39	*	*	*	*	*				Intermediate/high

[a] *The study accounted for age/year of birth in the design or analysis, *the study accounted for any other factor in the design or analysis.

[b]We interpreted this question as the risk of misclassification is similar between cases and controls.

[c]Technically used the same method, however bias is likely.

We defined ≥7 stars as low risk of bias and <7 starts as intermediate or high risk of bias.

Cohort studies

Author	Ref.	Selection				Comparability	Outcome			
		Representativeness of the exposed cohort	Selection of the non-exposed cohort	Ascertainment of exposure	Demonstration that outcome of interest was not present at start of study	Comparability of cohorts on the basis of the design or analysis[a]	Assessment of outcome	Was follow-up long enough for outcomes to occur	Adequacy of follow up of cohorts	Overall risk of bias
Max		*	*	*	*	**	*	*	*	
**Parental age and birth order**										
Katzman	55		*	*	*		*	*	*	Intermediate/high
Lahti	67	*	*	*	*	**	*		*	Low
Mosing	65	*	*	*	*	*	*	*	*	Low
Tyas	56	*	*		*	**	*	*	*	Low
Vaillant	57		*		*	**	*	*	*	Low

[a] *The study accounted for age/year of birth in the design or analysis, *the study accounted for any other factor in the design or analysis.

We defined ≥7 stars as low risk of bias and <7 starts as intermediate or high risk of bias.

Retrospective cohort/cross-sectional

Author	Ref.	Selection				Comparability	Outcome		
		Representativeness of the exposed cohort	Sample size	Ascertainment of exposure	Non-respondents	The subjects in different outcome groups are comparable, based on the study design or analysis[a]	Assessment of outcome	Statistical test	Overall risk of bias
Max		*	*	*	*	**	*	*	
**Parental age and birth order**									
Kim	66	*	*	*			*	*	Intermediate/high
Reisz	58	*				**	*	*	Intermediate/high

[a] *The study accounted for age/year of birth in the design or analysis, *the study accounted for any other factor in the design or analysis.

We defined ≥6 stars as low risk of bias and <6 starts as intermediate or high risk of bias.

Season of birth

Case-control studies

Author	Ref.	Selection				Comparability	Exposure			
		Is the case definition adequate?	Representativeness of the cases	Selection of controls	Definition of controls	Comparability of cases and controls on the basis of the design or analysis[a]	Ascertainment of exposure	Same method of ascertainment for cases and controls	Non-Response rate	Overall risk of bias
Max		*	*	*	*	**	*	*	*	
**Season of birth**										
Dysken	68	*		*	*	*	*			Intermediate/high
Fratiglioni	61	*		*	*	**		*		Intermediate/high
Frazee	74	*	*	*	*	*	*			Intermediate/high
Henderson	69	*		*	*	**		*		Intermediate/high
Koch	75	*		*	*	*	*			Intermediate/high
Lawlor	72	*		*	*	*				Intermediate/high
Philpot	73	*		*	*					Intermediate/high
Prince	36	*	*	*	*	**		*	*	Low
Ptok 2001	70	*	*	*	*	**		*		Low
Tolppanen	76	*	*	*	*	**	*	*	*	Low
Vezina	80	*		*	*	*				Intermediate/high
Vitiello	71	*	*	*	*					Intermediate/high

[a] *The study accounted for age/year of birth in the design or analysis, *the study accounted for any other factor in the design or analysis.

We defined ≥7 stars as low risk of bias and <7 starts as intermediate or high risk of bias.

Cohort studies

Author	Ref.	Selection				Comparability	Outcome			
		Representativeness of the exposed cohort	Selection of the non-exposed cohort	Ascertainment of exposure	Demonstration that outcome of interest was not present at start of study	Comparability of cohorts on the basis of the design or analysis[a]	Assessment of outcome	Was follow-up long enough for outcomes to occur	Adequacy of follow up of cohorts	Overall risk of bias
Max		*	*	*	*	**	*	*	*	
**Season of birth**										
Doblhammer	77	*	*	*		**	*			Intermediate/high
Hsu	81	*	*	*	*	*	*	*	*	Low
Mooldijk	79	*	*	*	*	**	*	*	*	Low

[a] *The study accounted for age/year of birth in the design or analysis, *the study accounted for any other factor in the design or analysis.

We defined ≥7 stars as low risk of bias and <7 starts as intermediate or high risk of bias.

Retrospective cohort/cross-sectional

Author	Ref.	Selection				Comparability	Outcome		
		Representativeness of the exposed cohort	Sample size	Ascertainment of exposure	Non-respondents	The subjects in different outcome groups are comparable, based on the study design or analysis[a]	Assessment of outcome	Statistical test	Overall risk of bias
Max		*	*	*	*	**	*	*	
**Season of birth**									
Ding	78	*	*	*	*	**	*	*	Low

[a] *The study accounted for age/year of birth in the design or analysis, *the study accounted for any other factor in the design or analysis.

We defined ≥6 stars as low risk of bias and <6 starts as intermediate or high risk of bias.

Place of birth

Case-control studies

Author	Ref.	Selection				Comparability	Exposure			
		Is the case definition adequate?	Representativeness of the cases	Selection of controls	Definition of controls	Comparability of cases and controls on the basis of the design or analysis[a]	Ascertainment of exposure	Same method of ascertainment for cases and controls	Non-Response rate	Overall risk of bias
Max		*	*	*	*	**	*	*	*	
**Place of birth**										
Baker	83	*	*		*	*		*		Intermediate/high
Emard	90	*			*			*		Intermediate/high
Forster	48	*	*	*	*	**		*		Low
Frecker	89	*	*	*	*	*	*	*	*	Low
Jean	82	*		*	*	*				Intermediate/high
Prince	36	*	*	*	*	**		*	*	Low

[a] *The study accounted for age/year of birth in the design or analysis, *the study accounted for any other factor in the design or analysis.

We defined ≥7 stars as low risk of bias and <7 starts as intermediate or high risk of bias.

Cohort studies

Author	Ref.	Selection				Comparability	Outcome			
		Representativeness of the exposed cohort	Selection of the non-exposed cohort	Ascertainment of exposure	Demonstration that outcome of interest was not present at start of study	Comparability of cohorts on the basis of the design or analysis[a]	Assessment of outcome	Was follow-up long enough for outcomes to occur	Adequacy of follow up of cohorts	Overall risk of bias
Max		*	*	*	*	**	*	*	*	
**Place of birth**										
Gilsanz 2017	85	*	*	*	*	**	*	*	*	Low
Gilsanz 2019	88	*	*	*	*	**	*	*	*	Low
Topping	87	*	*		*	**	*	*	*	Low
Wilson	91		*	*	*	**	*	*	*	Low

[a] *The study accounted for age/year of birth in the design or analysis, *the study accounted for any other factor in the design or analysis.

We defined ≥7 stars as low risk of bias and <7 starts as intermediate or high risk of bias.

Retrospective cohort/cross-sectional

Author	Ref.	Selection				Comparability	Outcome		
		Representativeness of the exposed cohort	Sample size	Ascertainment of exposure	Non-respondents	The subjects in different outcome groups are comparable, based on the study design or analysis[a]	Assessment of outcome	Statistical test	Overall risk of bias
Max		*	*	*	*	**	*	*	
**Place of birth**									
Glymour	86	*	*	*		**	*	*	Low
Guaita	92	*	*	*	*	**	*	*	Low
Scazufca	84	*	*	*		**	*	*	Low

[a] *The study accounted for age/year of birth in the design or analysis, *the study accounted for any other factor in the design or analysis.

We defined ≥6 stars as low risk of bias and <6 starts as intermediate or high risk of bias.

Other factors

Case-control

Author	Ref.	Selection				Comparability	Exposure			
		Is the case definition adequate?	Representativeness of the cases	Selection of controls	Definition of controls	Comparability of cases and controls on the basis of the design or analysis[a]	Ascertainment of exposure	Same method of ascertainment for cases and controls	Non-Response rate	Overall risk of bias
Max		*	*	*	*	**	*	*	*	
**Other factors**										
Jiang	97	*		*	*	*	*	*		Intermediate/high
Vladeanu	96	*		*	*	**		*		Intermediate/high

[a] *The study accounted for age/year of birth in the design or analysis, *the study accounted for any other factor in the design or analysis.

We defined ≥7 stars as low risk of bias and <7 starts as intermediate or high risk of bias.

Cohort

Author	Ref.	Selection				Comparability	Outcome			
		Representativeness of the exposed cohort	Selection of the non-exposed cohort	Ascertainment of exposure	Demonstration that outcome of interest was not present at start of study	Comparability of cohorts on the basis of the design or analysis[a]	Assessment of outcome	Was follow-up long enough for outcomes to occur	Adequacy of follow up of cohorts	Overall risk of bias
Max		*	*	*	*	**	*	*	*	
**Other factors**										
Cocoros	93	*	*	*	*	**	*	*	*	Low
Kang	94	*	*	*	*		*			Intermediate/high
Luo	98	*	*	*	*	**	*	*	*	Low

[a] *The study accounted for age/year of birth in the design or analysis, *the study accounted for any other factor in the design or analysis.

We defined ≥7 stars as low risk of bias and <7 starts as intermediate or high risk of bias.

Retrospective cohort/cross-sectional

Author	Ref.	Selection				Comparability	Outcome		
		Representativeness of the exposed cohort	Sample size	Ascertainment of exposure	Non-respondents	The subjects in different outcome groups are comparable, based on the study design or analysis[a]	Assessment of outcome	Statistical test	Overall risk of bias
Max		*	*	*	*	**	*	*	
**Other factors**									
Lenz	95	*				*	*	*	Intermediate/high

[a] *The study accounted for age/year of birth in the design or analysis, *the study accounted for any other factor in the design or analysis.

We defined ≥6 stars as low risk of bias and <6 starts as intermediate or high risk of bias.

Birth characteristics

Cohort studies

Author	Ref.	Selection				Comparability	Outcome			
		Representativeness of the exposed cohort	Selection of the non-exposed cohort	Ascertainment of exposure	Demonstration that outcome of interest was not present at start of study	Comparability of cohorts on the basis of the design or analysis[a]	Assessment of outcome	Was follow-up long enough for outcomes to occur	Adequacy of follow up of cohorts	Overall risk of bias
Max		*	*	*	*	**	*	*	*	
**Birth characteristics**										
Mosing	99	*	*	*	*	*	*	*	*	Low
Matshusima	65	*	*			**		*		Intermediate/high
Sydall	100	*	*	*	*	**	*	*	*	Low

[a] *The study accounted for age/year of birth in the design or analysis, *the study accounted for any other factor in the design or analysis.

We defined ≥7 stars as low risk of bias and <7 starts as intermediate or high risk of bias.
