# Supplementary material for: Do prenatal factors shape the risk for dementia?: A systematic review of the epidemiological evidence for the prenatal origins of dementia
Source: Soc Psychiatry Psychiatr Epidemiol. 2023 Apr 8;60(5):977–91. doi: 10.1007/s00127-023-02471-7 (PMC12119685; doi:10.1007/s00127-023-02471-7)
Supplement: Supplementary file 4 — Supplementary file4 (PDF 858 KB) [file 127_2023_2471_MOESM4_ESM.pdf]

Online Resource 4: Tables including the data extraction results

Social Psychiatry and Psychiatric Epidemiology

Do prenatal factors shape the risk for dementia?: A systematic review of the epidemiological evidence for the prenatal origins of dementia

Aline Marileen Wiegersma*, Amber Boots, Miranda W. Langendam, Jacqueline Limpens, Susan D. Shenkin, Aniko Korosi, Tessa J. Roseboom, Susanne R. de Rooij

*Corresponding author: Amsterdam UMC location University of Amsterdam, Epidemiology and Data Science, Meibergdreef 9, Amsterdam, The Netherlands, e-mail: a.m.wiegersma@amsterdamumc.nl

Content

Table A – Basic information (parental age and birth order, season of birth, place of birth, other factors, birth characteristics)

Table B – Information and Results (parental age and birth order, season of birth, place of birth, other factors, birth characteristics)

Table C – Subgroup results (parental age and birth order, season of birth, place of birth, other factors, birth characteristics)

Table A – Basic information

Parental age and birth order

Author	Ref.	Publication year	N[a]	Men/women	Location	Population	Exposure	Exposure ascertainment	Comparison/control
**Parental age and birth order**									
Case-control studies									
Amaducci	60	1986	329	82/150 (excluding population controls)	Italy; seven centers throughout the country	Patients from neurology departments, hospital and population control groups	Maternal age (>40)<br>Paternal age (>44)<br>Birth order (≥2)<br>Birth order (≥4)	Next-of-kin interview (for cases and controls)	Community controls and hospital controls[a]
Bertram	45	1998	154	Unclear	Germany	Patients from outpatient clinic, Caucasian	Maternal age<br>Paternal age	Family history questionnaire completed by proxy; not clear if a proxy was also used for the controls	Age and sex matched volunteers; no family history of dementia; scored ≥ 28 on MMSE
Chandra	63	1987	128	30/98	Denver, Colorado, USA	Participants from outpatient clinic for senior citizen's	Birth order (≥4)	Surrogate/next of kin respondent (for cases and controls)	Patients without dementia from the same outpatient clinic, affected by one or more medical conditions
Clarnette	46	1992	Maternal age: 332; paternal age: 318	For cases (including 56 none AD cases) 98/116	Hamilton, Canada	Referrals to a memory clinic and their spouses	Maternal age<br>Paternal age	From subjects and/or relatives; often verified from family documents	Spouses
Cohen	43	1982	670 (80 cases)	For cases 50/30	Washington state, USA	Patients form community AD registry, Caucasians born between 1891 and 1921; controls from selected birth records	Maternal age	Cases: Questionnaire sent to family members; when possible (half of the cases) also through family records and birth certificates<br>Controls: via birth records	Randomly selected birth records for persons born in 1907 in Washington state in 1907
Corkin	42	1983	71	34/37	Massachusetts, USA	Participants form research studies on aging and dementia	Maternal age<br>Paternal age	Interview with participants. If necessary obtained from or verified by relatives.	Spouses without dementia (12/34) and volunteers (12/34) of similar age and SES
De Braekeleer	44	1988	133 cases		Saguenay-Lac-St-Jean (SLSJ) region, isolated	Inhabitants of the SLSJ region	Maternal age<br>Paternal age<br>Birth order	Population registry	Comparison with siblings, spouses and six control groups (parental age); Siblings (birth order)

					region, Canada				
English	47	1985	163	Not reported	Washington state, USA	Cases: from a hospital and from a self-help organization for relatives of persons with AD<br>Controls: spouses from AD and Parkinson patients.	Maternal age	Questionnaire filled in by participant (controls) or by relative (cases)	Spouses of the AD patients and spouses form Parkinson patients
Farrer	33	1991	1422	570/852	Massachusetts, USA	Cases: from hospital<br>Controls: from Framingham study	Maternal age<br>Paternal age	Cases: informant interview<br>Controls: collected from Framingham study records	Participants of the Framingham study
Farrer	59	1997	2091	928/1163	USA	Cases: form different hospitals in the MIRAGE project<br>Controls: from Framingham study	Maternal age<br>Paternal age	Cases: from family history questionnaire by informant; verified by other informants and available medical records<br>Controls: from parental death certificates	Participants of the Framingham study
Forster	48	1995	218	96/122	Northern health region of England	Cases: patients referred to specialist hospital services, who had been diagnosed as having dementia before the age of 65 years during the period 1981-89<br>Controls: randomly selected from the general population	Maternal age	All data concerning history of exposure in both cases and controls were obtained from informants	Controls were randomly selected from the population of the northern health region by a two stage procedure. Firstly, for each case, a family health service authority (FHSA) was chosen randomly. The authority then supplied the names of five individuals of the same sex and age as the case. If needed a replacement control from the potential list of five was selected randomly. The possibility of dementia in the control was excluded.
Fratiglioni	61	1993	364	67/297	Stockholm, Sweden	Inhabitants from an area in Stockholm that participated in the Kungsholmen project and underwent the MMSE	Maternal age<br>Paternal age<br>Birth order	Structured interview with informant (close relative or other significant person)	Subjects who had MMSE ≥ 34 and were not diagnosed as affected by dementia in the Kungsholmen project
Graves	49	1990	260; 188 and 162 in maternal /paternal age analyses	140/120	Seattle area, Washington state, USA	Patients from two clinics; cases and controls were all married for ≥ 10 years	Maternal age<br>Paternal age	Structured telephone interview with surrogate	Friend of the patient or a patient surrogate; else a relative not related by blood

Heyman	41	1983	72	Not reported	1/2 North Carolina, 1/3 other southeastern states, else from northeastern/midwestern states, USA	Consecutive series of AD patients that had participated in a comprehensive clinical, genetic and epidemiological study of AD at Duke University Medical Center and their spouses	Maternal age<br>Paternal age	Determined with interview. For cases interview with informant (usually the spouse)	Close relative, usually the spouse
Hofman	50	1990	368	Not reported	Four northern provinces and Rotterdam area, Netherlands	Part of a case-control study of AD, Diagnosis <70	Maternal age<br>Paternal age<br>Birth order	Structured interview with next of kin (mostly from wife, husband or a child); Information was checked by either the municipal population register or information from a second family member	Randomly selected from the municipal population register; SPMSQ score ≥ 20 and apparently healthy
Jouan-flahault	54	1989	180	Not reported	Haute-Normandie, France	Elderly population in Haute-Normandie, living at home or in an institution (random sample)	Maternal age<br>Paternal age	Birth records and registry data from birth municipality	Two matched controls per case; from the same population as cases but without an indication for dementia
Knesevich	51	1982	94	Not reported	Washington, USA	From memory and aging project at the Washington University Medical Center; Caucasian	Maternal age	Obtained from vital statistics records (official government records), or else records from funeral parlors, monument companies, cemeteries and crematories, cross-checked against family documents	Healthy individuals who had good intellectual function in relation to past performance
							Birth order	No description	Comparison with the general US population form vital statistic rates in the US 1900-1940
Li	64	1992	210	99/111	Beijing, people's republic of China	Cases: outpatients and inpatients mostly from two psychiatric hospitals and neurologic clinics in two general hospitals	Birth order (≥4)<br>Birth order (≥6)	Structured and standardized interview with surrogate informants	Controls from neighborhoods of the patients. MMSE > 20
Lindsay	40	1994	793	Not reported	Canada (all provinces)	Cases and controls from community and institutions, drawn from lists of community residents in 36 cities and surrounding areas based on health insurance plans (universal coverage); In Ontario, the Enumeration Composite Record was used	Maternal age<br>Paternal age	Interview by informant for cases and controls	Selected from community and institution (like cases), with normal cognitive functioning according to test
Moceri	52	2000	770	282/488	Seattle, Washington, USA	Cases: from the University of Washington/GHC AD Patient registry (ADPR).	Maternal age<br>Birth order	Structured interview by research nurses with informants	Selected at random from the health maintenance organization; did not have dementia or other neurologic disease causing dementia; MMSE score ≥28.

						Controls: from a health-maintenance organization. Representative for population in area, although slightly higher educated			
Moceri	35	2001	484	184/300	Seattle, Washington, USA	Cases: from the University of Washington/GHC AD Patient registry (ADPR)<br>Controls: from a health-maintenance organization; representative for population in area, although slightly higher educated	Maternal age<br>Paternal age<br>Birth order	From birth certificate or calculated from ages listed on the U.S. census; if they differed birth certificate was used	Selected at random from the health maintenance organization; did not have dementia or other neurologic disease causing dementia
Prince	36	1994	273	Not reported	England, Scotland and Wales	Participants form primary care practices participating in the MRC hypertension trial	Maternal age (>34)<br>Paternal age (>37)	Risk factor questionnaires by MRC research nurses to the subject and an informant for each participant	Unimpaired, unmatched control from same population with intact PALT scores
Ptok	37	2000	238	85/153	Bonn, Germany	Consecutive patients recruited form hospital admissions, controls randomly chosen from general population	Maternal age<br>Paternal age	Interview with patients, controls and their relatives; only used if there was consistency among relatives and no doubt about accuracy	Healthy control subjects from the general population, chosen with city census agencies
Tsolaki	38	1997	134	62/72	Pilea, Thessaloniki, Greece	Greek residents examined under WHO Program of Aging in 1992; cases were outpatients from Neurology department in Thessaloniki	Maternal age<br>Paternal age	Interview by trained nurses with informant (a close relative, a distant relative, a friend, neighbor or another significant person	Community controls, MMSE>24, no dementia; similar in age and gender with the cases
Urakami	53	1989	220	92/128	Daisen and Kishimoto, Tottori Prefecture and Ama, Shimane Prefecture, Western Japan	Community-based study; participants involved in agriculture, industry and commerce; all fathers were wedded husbands	Maternal age<br>Paternal age	Birth records	Controls subject from persons with about the same age and with a similar socioeconomic status who did not have dementia and who were from the same areas
Whalley	62	1982	276	Cases 20/54 (5 excluded in parental age analysis)	Edinburgh, Scotland	Subjects from records in the Neuropathology Department, admission to mental hospital before age 65	Maternal age<br>Paternal age<br>Birth order	From Scottish public registry, calculated from marriage certificates	Entries in the same local register*
Whalley	34	1995	2230*	1035/1185*	Scotland	Records of Scottish Mental Hospitals	Maternal age<br>Paternal age	From marriage certificates or else from their birth or death certificates	Control selection was not reported

White	39	1986	405		Minnesota, USA	Cases died in six of the seven Minnesota state hospitals and the only state nursing home; siblings and controls selected from the same time period as the cases	Maternal age<br>Paternal age<br>Birth order	Cases: interview of a relative (usually more than 1), if possible information was checked by medical birth and death records; not reported for the siblings and controls	Normal siblings of the AD cases and individuals born during the same time period as the cases selected from birth records in Anoka County
Cohort studies									
Katzman	55	1989	434	154/280	Bronx, New York, USA	Subjects were volunteers recruited from senior citizen centers, by local newspaper advertisements, and by word of mouth, aged 75-85 at intake; the majority (70%) were Jewish; the cohort was predominantly (90%) white	Maternal age	Not specified; probably during interview at first assessment.	-
Kim	66	2007	916	376/540	Kwangju, Sourth Korea	Inhabitants age 65 or over, recorded in national residents registration lists within two defined geographic areas (one urban one rural); low-income population.	Birth order	Home-based interviews carried out by research nurses, information was collected from participants and family members	-
Lahti	67	2014	13243	6905/6338	Helsinki, Finland	Helsinki Birth Cohort Study, singleton live births at the two public maternity hospitals in Helsinki, between 1934 and 1944	Maternal grand multiparity (born as sixth or later born child)	Hospital birth records, grand multiparity defined as being born as sixth or later born child	Born as fifth child or earlier
Mosing	65	2018	35191	16324-16813/ 17842-18378	Sweden	All twins, from the Swedish Twin Registry, with information on birth characteristic, born between 1926-1960; excluding early onset dementia cases (<55 yeas)	Birth order (within twins)	Based on nationwide collection of information from original birth records, recorded by midwives and/or doctors at the time of birth	-
Reisz	58	2021	289	0/289	Kansas City, Missouri, USA	Caucasian female patients from a community-based out-patient setting in a metropolitan area of the Midwest.	Maternal age<br>Birth order<br>Age difference with the next older sibling	Exposure variables were collected in a survey	-
Tyas	56	2001	694	261/433	Manitoba, Canada	Random sampling from the provincial insurance plan, all cognitively healthy at start of the study	Maternal age<br>Paternal age	Risk factor questionnaire (1991/92)	-
Vaillant	57	2014	196	196/0	Harvard University, USA	Caucasian sophomore males at Harvard university (born circa 1920), 2/3 of men obtained graduate degrees and most have worked as physicians, lawyers, university professors or business executives	Maternal age	Not clearly reported. Likely from interview (with parents)	-

Basic information extracted from studies regarding parental age and/or birth order.

[a]This number represents the participants included in the analyses relevant for our systematic review or, if this specific number was not available, the total number of participants in the study. If the study did not include a control group but used a reference population as a comparison, or if the number of controls was not available, only the number of cases was reported.

*Reporting was not complete or not clear.

AD=Alzheimer's disease

MMSE=Mini-Mental State Examination

Season of birth

Author	Ref.	Publication year	N[a]	Men/women	Location	Population	Exposure	Exposure ascertainment	Comparison/control
**Season of birth**									
Case-control studies									
Dysken	68	1991	727 cases	Not reported	St Paul, Cleveland, Chicago, USA	Cases: from three research departments in a medical center and two universities, born between 1881 and 1940	Season of birth	Based on birth dates (likely from medical records)	Average expected quarterly birth rates for the general US population (1915-1940)
Fratiglioni	61	1993	364	67/297	Stockholm, Sweden	Inhabitants from an area in Stockholm that participated in the Kungsholmen project and underwent the MMSE	Season of birth	Swedish registry data	Subjects who had MMSE ≥ 34 and were not diagnosed as affected by dementia in the Kungsholmen project
Frazee	74	2004	1359 cases	Not reported	California, USA	Cases: from AD database, birthdates from 1906-1923.Controls form population registry	Season and month of birth	From national registry	The monthly distribution of general population birth sin California from 1906-1923
Henderson	69	1991	340	Not reported	Sydney, Australia	Cases: from two Sydney hospitals	Season of birth	Not clearly reported; based on birthdate	Controls were recruited from the same or a neighboring general practice as each case and had a MMSE ≥26; Also comparison with large population group (n=37534), from the Australian Electoral Roll, for the city of Canberra and its surrounding district of Eden-Monaro
Koch	75	2005	2917	692/2225	Regensburg, Germany	Patients treated in the psychogeriatric ward of the University clinic of Regensburg between 1995 and 2004	Season of birth	Not reported; likely based on birthdate from patient records	The data was normalized utilizing the overall births per month in Germany (Federal Statistical Office, Wiesbaden, Germany)
Lawlor	72	1993	306	Not reported	Not reported; Authors from Ireland	Not reported	Season of birth (first quarter births)	Nor reported; based on birthdate	91 age-matched healthy control subjects with no family history of dementia; the number of births per month from an age-matched, census-derived population was also available for comparison
Philpot	73	1989	239 cases	Not reported	South-East of England	Cases were consecutive referrals to two hospitals or patients enrolled in the natural history of AD	Season of birth	Not reported; for cases likely from medical records, controls were from the 1971 census sample	Controls were form the 1971 census sample

Prince	36	1994	273	Not reported	England, Scotland and Wales	Participants form primary care practices participating in the MRC hypertension trial.	Season of birth	Risk factor questionnaires by MRC research nurses to the subject and an informant for each participant	Unimpaired, unmatched controls from the same population with intact PALT scores
Ptok	70	2001	238	85/153	Bonn, Germany	Cases were recruited consecutively from hospital admissions, at least 60 years of age	Season of birth	Not clearly reported; based on birthdate*	Chosen from the general population with the support of the city census
Tolppanen	76	2016	353581	For cases: 24602/46117	Finland	The Medication and Alzheimer's Disease cohort: community-dwelling persons who received a clinically verified diagnosis of AD in 2005 to 2011	Month and season of birth	Based on registry data	One to four matched with controls, who were identified from the register including all residents of Finland who are entitled to benefits by the Social Insurance Institution (i.e. all citizens and residents living in Finland for at least two years)
Vezina	80	1996	399 cases	For cases: 124/275	Saguenay–Lac-Saint-Jean (SLSJ), Quebec, Canada	From the Image project in the SLSJ region; all cases were compared to control group 1 and cases born in the SLSJ region were compared to control group 2	Month of birth	Not clearly reported; based on birthdate, for controls from databases	Two control groups:<br>1) data obtained from the ministry of health and social services of Quebec. Born between 1893 and 1934 and living in the SLSJ area as of November 1992 or living in that area at the time of their death (1987-1992). Régie de l'assurance-maladie du Quebec, (RAMQ); N=51170).<br><br>2) All births which took place between 1893 and 1934 in SLSJ, computerized register of all the catholic parish record of baptism, marriages and sepultures that took place in the SLSJ (SOREP database; N=135142).
Vitiello	71	1991	282	130/152	Rockville Pike, Bethesda, USA	Cases: patients form the unit of Geriatric Psychopharmacology diagnosed with Alzheimer's disease, referred by secondary care providers	Season of birth	Not clearly reported; based on birthdate	Recruited through the National Institutes of Health volunteer office and advertisements in local newspapers; no evidence of dementia in functioning in the community and performance on neuropsychological screening tests and no history of major psychiatric disorders
Cohort studies									
Ding	78	2019	354859	172012/182847<br><br>Cases 496/787	China	Chinese population, non-institutionalized, representative of total Chinese population	Season of birth	Second China National Sample Survey on Disability in 2006	-

Doblhammer	77	2015	149225	Not reported	Germany	2% of all insured persons from the largest insurer in Germany (1/3 of German population). Drawn in the first quarter of 2007; SES lower than general population, bias greater in younger than older people	Season and month of birth	By date of birth in insurer's register	Summer born individuals were the reference
Hsu	81	2021	29074024	Not reported<br><br>Cases 20480/25636	Taiwan	Taiwanese national health insurance database, covering 99% of Taiwan's population. Born between 1 January 1900 and 31 December 2013	Month of birth	By date of birth in insurer's register	-
Mooldijk	79	2021	12964	5478/7486	Rotterdam, the Netherlands	Participants from the Rotterdam study aged 45 and over. Participants did not have dementia at baseline	Season of birth and severity of winter	Based on date of birth	Summer born individuals were the reference.

Basic information extracted from studies regarding season of birth.

[a]This number represents the participants included in the analyses relevant for our systematic review or, if this specific number was not available, the total number of participants in the study. If the study did not include a control group but used a reference population as a comparison, or if the number of controls was not available, only the number of cases was reported.

*Reporting was not complete or not clear.

AD=Alzheimer's disease

MMSE=Mini-Mental State Examination

Place of birth

Author	Ref.	Publication year	N[a]	Men/women	Location	Population	Exposure	Exposure ascertainment	Comparison/control
**Place of birth**									
Case-control studies									
Baker	83	1993	72	22/50	New York city, USA	Cases and controls from Burke rehabilitation center; all individuals with cognitive impairment in the past 20 years were evaluated for AD; middle-class community	Place of birth (rural vs urban?)*	Surrogate interview with relative (for cases and controls)	In some controls cognitive testing was done (not all), controls were excluded if they had dementia; controls primarily had respiratory and cardiovascular disease.
Emard	90	1994	129 cases only	Not reported	Saguenay-Lac-Saint-Jean (SLSJ), Quebec, Canada	IMAGE project	Place of birth (soil composition)	A questionnaire was sent to families of cases	No control group; comparison of birth residence soil composition to average municipality composition
Forster	48	1995	160	Not reported	Northern health region of England	Cases: patients referred to specialist hospital services, who had been diagnosed as having dementia before the age of 65 years during the period 1981-89<br>Controls: randomly selected from the general population	Place of birth (Aluminium concentration in drinking water)	All data concerning history of exposure in both cases and controls were obtained from informants; place of birth was connected to historical data on aluminum levels in drinking water	Controls were randomly selected from the population of the northern health region; for each case, a family health service authority (FHSA) was chosen randomly, the authority then supplied the names of five individuals of the same sex and age as the case; if needed a replacement control from the potential list of five was selected randomly; the possibility of dementia in the control was excluded
Frecker	89	1991	7238 (399 dementia)	70.4% women in north shore, 53.8% women in rest of the bay	Bonavista bay, Newfoundland, Canada	Born in the early 1900's; death certificates registered in 1985 and 1986 at the provincial Department of Health	Place of birth (Drinking water quality)	Place of birth from death certificate; aluminum in drinking water and other measures of drinking water quality were performed	1985 and 1986 death of individuals born in Bonavista bay surviving beyond age 70 years, and not having a dementing illness recorded on their death certificates
Jean	82	1996	235 cases	65/170 for cases	Saguenay-Lac-Saint-Jean (SLSJ), Quebec, Canada	Cases from project IMAGE, comparison with whole SLSJ reference population	Place of birth (rural vs urban)	For cases, close family members acted as informants for place of birth; registry data for the reference population<br><br>SLSJ region was divided into urban and rural sub-regions	Data from the reference population was obtained through the Institut interuniversitaire de recherches sur les populations (IREP), all live births that occurred in the SLSJ within the study period

Prince	36	1994	273	Not reported	England, Scotland and Wales	Participants form primary care practices participating in the MRC hypertension trial	Place of birth (area of residence from birth to age 15; rural vs urban)	Risk factor questionnaires by MRC research nurses to the subject and an informant for each participant	Unimpaired, unmatched control from same population with intact PALT scores
Cohort studies									
Gilsanz	85	2017	7423	3374/4049	San Francisco bay area, USA	Member population of health care delivery system (KPNC), that participated in optional check-ups between 1964 and 1973	Place of birth (in high stroke mortality state)	Place of birth was captured during an optional check-up, asking 'where were you born?'<br><br>High stroke mortality states were determined by using the overall Centers for Disease Control and Prevention stroke mortality rates from 2012 to 2014; high stroke mortality states were those in the top quintiles of stroke mortality rates among US territories (>83 stroke deaths per 100 000 people older than 34)	KPNC members born outside high stroke mortality state
Gilsanz	88	2019	6268	2869/3399	San Francisco bay area, USA	Member population of health care delivery system (KPNC), that participated in optional check-ups between 1964 and 1973	Place of birth (in high infant mortality rate areas)	Place of birth was captured during an optional check-up, asking 'where were you born?'<br><br>State level infant mortality rates were obtained from a historical report issued by the US census bureau from 1928; infant mortality rates were determined separately for white and African-American individuals	KPNC members born outside high infant mortality rate areas
Glymour	86	2011	1393733	Not reported (Comparable to US population)	USA	The 2000 US Census Public, individuals who were born in the District of Columbia or any of 49 US states (Excluding Hawaii), self-reporting race as African-American or white; upweighted to represent total US population	Place of birth (Stroke belt)	Place of birth in a 'stroke belt' state, based on the householder who filled in birthplace at the 2000 US census report.	Individuals born outside the stroke belt
Guaita	92	2015	1321	607/714	Abbiategrasso, Italy	InveCe.Ab study, residents from Abbiategrasso born between 1935 and 1939	Place of birth (location in Italy)	Collected from municipal registry and/or from a social questionnaire administered by trained interviewers*	-
Scazufca	84	2008	2005	784/1221	Butantã, Sao Paulo, Brazil	Sao Paulo Ageing & Health Study (SPAH); participants lived in 66 pre-defined census sectors of the borough of Butantã with the lowest Human Development Index in the borough.	Place of birth (city, town or rural)	Through interview with participant and a key informant (co-residents, a relative or friend who were familiar with the participants life history); participants were asked to name the place they were	-

						Participants were community-dwelling and recruited through door knocking		born (farm, town/village, city, district, state, country) and to classify that place as a city (known urban area), town (small village with reduced number of houses, shops and other amenities, and no more than a primary school) or rural area at the time of birth	
Topping	87	2021	353572	220503/133069	USA	Participants from the Diet and Health Study (DHS), including members of the American Association of Retired Persons.	Place of birth (stroke belt)	State of birth was based on the social security number asked on the initial survey. The stroke belt consists of states with high stroke mortality.	Individuals born outside the stroke belt
Wilson	91	2005	859	263/596	Chicago, USA	Older catholic nuns, priests and brothers from the religious orders study; mean education of 18.1 years.	Place of birth (county SES level)	Participants (at baseline) were asked the country, state, county and city of their birth; information from the 1920 census (1 in 200) was used to determine the county average Duncan SES index for head of household, literacy rate (in those aged 6 or older), and proportion of 6-13 year old in school	-

Basic information extracted from studies regarding place of birth.

[a]This number represents the participants included in the analyses relevant for our systematic review or, if this specific number was not available, the total number of participants in the study. If the study did not include a control group but used a reference population as a comparison, or if the number of controls was not available, only the number of cases was reported.

*Reporting was not complete or not clear.

AD=Alzheimer’s disease

Other factors

Author	Ref.	Publication year	N[a]	Men/women	Location	Population	Exposure	Exposure ascertainment	Comparison/control
**Other factors**									
Case-control studies									
Jiang	97	2020	139	66/73	Not reported; most likely California, USA	Recruited from community adult care centers and assisted living facilities.	Right hand second to fourth finger length ratio as an indication of prenatal sex hormone exposure (2D:4D)	Images of hands were collected using a scanner. 2D:4D ratios were measured on collected images using a Vernier caliper by a single assessor who was blinded to the group designation of the participants.	Controls were from the same population self-reported to have no severe memory impairment or dementia.
Vladeanu	96	2014	40	18/22	London, UK	As part of a larger investigation at the Hillingdon Memory Cilnic of the Central and North West London National Health Service trust; all were British citizens with English as their first language	The second to fourth finger length ratio as an indication of prenatal sex hormone exposure	Digital calipers to measure the lengths of the second and fourth fingers, from the crease at the base of the finger to the tip, measured only once; the 2D:4D ratio was calculated for each hand separately and for both hands averaged	Recruited through advertisements in local media; no report of head injury or any diagnosis of a psychiatric disorder or dementia; 16/20 controls received the Addenbrooke Cognitive Examination Revised to test for undiagnosed dementia
Cohort studies									
Cocoros	93	2018	284397	136511/147886	Denmark	Danish-born residents; registry data	Prenatal 1918 influenza pandemic exposure	By date of birth, having experienced at least one peak of the 1918 influenza virus in utero, based on Danish surveillance data to identify peaks in the epidemic	Unexposed group had birth dates from December 1915 through June 1918 (born before influenza peak) or March through December 1921 (born after the influenza peak and were not in utero during the influenza peak)
Kang	94	2017	6790	2778/4012	Sichuan province, China	From rural and urban areas in China	Prenatal famine exposure	Based on date of birth; Great Chinese Famine occurred from 1959 to 1961: pre-famine group born between 1956 and 1958, famine group born between 1959 and 1961, post-famine group born between 1962 and 1964	Pre-famine group and post-famine group
Lenz	95	2018	Data from 23 nations were included	Not reported	23 countries: Australia, Austria, Belgium, Canada,	Analysis done at nation level; participants were mostly white	The second to fourth finger length ratio as an indication of prenatal sex	Online self-measure method for length of the second and fourth fingers; used the mean of the national right-hand and left-hand 2D:4D values; measured in individuals aged 10 to 70 years	Comparison across countries (country averages)

					Croatia, Czech Republic, Denmark, Finland, France, Germany, Hungary, Iceland, Ireland, Italy, Netherlands, New Zealand, Norway, Romania, Spain, Sweden, Switzerland, United Kingdom, and the United States of America		hormone exposure (2D:4D)		
Luo	98	2020	43254	20632/22622	Sweden	Twins form the Swedish Twin Registry born between 1906 and 1957.	Presumed differential prenatal hormone exposure by comparing same- and opposite- sex dizygotic twin pairs	Based on the Swedish Twin Registry. Comparing those form opposite-sex twin pairs to same-sex dizygotic twin pairs, for men and women separately.	-

Basic information extracted from studies regarding other factors.

[a]This number represents the participants included in the analyses relevant for our systematic review or, if this specific number was not available, the total number of participants in the study. If the study did not include a control group but used a reference population as a comparison, or if the number of controls was not available, only the number of cases was reported.

*Reporting was not complete or not clear.

Birth characteristics

Author	Ref.	Publication year	N[a]	Men/women	Location	Population	Exposure	Exposure ascertainment	Comparison/control
**Birth size**									
Cohort studies									
Matshusima	99	2018	269	Not reported	Hiroshima and Sendai, Japan	From the Japanese Study of Age and Retirement (JSTAR); baseline sample were individuals aged 50 to 75 who were randomly chosen based on household registration in a total of 10 selected municipalities	Low birth weight/premature birth	Respondents were asked in a questionnaire to fill in their birth weight (in kilograms), if they had it recorded in a birth record handbook or if someone told them and if they did not know the weight, they were requested to answer whether they were a premature baby or not	Individuals not reporting low birth weight or being born preterm
Mosing	65	2018	35191	16324-16813/ 17842-18378	Sweden	All twins, from the Swedish Twin Registry, with information on birth characteristic, born between 1926-1960; excluded early onset dementia (<55 yeas)	Birth weight<br>Head circumference at birth<br>Birth length<br>Gestational age<br>Birth SES	Based on nationwide collection of information from original birth records, recorded by midwives and/or doctors at the time of birth	Dependent on analysis (e.g individuals not being born small for gestation age)
Syddall	100	2005	37615	21632/15983	Hertfordshire, England	Hertfordshire Cohort study: all births from 1911 were reported by the attending midwife and recorded in ledgers; individuals that could be traced were included	Birth weight	Computerized midwife ledgers; birth weight was measured in pounds and ounces and had been converted to metric units, the midwives typically recorded weights to the nearest quarter pound	-

Basic information extracted from studies regarding birth charateristics.

[a]This number represents the participants included in the analyses relevant for our systematic review or, if this specific number was not available, the total number of participants in the study. If the study did not include a control group but used a reference population as a comparison, or if the number of controls was not available, only the number of cases was reported.

*Reporting was not complete or not clear.

SES=Socioeconomic status

Table B – Information and Results

Parental age and birth order

Author	Ref.	Outcome: Type	Outcome: Measurement/criteria	Outcome: Age; mean (SD; range)	Statistical test	Covariates accounted for in model or by matching	Exposure	Results (by exposure): Unadjusted/crude model	Results (by exposure): Fully adjusted model	Key limitations
**Parental age and birth order**										
Case-control studies										
Amaducci	60	Alzheimer's disease	Slow and progressive decline assessed with the Blessed Dementia Scale	(41-80)	Matched pairs odds ratio and McNemar's test (corrected for continuity)	Matched on: age (±3 years), sex and region of residence	Maternal age >40<br>Paternal age >44<br>Birth order ≥2<br>Birth order ≥4	OR 4.67 (p=0.01)<br>OR 4.50 (p=0.06)<br>OR 1.28 (p=0.53)<br>OR 1.88 (p=0.06)	-	-Extensive multiple testing
Bertram	45	Alzheimer's disease	ICD-10 criteria and NINCDS-ADRDA criteria for probable AD	Cases high MGAD 70.8 (10.9); low MGAD 70.3(9.0) Controls 70.3 (8.9)	Independent student's t-test	Matched on: age and gender	Maternal age<br>Paternal age	NS<br>NS<br><br>*(subgroup results in table C)*	-	-Statistics insufficiently reported
Chandra	63	Alzheimer's disease (onset after 70)	Short portable mental status questionnaire and medical records; NINCDS-ADRDA criteria for probable AD	Cases 83.2 (70-96); Controls 83.0	Matched pairs odds ratio and McNemar's test	Matched on: relationship with surrogate respondent, age (within 3 years), race, sex	Birth order ≥4	OR 1.0, X^2=0.00 (NS)	-	-Multiple testing<br>-Only late age of onset
Clarnette	46	Alzheimer's disease	MMSE and patient history; NINCDS-ADRDA criteria for possible or probable AD	Not reported	Unpaired t-test and odds ratios with 5 year maternal/paternal age groups	-	Maternal age<br><br><br><br><br><br><br><br>Paternal age	t-test means NS<br>16-20: OR 0.78 (0.31-2.07)<br>21-25: OR 1.33 (0.67-2.62)<br>26-30: reference<br>31-35: OR 1.97 (0.85-4.42)<br>36-40: OR 0.82 (0.35-2.11).<br>41+ OR 2.59 (0.87-10.08)<br><br>t-test means NS	-	-No strict protocol for ascertainment of exposure<br>-Not accounting for covariates

								16-20: OR 0.95 (0.23-4.27)<br>21-25: OR 1.07 (0.48-2.35)<br>26-30: OR reference<br>31-35: OR 1.08 (0.49-2.35)<br>36-40: OR 3.15 (1.20-7.79)<br>41+ OR 1.48 (0.6-3.6)<br><br>*(subgroup results in table C)*		
Cohen	43	Alzheimer's disease	Classified according the research criteria for AD; extensive list of diagnostic criteria mentioned	Cases 73.2	None, only means reported	-	Maternal age	Cases mean 35.5 (SE=1.4)<br>Controls mean 27	-	-Comparison group not clearly described<br>-No statistical analysis
Corkin	42	Alzheimer's disease	Neurologic examination; extensive list of diagnostic criteria mentioned	Cases 63.5 (52-78)<br>Controls 63.8 (52-80)	None, only means reported	No matching, they state control data were obtained from individuals of comparable age and SES	Maternal age<br><br>Paternal age	Cases mean 27.7 (18-33)<br>Controls mean 27.4 (17-40)<br><br>Cases mean 30 (21-50)<br>Controls mean 29.4 (18-39)<br><br>*(subgroup results in table C)*	-	-No statistical analysis
De Braekeleer 1988	44	Alzheimer's disease (clinical or autopsy proven)	Definite, probable or possible AD according to NINCDS-ADRDA criteria	Not reported	Two tailed student's paired t-test. Haldane-Smith test (birth order)	Dependent on comparison:<br><br>Siblings and spouses: no matching<br><br>Controls 1-2-3 matched on: date of parental marriage (+/- 5 days), parental SES, parental residence, birth rank<br><br>Controls 4-5-6 matched on: Sex, year of birth, SES	Maternal age<br><br>Paternal age<br><br>Birth order	Cases mean 29.4 (SD 6.7)<br>Siblings mean 31.5 (SD 4.1)<br>F=2.64 p<0.001<br>Vs spouses (mean 29.5 (SD 6.1) or controls p>0.10<br><br>Cases mean 32.6 (SD 7.5)<br>Siblings mean 34.9 (SD 5.0)<br>F=2.25 p=0.001<br>Vs spouses (mean 33.4 (SD 6.7) or controls p>0.10<br><br>Test-statistic: -3.075, p>0.99 (no birth order effect vs later born alternative), no birth order effect vs early birth order effect (p<0.001)	-	-Limited reporting
English	47	Alzheimer's disease	Thorough physical and psychiatric examination	Not reported	Chi-squared test for trend; Mantel's test for trend to include covariates; 95% CIs for odds ratios calculated	Adjustment for mother's year of birth (10 year interval)	Maternal age	<25 reference<br>25-29: OR 0.4 (0.17-1.1)<br>30-34: OR 1.0 (0.38-2.4)<br>35-39: OR 0.7 (0.21-2.3)<br>40+: OR 1.4 (0.22-9.1)<br>p=0.82	Similar results, not reported	-Low response rate<br>-Method of exposure ascertainment different for cases and controls

					by Cornfields method			*(subgroup results in table C)*		
Farrer 1991	33	Alzheimer's disease	Probable AD according to NINCDS-ADRDA criteria	Not reported; age at onset 67.2 (42-86)	Matched design: paired comparison t-test and conditional logistic regression	Matched on: sex, year of birth (same year), 'survival' age and *residence<br><br>Included in conditional logistic model: parental and maternal age in one model*	Maternal age<br><br>Paternal age	AD mean 27.4 (SE 0.4)<br>Control 27.9 (SE 0.2) NS<br>OR 1.04 (0.89-1.23) (5-year steps)<br><br>AD 30.5 (SE 0.5)<br>Control 31.6 (SE 0.2) NS<br>OR 0.88 (0.76-1.01) (5-year steps)<br><br>*(subgroup results in table C)*	-	-Limited reporting
Farrer 1997	59	Alzheimer's disease (clinical or autopsy proven)	Definite or probable AD according to NINCDS-ADRDA criteria	Not reported	Least square mean/Duncan multiple range test (taking matching into account)<br><br>Conditional logistic regression analysis	Matched on: gender, year of birth (wide intervals), 'survival' age<br><br>Adjusted for:<br>(1) Gender, age/year of birth (least square means)<br><br>(2) APOE genotype, maternal/paternal age (logistic regression)	Maternal age<br><br>Paternal age	Cases mean 28.9 control 28.1 p<0.02<br>OR 1.28 (1.08-1.48) (10 year steps)<br><br>Cases mean 32.1 controls 31.0 p=0.004<br>OR 1.22 (1.06-1.40) (10 year steps)<br><br>*(subgroup results in table C)*	OR 1.12 (0.88-1.41) (10 year steps)<br><br>OR 1.16 (0.96-1.41) (10 year steps)	-AD cases form clinic<br>-Concerns of recruitment bias<br>-There might be some overlap with participants from Farrer 1991
Forster	48	Presenile Alzheimer' disease (onset <65)	Hospital cases notes were studied; DSM-III-R criteria for dementia, NINCDS-ADRDA criteria for Alzheimer's disease	Not reported; diagnoses <65	OR's calculated with McNemar's test (taking matching into account)	Matched on: age and sex	Maternal age	Maternal age >=40 0.4 95%CI 0.1-1.59 p=NS		-Multiple testing<br>-Only included early onset AD
Fratiglioni	61	Alzheimer's disease	MMSE screening, DSM III-R criteria with some modifications; mild, moderate or severe dementia according to CDR scale	>75	ORs, Logistic regression	Adjustment for: age, sex, education, type of informant<br><br>Birth order: also adjusted for number of siblings	Maternal age<br><br>Paternal age<br><br>Birth order	<25 reference<br>25-34 OR 2.1 (1.0-4.5)<br>35+ OR 2.0 (0.6-4.5)<br><br><25 reference<br>25-34 OR 0.9 (0.4-2.1)<br>35+ OR 1.5 (0.7-3.6)<br><br>2nd vs 1st OR 1.2 (0.7-2.03)<br>4+ vs 1st-3th OR 1.3 (0.8-2.2)	<25 reference<br>25-34 OR 2.2 (1.0-4.9)<br>35+ OR 2.0 (0.8-4.7)<br><br><25 reference<br>25-34 OR 1.0 (0.4-2.3)<br>35+ OR 1.6 (0.7-3.9)<br><br>2nd vs 1st OR 1.0 (0.6-1.8)<br>4+ vs 1st-3th OR 1.0 (0.5-1.9)	

Graves	49	Alzheimer's disease	MMSE ≤ 26<br>DSM-3 and NINCDS-ADRDA criteria	Cases 66.2<br>Controls 63.6	Paired t-test, intraclass correlation coefficient<br><br>Conditional logistic regression	Matched on: sex, age (10 years), relationship with surrogate<br><br>Included in adjusted model: Age of subject at the reference year	Maternal age<br><br><br><br><br>Paternal age	Cases mean 28.57 (SD=6.66)<br>Controls mean 28.01 (SD=6.42)<br>Paired t=0.55, p=0.58, ICC=0.84<br><br>Cases mean 33.35 (SD=8.59)<br>Controls mean 31.35 (SD=7.27)<br>Paired t=1.58 p=0.12, ICC=0.92	Not different compared to unadjusted analysis, not reported	-Control pairs were chosen by the case surrogate respondent, process not documented<br>-Multiple testing
Heyman	41	Alzheimer's disease (onset <70)	Uniform battery of diagnostic tests and procedures; MMSE	Not reported; age of onset <70	Paired t-test	Matched with spouses	Maternal age<br><br>Paternal age	Mean cases 28.6 controls 28.4 NS<br>Mean cases 33.3 and 31.9 NS		-Only AD cases with onset <70<br>-Limited reporting<br>-Multiple testing<br>-Not accounting for confounders
Hofman	50	Alzheimer's disease (onset <70)	Clinical dementia rating scale, extensive clinical and neurological examination	Not reported; <70 at diagnosis	Logistic regression, matched-pair case-control analysis	Included in model: gender, age and area of residence<br><br>Matching on: gender, age (5 year categories) and area of residence	Maternal age<br><br><br><br><br><br><br><br>Paternal age<br><br><br><br><br><br><br><br><br><br><br>Birth order	-	≤24 reference<br>25-29 RR 0.9 (0.5-1.6)<br>30-34 RR 0.9 (0.5-1.6)<br>35-39 RR 0.6 (0.3-1.1)<br>40+ RR 1.1 (0.5-2.6)<br>Difference in mean age 0.7 (SEM 0.7) 95%CI (-0.7-2.1)<br><br>≤24 reference<br>25-29 RR 0.9 (0.5-1.9)<br>30-34 RR 0.7 (0.4-1.5)<br>35-39 RR 0.4 (0.2-1.0)<br>40+ RR 0.9 (0.4-1.8)<br>Difference in mean age 1.5 (SEM 0.8) 95%CI (-0.1-3.1)<br><br>*matched pair analysis gave similar results.*<br><br>NS<br><br>*(subgroup results in table C)*	-Only AD cases with onset <70<br>-Limited reporting
Jouan-flahault	54	Dementia	Medical examination after indication of mental	Not reported; ≥65 years	T-test	Matched on: age and sex	Maternal age<br><br>Paternal age	Mean cases 28.5 controls 28.2 NS	-	-Limited reporting

			deterioration at home interview					Mean cases 31.6 controls 33.0 NS		
Knesevich	51	Alzheimer's disease (clinical diagnosis or autopsy proven)	Diagnostic criteria (Berg et al.); additional subjects with autopsy proven Alzheimer's disease	Not reported	T-test (maternal age), Slater method (birth order)		Maternal age<br><br>Birth order	NS<br><br>Cases mean 0.41 (on a 0-1 scale; SE 0.082)<br>Controls mean 0.47 (on a 0-1 scale; SE 0.083) NS	-	-Limited sample-size<br>-Comparison with population form a different decade<br>-Limited reporting
Li	64	Alzheimer's disease (onset >40)	NINCDS-ADRDA criteria for probable AD and ICD-10 criteria for dementia in AD; all followed for 6 months	Cases 65.2, Controls 65.4	1:2 matched set method of analysis, OR	Matched on: age (3 years) and sex	Birth order ≥4<br>Birth order ≥6	OR 1.47 (0.73-2.97)<br>OR 2.75 (0.71-10.67)	-	-Extensive multiple testing<br>-Young patients
Lindsay	40	Alzheimer's disease	Modified MMSE (3MS), clinical examination if indication by MMSE. DSM-III-R criteria + NINCDS-ADRDA criteria for probable AD	Cases 84.7 Controls 79.0	Unconditional logistic regression	Frequency matched on: age group, study center and residence in community of institution.<br><br>Included in model: Age, sex, education, residence in community or institution.	Maternal age<br><br>Paternal age	-	<20 1.86 95%CI 0.52-6.61<br>20-29 reference<br><br>>40 OR 1.49 95%CI 0.71-3.16<br>20-29 reference	-Limited reporting<br>-Extensive multiple testing
Moceri 2000	52	Alzheimer's disease	DSM-3th revised and definite or probable AD according to NINCDS-ADRDA criteria	Most between 60-9; mean age at intake cases 78 (6.7) controls 78 (6.8)	Unconditional logistic regression	Frequency matched on: sex and age (2 years)	Maternal age<br><br>Birth order	<20 OR 1.02 (0.53-1.95)<br>20-34 referent<br>35+ 0.89 (0.71-1.12)<br><br>OR 1.04 (0.96-1.12)	-	-Multiple testing
Moceri 2001	35	Alzheimer's disease	DSM-3th revised and probable AD according to NINCDS-ADRDA criteria	Most between 60-89; not clearly reported	Unconditional logistic regression	Frequency matched on: sex and age (2 years)	Maternal age<br><br>Paternal age<br><br>Birth order	<20 OR 0.75 (0.28-1.95)<br>20-24 reference<br>35+ OR 0.86 (0.72-1.19)<br><br><20 OR 1.07 (0.00-39.61)<br>20-34 reference<br>35+ OR 1.11 (0.75-1.63)<br><br>1st reference<br>2nd 1.58 (0.95-2.61)<br>3rd 1.12 (0.62-2.02)<br>4th 1.24 (0.74-2.09)	-	-Multiple testing<br>-There might be some overlap with participants included in Moceri 2000

Prince	36	Alzheimer's disease and other dementias (onset > 65)	DSM-III-R criteria for dementia including AD, multi-infarct dementia, mixed vascular and AD and amnestic syndrome. Possible or probable AD according to NINCDS-ARDA criteria	71-87	Odds ratios; logistic regression for adjusted analysis	Included in adjusted model: age, sex, family history of dementia, premorbid IQ and area of residence	Maternal age >34<br>Paternal age >37	NS<br>NS	NS<br>NS	-Very limited reporting
Ptok 2000	37	Alzheimer's disease	Possible AD according to NINCDA-ADRDA criteria; CIDI and SIDAM interviews	>60<br>AD patients 76.99 (9.66)<br>Controls 76.99 (9.66)	ANOVA	Included in adjusted model: gender<br><br>Matched on: age, gender and education	Maternal age<br><br><br><br><br><br><br><br>Paternal age	AD mean 28.13 (SD 5.59 95%CI 26.60-29.66)<br>Depression and AD 29.13 (SD 5.01 27.32-30.93)<br>Controls 28.10 (SD 5.32 26.98-29.23)<br>F=0.548 df=4 p=0.701<br><br>AD 32.67 (SD 8.70 30.14-35.19)<br>Depression and AD 32.80 (SD 6.86 30.24-35.36)<br>Controls 31.79 (SD 5.84 30.54-33.04)<br>F=0.292 df=4 p=0.88	No relevance of the subject's gender was detected	-Limited reporting<br>-Low availability of parental age data
Tsolaki	38	Alzheimer's disease	DSM-IV and NINCDS-ADRDA criteria for probable AD	>70	Chi-square (Yates's correction if necessary), logistic regression, cluster analysis	Age-matched?*	Maternal age (>35)<br><br><br><br><br>Paternal age (>35)	Chi-squared p=0.8<br>Cases: 21 women, 7 men<br>Controls: 13 women, 4 men with maternal age >35<br><br>Chi-squared p=0.92<br>Cases: 11 women and 12 men<br>Controls: 8 women and 10 men with paternal age >35	-	-Multiple testing<br>-Limited reporting related to case/control selection
Urakami	53	Alzheimer's disease, Multi-infarct dementia (MID)	DSM-III and NINCDS-ADRDA criteria; Hachinski ischemia score to distinguish AD from MID, followed at least 3 years	AD 80.78 (7.64)<br>MID 78.46 (8.90)<br>Controls normal cognition 77.97 (7.52)<br>Controls subnormal cognition 78.07 (7.52)	Student's t-test		Maternal age<br><br><br><br><br><br><br>Paternal age	AD 26.58 (SD 6.63)<br>MID 24.12 (SD 5.73)<br>Normal 23.86 (SD 4.85)<br>Subnormal 25.00 (SD 5.46)<br>AD vs MID p<0.02<br>AD vs normal sign. higher<br><br>AD 31.44 (SD 8.65)<br>MID 27.21 (SD 6.99)<br>Normal 26.29 (SD 5.65)<br>Subnormal 27.91 (SD 6.08)	-	-Limited reporting<br>-Limited numbers

								AD vs normal p<0.001<br>AD vs subnormal p<0.01<br>AD vs MID sign. higher		
Whalley 1982	62	Alzheimer's disease (autopsy proven)	Admission to mental hospital before age 65; clinical characteristics; neuropathological evidence from autopsy	Age at death, men 62.3 (SD 6.7), women 64.4 (SD 4.7)	Paired students t-test (parental age)<br><br>Tendency to be born later with method of Carothers et al. (birth order)	1:3 matched for date of entry birth record, sex and social class	Maternal age	Cases mean 30.6 (SE 0.7)<br>Controls mean 28.6 (SE 0.4)<br>p=0.014<br>Sibling control NS	-	-Limited reporting
							Paternal age	Cases mean 33.2 (SE 0.8)<br>Controls mean 30.8 (SE 0.5)<br>p=0.005<br>Sibling control NS		
							Birth order	NS		
Whalley 1995	34	Alzheimer's disease, vascular dementia (symptoms before 65)	Probable presenile AD according to McKhann criteria; Hachinski score greater than 6 and at least one cerebrovascular accident additional criteria for vascular dementia	<73; symptoms present before 65	Conditional logistic regression	Matched on: sex, birth registration district, date of birth (same year), father's occupational group<br><br>Included in adjusted model: parental and maternal age in one model	Maternal age	AD mean 30.17 (SD 6.27)<br>controls 28.82 (SD 6.14) NS<br>VaD mean 28.80 (SD 6.03)<br>controls 28.88 (SD 6.02) NS	AD Likelihood ratio 0.303<br>df=1 p=0.58<br>VaD NS	
							Paternal age	AD mean 33.58 (SD 7.44)<br>controls 31.53 (SD 6.84)<br>p<0.001<br>VaD mean 31.53 (SD 6.69)<br>controls 31.89 (6.87) NS<br><br>*(subgroup results in table C)*	AD 1.05 (1.02-1.09)<br>VaD NS	
White	39	Alzheimer's disease (clinical and autopsy proven)	Identified from autopsy records. Secondary cases identified from relatives and diagnosed using clinical criteria	Not reported	One tailed t-test, Sibling analysis	No direct adjustment, they performed a sibling analysis to indirectly accounts for SES and education	Maternal age	AD mean 29.1<br>normal siblings 27.5<br>t(202)=1.50 df=202 p=0.07<br>controls 30.0<br>t(310)=-1.16 p>0.05	-	-Statistic method not clearly described
							Paternal age	AD mean 34.2<br>normal siblings 32.9<br>t(203)=0.98 p>0.10<br>controls 34.7<br>t(311)=-0.59 p>0.50		
							Birth order	AD mean 3.10<br>expected average throughout sibship t(120)=1.20 p=0.11<br>Controls mean 4.20<br>t(324)=3.50 p<0.01		
Cohort studies										

Katzman	55	Alzheimer's disease	If a change in cognition or function was noted, or if there was an increase of four errors or more on the IMC or if the total errors exceeded eight, a dementia work-up was carried out. DSM-III and McKhannn criteria<br><br>5-year follow up period	79 (75-85) at intake	Chi-squared test	-	Maternal age	Mean maternal age at birth<br>Alzheimer cases: 25<br>No dementia: 26<br><br>NS	-	-Limited reporting<br>-Results/statistics insufficiently reported for our exposure of interest
Kim	66	Dementia	Consensus diagnosis of dementia and subtypes were made by three psychiatrists and a neurologist, according to DSM-IV NINCDS-ADRDA and NINDS-AIREN criteria	Cases 77.9 (6.8)<br>Controls 73.1 (5.2)<br>(>65)	T-test	-	Birth order	Mean (SD) birth order<br>No dementia: 2.5 (1.6)<br>Dementia: 2.5 (1.7)<br>t=-0.48<br>p=0.631	-	-Not accounting for older age in dementia group<br>-Primary focus was not on birth order as an exposure (limited analysis/reporting)
Lahti	67	Dementia	Hospital Discharge Register and Causes of Death Register; ICD-8 (290.00-290.10), ICD-9 (290, 2912A, 2928C, 2941A, 3310A, 3311A, 4378A) ICD-10 (F00, F01, F03, F051, G30)	66-76	Cox proportional hazards models	Model 1: Adjusted for sex, year of birth (1934-1938 or 1939-1944), mother's and father's highest attained occupational statuses, birth weight, maternal body mass index at childbirth<br><br>Model 2: Adjusted for sex, year of birth (1934-1938 or 1939-1944), mother's and father's highest attained occupational statuses, birth weight, maternal BMI at childbirth, and maternal age at childbirth	Maternal grand multiparity (born as sixth or later born child)	Model 1:<br>HR 0.93 95%CI 0.29-2.98, p=0.91<br><br>*(subgroup results in table C)*	Model 2:<br>HR 1.09 95%CI 0.34–3.55, p=0.88<br><br>*(subgroup results in table C)*	-Number of individuals with dementia was really low<br>-No crude model presented<br>-Cohort was born around time of war, malnutrition was common, may limit generalizability to individuals born more recently.
Mosing	65	Dementia (clinical diagnosis or cause of death)	Register-based dementia diagnoses as well as date of first diagnosis were derived from the NPR and CDR. ICD 7-10 codes for	74.3 (2.6; 55-88)	Cox proportional hazard analysis; Corrected for correlation within twin pairs	Included in model: sex	Birth order (within twins)	NS (p>0.05)		-Registry data might miss a lot of dementia diagnosis<br>-Only twins were included

			AD, vascular dementia and other dementia							(population not representative)
Reisz	58	Dementia	Based on documented diagnosis of dementia in the patient's problem list, current prescription and use of medications used for Alzheimer's dementia, or endorsement of a dementia diagnosis by a caregiver present at the time of the clinic visit	77.12 (6.8; 65-98)	Logistic regression	Age	Maternal age<br>Birth order<br>Age difference with the next older sibling	OR 1.026 95%CI 0.963-1.09<br>OR 1.268 95%CI 0.988-1.61<br>OR 1.046 95%CI 0.896-1.205		-Only Caucasian women were included<br>-Exposure status based on patient recollection
Tyas	56	Alzheimer's disease	Individuals with MMSE score <78 were clinically evaluated for AD; NINCDS-ADRDA criteria for probable or possible AD<br><br>5-year follow up period	Cases 79.8 (5.7)<br>Controls 73.7 (5.7) at intake	Logistic regression	Included in adjusted model: age, sex and education	Maternal age<br><br><br><br>Paternal age	-	Cases mean 28.0<br>Controls mean 28.8<br>RR 0.97 95%CI 0.91-1.04<br><br>Cases mean 31.9<br>Controls mean 33.9<br>RR 0.95 95%CI 0.89-1.02	-Limited reporting<br>-Multiple testing<br>-Low number of AD cases
Vaillant	57	Dementia	Telephone Interview for Cognitive Status (TICS) (at age 80, 85 and 90) score <31 and clinical evidence for dementia (information from relatives, psychosocial course after 70, results from the physical exams conducted every five years, and death certificates)<br><br>Followed from 25-90, survived until at least 70	At least survived until 70	Spearmans's rho (two-tailed), logistic regression	Did not adjust for factors, however, this was a very uniform group of men	Maternal age	Spearmans rho<br>Dementia by age 90: 0.10 (NS)<br>Intact cognition (1) or dementia (0) at age 90 (n=84): -0.20 (NS)<br><br>Dementia vs intact cognition at age 90<br><br>OR 0.27 95% 0.06-1.13	-	-Limited reporting<br>-Sample highly educated, not comparable to general population

Information and results from studies regarding parental age and/or birth order.

[a]Results for hospital controls not shown.

*Reporting was not complete or not clear.

AD=Alzheimer's disease

CDR=Clinical Dementia Rating Scale

CIDI=Composite International diagnostic interview

DSM=Diagnostic and Statistical Manual of Mental Disorders

ICD=International Classification of Diseases.

IMC=The initial information-memory-concentration.

MGAD=Major gene probability for Alzheimer's disease

NINCDS-ADRDA=National Institute of Neurological and Communicative Disorders and Stroke and the Alzheimer's Disease and Related Disorders Association (now known as the Alzheimer's Association)

NINDS-AIREN=National Institute of Neurological Disorders and Stroke and the Association Internationale pour la Recherche et l'Enseignement en Neurosciences

NS=Not statistically significant

SES=Socioeconomic status

SIDAM= Structured Interview for the diagnosis of Dementia of the Alzheimer type, Multi-infarct dementia and dementias of other etiology according to ICD-10 and DSM-III-R

Season of birth

Author	Ref.	Outcome			Statistical test	Covariates accounted for in model or by matching	Exposure	Results (by exposure)		Key limitations
		Type	Measurement/criteria	Age; mean (SD; range)				Unadjusted/crude model	Fully adjusted model	
**Season of birth**										
Case-control studies										
Dysken	68	Alzheimer's disease (autopsy proven)	Post-mortem brain specimens were examined for large numbers of neuritic plaques and neurofibrillary tangles	Not reported	Chi-squared analysis: observed vs expected birth, individual quarter vs remaining quarters (yates correction) Edwards' procedure for estimating cyclic trends	-	Season of birth (by quarter)	AD observed birth quarter vs expected. Chi-squared (df:3) 2.07 NS<br><br>Per birth quarter, Chi-squared (df:1):<br>Jan-March: 1.03 NS<br>Apr-June: 051 NS<br>Jul-Sept: 1.03 NS<br>Oct-Dec: 0.20 NS<br><br>Edwards's method did not observe a peak period of AD births.<br><br>*(subgroup results in table C)*	-	-Not accounting for confounders, like birth years<br>-Limited reporting
Fratiglioni	61	Alzheimer's disease	MMSE screening, DSM III-R criteria with some modifications; mild, moderate or severe dementia according to CDR scale	>75	ORs, Logistic regression	Included in adjusted model: age, sex and education	Season of birth (birth in 1st quarter)	1st quarter vs rest of the year RR 1.4 (0.8-2.2)	1st quarter vs rest of the year RR 1.4 (0.9-2.3)	-
Frazee	74	Alzheimer's disease (clinical diagnosis or autopsy proven)	Cases listed as probable AD or AD verified by autopsy from the AD research centers of California database; time of diagnosis ≥65	Not reported. At least >65	Comparison of AD births per quarter/month vs expected births; chi-squared test	Expected births were based on the specific birth years in the case sample	Season (by quarter) and month of birth	Per quarter: chi-squared (2, n=1359)=0.26 NS<br><br>Disproportionate number of births in the first 4 months, chi-squared=3.90, p<0.05	-	Limited reporting

Henderson	69	Alzheimer's disease	Possible or probable AD according to NINCDS-ADRDA criteria	Not reported	Chi-squared test; Roger's method for cyclic trends	Frequency matched on: age and gender	Season of birth (per quarter)	Per quarter: cases vs controls: chi-squared=1.48, p=0.69<br>Cases vs general population: chi-squared=1.51, p=0.68<br>Roger's test: chi-square:2.18 p=0.34 (monthly also NS)<br><br>*(subgroup results in table C)*	-	-Limited reporting
Koch	75	Dementia (vascular and AD)	Dementia diagnosis from the psychogeriatric ward of the University clinic of Regensburg; between 1995 and 2004	Not reported	Chi-squared test, circannual Cosinor models	Births were normalized according to the mean birth year of the patient sample	Season of birth (Dec-Feb, March-May, -June-August, Sep-November)	No significant circannual variation in patient births P>0.05<br><br>Tendency towards higher mean birth frequencies in the fall for vascular dementia and winter for Alzheimer patients	-	-Limited reporting<br>-Results/statistics insufficiently reported
Lawlor	72	Alzheimer's disease	NINCDS-ADRDA criteria for probable AD	Not reported	Chi-squared test	Matched for age	Season of birth (first quarter)	Percentages of first-quarter births<br>AD: 27%<br>Controls: 20.9%<br>Census-derived data: 25.1%<br>NS	-	-Very limited reporting (letter to the editor)<br>-Results/statistics insufficiently reported
Philpot	73	Alzheimer's disease	Probable AD according to NINCDS-ADRDA criteria	Not reported	Chi-squared test for observed vs expected births. Roger's method for testing cyclic trends and Edwards' method for determining the peak quarter of birth	Age structure of the comparison sample was matched to the cases (by decade of birth)	Season of birth (per quarter)	Quarterly variation of births compared to controls: chi-squared=6.35, p<0.1 p>0.05<br><br>Comparison with remaining quarters: (df=1)<br>January-March: chi-squared=5.67, p<0.02 75 observed/59.1 expected<br>April-June: chi-squared=1.79, p>0.1 52 observed/60.9 expected<br>July-September: chi-squared=0.003, p>0.9<br>60 observed/60.3 expected<br>October-December: chi-squared=1.83, p>0.1<br>52 observed/58.6 expected<br><br>*(subgroup results in table C)*	-	-Limited reporting

Prince	36	Alzheimer's disease and other dementias (onset > 65)	DSM-III-R criteria for dementia including AD, multi-infarct dementia, mixed vascular and AD and amnestic syndrome; possible or probable AD according to NINCDS-ARDA criteria	71-87	Odds ratios; logistic regression for adjusted analysis	Included in model: age, sex, family history of dementia, premorbid IQ and area of residence	Season of birth	NS	NS	-Very limited reporting
Ptok 2001	70	Alzheimer's disease	ICD-10 or DSM-III-R diagnosis; CIDI and SIDAM interviews, thorough clinical examination	AD 76.99 (9.66), co-morbid AD and depression 73.45 (9.58), controls 76.99 (9.66)	Chi-squared test, logistics regression to examine the effect of birth in the first three months of the year	Included in model: age, sex, ApoE	Season of birth (quarterly birth rates)	AD vs controls chi-squared: 2.99 (df=3) p=0.39<br><br>*(subgroup results in table C)*	Birth in the first three months of the year had no effect on AD (in the adjusted logistic regression analyses)	-Limited reporting
Tolppanen	76	Alzheimer's disease	From the finish reimbursement register; all records of individuals eligible for higher reimbursement due to certain chronic diseases, including AD<br><br>Must be diagnosed following NINCDS-ADRDA and DSM-IV criteria for AD	34-105<br><br>For cases 80.1 (71)	Conditional logistic regression	Matched on: age (+/- 1 year), sex and region of residence	Month and season of birth	Jan = reference<br>Feb: OR 1.02 95%CI 0.98-1.06<br>Mar: OR 0.99 95%CI 0.95-1.03<br>Apr: OR 1.02 95%CI 0.98-1.07<br>May: OR 1.02 95%CI 0.98-1.06<br>Jun: OR 1.02 95%CI 0.98-1.06<br>Jul: OR 1.04 95%CI 1.00-1.08<br>Aug: OR 1.00 95%CI 0.97-1.04<br>Sep: OR 1.02 95%CI 0.98-1.06<br>Oct: OR 1.00 95%CI 0.96-1.04<br>Nov: OR 0.99 95%CI 0.95-1.04<br>Dec: OR 0.97 95%CI 0.93-1.01<br>p=0.09<br><br>Winter (nov-feb) Reference<br>Spring (march-may) 1.02 95% CI 0.99-1.04<br>Summer (june-august) 1.03 95%CI 1.00-1.05		-Also included very young AD patients

								Autumn (sep-oct) 1.02<br>95%CI 0.99-1.04<br>P=0.13		
Vezina	80	Alzheimer's disease	NINCDS-ADRDA criteria for possible, probable and definite AD	Not reported	Chi-squared test for observed vs expected births	Included in adjusted model: birthyear (3-year periods)<br><br>In the comparison to the SOREP control group everyone was born in the SLSJ area	Season of birth (month)	Jan<br>RAMQ p=0.28<br>SOREP p=0.82<br>Feb<br>RAMQ p=0.07 (cases > controls)<br>SOREP p=0.22<br>Mar<br>RAMQ p=0.90<br>SOREP p=0.97<br>Apr<br>RAMQ p=0.54<br>SOREP p=0.74<br>May<br>RAMQ p=0.01 (cases < controls)<br>SOREP p=0.02 (cases < controls)<br>Jun<br>RAMQ p=0.44<br>SOREP p=0.26<br>Jul<br>RAMQ p=0.54<br>SOREP p=0.97<br>Aug<br>RAMQ p=0.47<br>SOREP p=0.70<br>Sep<br>RAMQ p=0.53<br>SOREP p=0.76<br>Oct<br>RAMQ p=0.24<br>SOREP p=0.13<br>Nov<br>RAMQ p=0.60<br>SOREP p=0.34<br>Dec<br>RAMQ p=0.92<br>SOREP p=0.90<br><br>chi2=10.96, df=11, p>0.10<br><br>Daily distributions:	P-values were similar for the adjusted and unadjusted analyses; not clearly reported if reported p-values were for the adjusted or unadjusted analyses	-Limited reporting<br>-Multiple testing<br>-Low number of cases

								The deficit of births in May remains significant, but also includes some days in June		
Vitiello	71	Alzheimer's disease	McKhann criteria	Cases 67.4 (9.1; 42-87) at diagnosis. Controls 66.2 (8.3)	Chi-squared test; Edwards method for cyclic trend	No matching; age and gender were similar	Season of birth (quarterly birth rates)	Quarterly distribution: chi-squared=3.6 (df 3) p=0.31<br>Monthly distribution: chi-squared=11.9 (df 11) p=0.37<br><br>No significant cyclic trend (Edwards method)<br><br>*(subgroup results in table C)*	-	-Limited reporting
Cohort studies										
Ding	78	Dementia	Combination of self-report or report by family member (with a trained interviewer) and checked by doctors with more than 5-year clinical experience; ICD-10-AM codes: F00 AD, F01 vascular dementia, F02 dementia in other diseases classified elsewhere, F03 unspecified dementia	69.68 (SD 7.24 >60)<br><br>Dementia cases, 75.69 (8.29)	Logistic regression	Adjusted for: age, gender, education and annual household income per capita	Season of birth	-	Spring (Mar, Apr, May) OR 0.85 95%CI 0.72-1.01 p=0.062<br>Summer Reference<br>Autumn (Sep, Oct, Nov) OR 0.89 95%CI 0.76-1.05 p=0.165<br>Winter (Dec, Jan, Feb) OR 0.76 95%CI 0.65-0.90 p=0.001<br><br>*(subgroup results in table C)*	-Limited reporting
Doblhamer	77	Dementia	ICD-10 codes: G30, G31.0, G31.82, G23.1, F00, F01, F02, F03, F05<br><br>Individuals were followed over a one year period.<br><br>Those with a verified second occurrence in the following 3-years were considered valid dementia cases	>65	Logistic regression	Crude model: adjusted for age (5-year groups) and gender<br><br>Included in fully adjusted model: adjusted for age (5-year groups), gender, diabetes mellitus, hypertension, ischemic heart disease, cerebrovascular diseases, hypercholesterolemia, arterial fibrillation	Season of birth	Winter (Dec, Jan, Feb) OR 0.93 p=0.004<br>Spring (Mar, Apr, May) OR 0.96 p=0.167<br>Summer (Jun, Jul, Aug) reference<br>fall (Sep, Oct, Nov) OR 0.95 p=0.051	Winter: OR 0.93, p=0.004<br>Spring: OR 0.95, p=0.077<br>Summer reference<br>Fall: OR 0.95, p=0.070	-Limited reporting<br>-Short follow-up period

Hsu	81	Alzheimer's disease	ICD-9 code: 331.0; insurer's registry data.	Not reported	Walter & Elwood's (W&E) test to estimate the within-year fluctuations with 12-month periodicity	-	Month of birth	Jan RR 1.079<br>Feb RR 1.071<br>Mar RR 0.987<br>Apr RR 0.937<br>May RR 0.967<br>Jun RR 0.940<br>Jul RR 0.961<br>Aug RR 0.931<br>Sep RR 1.073<br>Oct RR 1.004<br>Nov RR 1.016<br>Dec RR 1.011<br><br>P (W&E <0.001)<br><br>(*subgroup results in table C*)	-	-Limited reporting
Mooldijk	79	All-cause dementia; Alzheimer's disease	Consensus panel (led by neurologist) using DSM-III-R and NINCDS-ADRDA criteria.	64.8 (9.6)	Cox-proportional hazard models	Model 1: adjusted for age and sex<br>Model 2: additionally adjusted for educational level<br>Model 3: additionally adjusted for ethnicity, income, use of alcohol, smoking, body mass index, blood pressure, total and high-density lipoprotein cholesterol, APOE genotype, history of diabetes, history of stroke, history of coronary heart disease, history of heart failure and depressive symptoms.	Season and month of birth; severity of winter	Model 1<br><br>All-cause dementia<br>Winter (Dec, Jan, Feb)<br>HR 1.15 (95%CI 1.01-1.31)<br>Spring (Mar, Apr, May)<br>HR 1.12 (95%CI 0.98-1.28)<br>Summer (Jun, Jul, Aug)<br>Reference<br>Fall (Sep, Oct, Nov)<br>HR 1.17 (95%CI 1.03-1.33)<br><br>Normal winter<br>HR 1.10 (95%CI 0.95-1.26)<br>Cold winter<br>HR 1.34 (95%CI 1.05-1.71)<br>Very cold winter<br>HR 1.35 (95%CI 0.99-1.84)<br>Summer<br>Reference<br><br>Alzheimer's disease<br>Winter (Dec, Jan, Feb)<br>HR 1.23 (95%CI 1.06-1.43)<br>Spring (Mar, Apr, May)<br>HR 1.14 (95%CI 0.98-1.33)<br>Summer (Jun, Jul, Aug)<br>Reference<br>Fall (Sep, Oct, Nov)<br>HR 1.15 (95%CI 0.99-1.35)	Model 3<br><br>All-cause dementia<br>Winter (Dec, Jan, Feb)<br>HR 1.13 (95%CI 0.99-1.29)<br>Spring (Mar, Apr, May)<br>HR 1.10 (95%CI 0.96-1.26)<br>Summer (Jun, Jul, Aug)<br>Reference<br>Fall (Sep, Oct, Nov)<br>HR 1.10 (95%CI 0.96-1.26)<br><br>Normal winter<br>HR 1.10 (95%CI 0.95-1.26)<br>Cold winter<br>HR 1.29 (95%CI 1.01-1.65)<br>Very cold winter<br>HR 1.34 (95%CI 0.98-1.83)<br>Summer<br>Reference<br><br>Alzheimer's disease<br>Winter (Dec, Jan, Feb)<br>HR 1.20 (95%CI 1.03-1.40)<br>Spring (Mar, Apr, May)<br>HR 1.13 (95%CI 0.96-1.32)<br>Summer (Jun, Jul, Aug)<br>Reference<br>Fall (Sep, Oct, Nov)<br>HR 1.08 (95%CI 0.92-1.26)	-

Information and results from studies regarding season of birth.

AD=Alzheimer’s disease

CDR=Clinical Dementia Rating Scale

CIDI=Composite International diagnostic interview

DSM=Diagnostic and Statistical Manual of Mental Disorders

ICD=International Classification of Diseases.

NINCDS-ADRDA=National Institute of Neurological and Communicative Disorders and Stroke and the Alzheimer’s Disease and Related Disorders Association (now known as the Alzheimer’s Association)

NS=Not statistically significant

SIDAM= Structured Interview for the diagnosis of Dementia of the Alzheimer type, Multi-infarct dementia and dementias of other etiology according to ICD-10 and DSM-III-R

Place of birth

Author	Ref.	Outcome			Statistical test	Covariates accounted for in model or by matching	Exposure	Results (by exposure)		Key limitations
		Type	Measurement/criteria	Age; mean (SD; range)				Unadjusted/crude model	Fully adjusted model	
**Place of birth**										
Case-control studies										
Baker	83	Alzheimer's disease	All individuals with cognitive impairment were evaluated; diagnosis were standardized with McKhann criteria	Not reported; most between 60 and 90	Conditional logistic regression	Matched on: age (within 5 years), sex, race marital status, status at time of surrogate interview (case alive or dead)	Place of birth (rural vs urban ?)*	OR 16.00 (1.91-*)<br><br>*Not meaningful due to small sample size	-	-Multiple testing<br>-Small sample
Emard	90	Alzheimer's disease	NINCDS-ADRDA criteria for probable or definite AD cases	Not reported	Multivariate analysis, factor analysis of correspondences and differences in residential and municipal geochemical concentrations (DRMC)	-	Place of birth (geochemical composition)	No single geochemical element seems to be associated with the spatial distribution of cases<br><br>For Pb, Mn and Fe there were significantly (p<0.05) more cases with higher concentrations at their birth place than the average municipal concentration than there were cases with lower concentrations	-	-No actual control group<br>-Limited reporting<br>-No direct comparison with geochemical concentrations
Forster	48	Presenile Alzheimer' disease (diagnosis <65)	Hospital cases notes were studied; DSM-III-R criteria for dementia, NINCDS-ADRDA criteria for Alzheimer's disease	Not reported; diagnoses <65	OR's calculated with McNemar's test (taking matching into account)	Matched on: age and sex	Place of birth (Aluminum concentration in drinking water)	Mean aluminum concentration at birth (n=160) (microgram/l)<br><50 1.3 95%CI 0.61-2.68 p=NS<br>>50 0.8 95%CI 0.37-1.64 p=NS<br>>99 0.8 95%CI 0.42-1.71 p=NS<br>>149 1.1 95%CI 0.38-3.35 p=NS<br><br>*(subgroup results in table C)*	-	-Multiple testing<br>-Only included early onset AD

Frecker	89	Dementia as a cause of death (death records)	1985 and 1986 death certificates were individually scanned for cause of death; dementia was defined by five possible categories: AD, chronic/organic brain syndrome, dementia, senile dementia/senile confusion, or senility	>70	Calculated difference in proportions with confidence intervals; Fleiss correction factor was used for proportions smaller than 0.1	Mortality rates were adjusted for age	Place of birth (drinking water quality)	Newfoundland population:<br><br>Two birth regions (03 and 07) had high dementia mortality rates (mean standardized rates, 56.5 and 54.4/100 000), Whole population 34 and 27/100 000 (1985 and 1986)<br>03=isolated region on south west coast<br>07=includes Bonavista bay and the North shore of Trinity bay<br><br>Bonavista bay:<br>1985 15 deaths from north (17.2% of all deaths in that area)<br>9 from central and south (6.3% from that area) 95% CI 1.1-20.7 for proportion<br><br>1986: 12 deaths (14.3%), 4 (2.9%) 95%CI 2.5-20.4 for proportion<br><br>In the Newtown area high incidence of dementia death coincided with the lowest pH, highest concentration of aluminum and highest water color measurement in drinking water; two other areas also had higher measures but no corresponding dementia increase	-	-Limited reporting<br>-No direct associations measured with drinking water quality
Jean	82	Alzheimer's disease (Clinical and autopsy proven)	Possible, probable or definite AD according to NINCDS/ADRDA criteria; definite cases were determined with autopsy	Not reported	Exact probability test	-	Place of birth (rural and urban vs reference population)	Rural vs reference population OR=1.29 (p<0.01)<br>Urban vs reference population OR=0.84 (p<0.01)<br><br>*(subgroup results in table C)*	-	-Limited reporting<br>-No correction for year of birth or other variables

Prince	36	Alzheimer's disease and other dementias (onset > 65)	DSM-III-R criteria for dementia including AD, multi-infarct dementia, mixed vascular and AD and amnestic syndrome; possible or probable AD according to NINCDS-ARDA criteria	71-87	Odds ratios; logistic regression for adjusted analysis	Included in model: age, sex, family history of dementia, premorbid IQ and area of residence	Place of birth (area of residence from birth to age 15; rural vs urban)	NS	-	-Very limited reporting
Cohort studies										
Gilsanz 2017	85	Dementia	Dementia diagnosis was ascertained from records from a health care delivery system (KPNC) (follow-up from Jan 1996 – Oct 2015)<br><br>Using ICD-9 codes: AD 331.0, vascular dementia 290.4x, other/nonspecific dementia (290.0, 290.1x, 290.2x, 290.3, 294.2x, 294.8)<br><br>Mean follow-up time was 11.61 (6.24)	Age in 1996 (start of follow-up) cases 71.18 (2.72), controls 71.14 (2.72)	Cox proportional hazards models with age as the time scale; cumulative risk of dementia	Included in crude model: age and sex<br><br>Included in fully adjusted model: age, sex, race, education, midlife (BMI, smoking duration, hypertension) and late life (diabetes, hypertension, heart failure, acute myocardial infarction and stroke) cardiovascular risk factors	Place of birth (in high stroke mortality state)	Age adjusted dementia incidence rate (per 1000 person years)<br>Outside high-stroke: 15.54 95%CI 14.42-16.66<br>Inside high-stroke: 23.56 95%CI 20.46-26.67<br>HR 1.54 95%CI 1.39-1.71<br><br>*(subgroup results in table C)*	Overall HR 1.27 95%CI 1.11-1.44<br><br>*(subgroup results in table C)*	-All participants migrated to California
Gilsanz 2019	88	Dementia	Dementia diagnosis was ascertained from records from a health care delivery system (KPNC) (follow-up from Jan 1996 – Oct 2015)<br><br>Using ICD-9 codes: AD 331.0, vascular dementia 290.4x, other/nonspecific dementia (290.0, 290.1x, 290.2x, 290.3, 294.2x, 294.8)	Age in 1996 (start of follow-up) White cases 71.5 (2.8), controls 71.2 (2.7); African Americans cases 70.9 (2.8), controls 71.4 (2.8)	Cox proportional hazards models with age as the time scale; cumulative risk of dementia	Included in crude model: age, sex, education<br><br>Fully adjusted model: further adjusted for midlife vascular risk factors (BMI and high blood pressure) and late-life vascular risk factors (stroke and diabetes)	Place of birth in high infant mortality rate areas (IMR; in quartiles)	White:<br>Q1 (lowest IMR) reference<br>Q2: HR 1.03 95%CI 0.89-1.19<br>Q3: HR 1.20 95%CI 1.00-1.45<br>Q4 (highest IMR): HR 1.06, 95%CI = 0.85-1.32<br><br>African-American:<br>Q1 (lowest IMR) reference<br>Q2: HR 1.03 95%CI 0.83-1.28<br>Q3: HR 0.92 95%CI 0.64-1.33<br>Q4 (Highest IMR) : HR 1.35 95%CI 0.97-1.88<br><br>Inside vs outside highest quartile IMR state	White:<br>Q1 (lowest IMR) reference<br>Q2: HR 1.03 95%CI 0.89-1.18<br>Q3: HR 1.20 95%CI 1.00-1.45<br>Q4 (highest IMR): HR 1.07 95%CI 0.86-1.34<br><br>African-American:<br>Q1 (lowest IMR) reference<br>Q2: HR 1.08, 95%CI 0.87-1.35<br>Q3: HR 0.96, 95%CI 0.67-1.40<br>Q4 (highest IMR) : HR 1.44 95%CI 1.03-2.01<br><br>Inside vs outside highest quartile IMR state	-All participants migrated to California

								White:<br>HR 1.01, 95%CI 0.84-1.22<br>African-American:<br>HR = 1.35, 95%CI = 0.99-1.84	White:<br>Inside: HR 1.03, 95%CI 0.85-1.24<br>African-American:<br>Inside: HR 1.40, 95%CI 1.02-1.91	
Glymour	86	All cause dementia or Alzheimer's disease as a cause of death (death records)	Mortality records were obtained from the National Center for Health Statistics multiple cause of death files. AD (ICD-10 G30), all cause dementia including AD (F00-F03, G30)	65-89 (in the year 2000)	Mortality rates; logistic regression	Included in crude model: Age, age-squared and sex<br><br>Fully-adjusted model: additionally adjusted for adult stroke belt residence	Place of birth (Stroke belt vs outside stroke belt)	All-cause dementia:<br>White:<br>OR 1.23 95%CI 1.21-1.25<br><br>African-American:<br>OR 1.25 95%CI 1.20-1.30<br><br>AD:<br>White:<br>OR 1.30 95%CI 1.27-1.33<br><br>African-American:<br>OR 1.28 95%CI 1.19-1.36<br><br>*(subgroup results in table C)*	All-cause dementia:<br>White:<br>OR 1.19 95%CI 1.16-1.22<br><br>African-American:<br>1.29 95%CI 1.24-1.35<br><br>AD:<br>Patterns nearly identical to those for all-cause dementia	-Limited reporting<br>-Cause of death on death certificates is not always accurate
Guaita	92	Dementia and dementia subtypes (Alzheimer's disease and Vascular dementia)	Geriatrician used DSM IV-TR criteria for dementia<br><br>AD: NINCDS-ADRDA criteria for probable, possible and definite diagnoses<br>Vascular dementia (VD): NINDS-AIREN criteria	71.69 (70-74)	Pearson's chi-squared test/ fisher's exact test, likelihood ratio test, logistic regression	Included in fully adjusted model: gender, birth year, marital status, primary lifetime occupation, years of education	Place of birth (location in Italy)	Crude prevalence<br>Dementia<br>North-western Italy: 2.3 95%CI 1.5-3.5<br>North-eastern Italy: 1.4 95%CI 0.2-4.9<br>Central Italy: 5.0 95%CI 0.1-24.9<br>Southern Italy: 9.4 95%CI 4.6-16.7<br>Italian islands: 6.3 95%CI 1.7-15.2<br>p=0.001<br><br>AD<br>North-western Italy: 0.9 95%CI 0.4-1.8<br>North-eastern Italy: 0.0 95%CI 0.0-2.5<br>Central Italy: 5.0 95%CI 0.1-24.9<br>Southern Italy: 1.9 95%CI 0.2-6.6<br>Italian islands: 4.7 95%CI 1.0-13.1	Mutually adjusted prevalence<br>Dementia<br>North-western Italy: 2.2 95%CI 1.4-3.4<br>North-eastern Italy: 1.1 95%CI 0.3-4.6<br>Central Italy: 4.9 95%CI 0.7-29.0<br>Southern Italy: 2.2 95%CI 1.4-3.4<br>Italian islands: 5.4 95%CI 2.1-15.4<br>p>0.900<br><br>Southern Italy vs north-western Italy OR 4.06 95%CI 1.76-9.34 p=0.001<br><br>AD<br>North-western Italy: 0.6 95%CI 0.2-1.4<br>North-eastern Italy: -<br>Central Italy: 3.7 95%CI 0.4-26.1	-Low number of participants born in certain subareas

								p=0.017<br><br>VD<br>North-western Italy: 1.1 95%CI 0.5-1.9<br>North-eastern Italy: 0.7 95%CI 0.0-3.8<br>Central Italy: 0.0 95%CI 0.0-16.8<br>Southern Italy: 5.7 95%CI 2.1-11.9<br>Italian islands: 1.6 95%CI 0.0-8.4<br>p=0.020	Southern Italy: 0.8 95%CI 0.2-4.2<br>Italian islands: 2.4 95%CI 0.6-9.0<br>p=0.624<br><br>VD<br>North-western Italy: 1.6 95%CI 0.3-1.5<br>North-eastern Italy: 0.4 95%CI 0.1-3.2<br>Central Italy: -<br>Southern Italy: 3.7 95%CI 1.4-9.5<br>Italian islands: 1.1 95%CI 0.1-8.3<br>p>0.900<br><br>Southern Italy vs north-western Italy OR 6.36 95%CI 1.98-20.43 p=0.002	
Scazufca	84	Dementia	Harmonized one-phased dementia diagnostic procedure developed by the 10/66 Dementia Research Group and validated for use in population-based studies of LAMIC; diagnosis following DSM-IV criteria	72.2 (6.3)	Logistic regression	Included in crude model: age and gender<br><br>Included in fully adjusted model: literacy, head circumference, leg length, occupation, income, smoking, diabetes, hypertension, age and gender	Place of birth (city, town or rural)	City: prevalence = 2.7; reference<br>Town: prevalence = 3.9; OR 1.78 95%CI 0.68-4.66<br>Rural: prevalence = 5.8; OR 2.22 95%CI 0.94-5.24<br><br>p-value for linear trend=0.04 (likelihood ratio test)	City: reference<br>Town: OR 1.22 95%CI 0.45-3.32<br>Rural: OR 1.23 95%CI 0.50-3.05<br><br>p-value for linear trend=0.70	-
Topping	87	Mortality from all-cause dementia or Alzheimer's disease	Mortality status was obtained from yearly linkage with the Master Death Files of the Social Security Administration. Cause of death information was searched in the National Death Index and based on ICD codes. Follow-up from 1995 to 2011.	63.32 (4.53; 55-72) at baseline	Logistic regression	Crude model: sex, age and age squared<br><br>Fully adjusted model: additionally adjusted for educational attainment and self-rated health<br><br>All models were modelled with and without adult stroke belt residence included and separately	Place of birth (stroke belt)	All cause dementia mortality<br>White population<br>OR 1.13 95%CI 1.07-1.20<br>Non-white population<br>OR 1.24 95%CI 1.01-1.52<br><br>Alzheimer's disease<br>White population<br>OR 1.21 95%CI 1.04-1.41<br>Non-white population<br>OR 0.90 95%CI 0.46-1.76	All cause dementia mortality<br>White population<br>OR 1.08 95%CI 1.02-1.15<br>Non-white population<br>OR 1.14 95%CI 0.92-1.41<br><br>Alzheimer's disease<br>White population<br>OR 1.20 95%CI 1.02-1.40<br>Non-white population<br>OR 0.91 95%CI 0.46-1.79	-

						for the white and non-white population		*Including adult stroke belt residence:*<br><br>All cause dementia mortality<br>White population<br>OR 1.03 95%CI 0.96-1.12<br>Non-white population<br>OR 1.12 95%CI 0.88-1.45<br><br>Alzheimer's disease<br>White population<br>OR 1.15 95%CI 0.94-1.40<br>Non-white population<br>OR 0.85 95%CI 0.38-1.90	*Including adult stroke belt residence:*<br><br>All cause dementia mortality<br>White population<br>OR 0.97 95%CI 0.90-1.05<br>Non-white population<br>OR 1.05 95%CI 0.81-1.35<br><br>Alzheimer's disease<br>White population<br>OR 1.16 95%CI 0.95-1.42<br>Non-white population<br>OR 0.85 95%CI 0.38-1.92	
Wilson	91	Alzheimer's disease	NINCDS-ADRDA criteria	75.0 (7.0)	Proportional hazards model	Included in crude model: age and sex<br><br>Fully adjusted model: additionally adjusted for education	Place of birth (county SES level)	RR of incident AD associated with county socioeconomic level (per 1-unit increase in SES)<br><br>RR 1.36 95%CI 0.94-1.97	RR 1.35, 95%CI 0.93-1.96	-Population not representative

Information and results from studies regarding place of birth.

*Reporting was not complete or not clear.

AD=Alzheimer's disease

DSM=Diagnostic and Statistical Manual of Mental Disorders

ICD=International Classification of Diseases.

NINCDS-ADRDA=National Institute of Neurological and Communicative Disorders and Stroke and the Alzheimer's Disease and Related Disorders Association (now known as the Alzheimer's Association)

NINDS-AIREN=National Institute of Neurological Disorders and Stroke and the Association Internationale pour la Recherche et l'Enseignement en Neurosciences

NS=Not statistically significant

SES=Socioeconomic status

Other factors

Author	Ref.	Outcome: Type	Outcome: Measurement/criteria	Outcome: Age; mean (SD; range)	Statistical test	Covariates accounted for in model or by matching	Exposure	Results (by exposure): Unadjusted/crude model	Results (by exposure): Fully adjusted model	Key limitations
**Other factors**										
Case-control studies										
Jiang	97	Dementia	Self-report of significant memory impairment or dementia. In subjects with dementia, there was an additional query about the type. For some patients, information was provided by caregivers.	Men: 82.8 (1.1; 65-97) Women: 84.5 (1.0; 65-104)	Two-way ANOVA and Turkey post hoc test for pairwise comparisons.	Sex was accounted for in the models	Right hand second to fourth finger length ratio as an indication of prenatal sex hormone exposure (2D:4D)	Men: p=0.57<br><br>Women: p=0.006<br>Right hand 2D:4D higher in those with dementia		-Self-reported dementia measure<br>-Indirect measure of exposure
Vladeanu	96	Alzheimer's disease	Diagnosed according to the NINCDS-ADRDA and DSM-IV-TR criteria by a multidisciplinary team of a consultant psychiatrist, clinical psychologist and dementia nurse specialist	Cases 78.3 controls 75.6	MANOVA and 2x2 independent measures analyses of variance	Matched on: age and years of education	The second to fourth finger length ratio as an indication of prenatal sex hormone exposure	Group main effect/MANOVA: NS<br><br>Mean 2D:4D ratio<br>Men<br>Left hand: Cases: 1.03<br>Controls: 0.96 (0.02)<br>p<0.001<br><br>Women<br>Left hand: Cases: 0.97 (0.03) vs Controls: 1.03 (0.04)<br>p=0.004<br><br>Similar results for mean (left and right hand) ratios. No significant results for right hand ratios	-	-Indirect measure of exposure<br>-Small sample
Cohort studies										
Cocoros	93	Dementia of any type and specific types of	Registry data, diagnostic ICD codes, eight and tenth revisions	Followed from age 62 until max	Poisson regression; incidence rate ratios (IRR)	Included in crude model: sex	Prenatal 1918 influenza pandemic exposure	All ages:<br>IRR 1.01 95%CI 0.99-1.04<br><br>Per type	-	-Indirect measure of exposure<br>-Healthy survivor bias

		dementia (AD, vascular dementia, other dementia)	Danish National Patient Registry (DNPR) and the Psychiatric Central Research Registry (PCRR) between January 1 1977 and November 30 2013	their 92th birthday		They Included participants born before and conceived after the influenza pandemic to account for age		AD IRR 0.97 (0.93-1.01)<br>vascular dementia: IRR 1.04 95%CI 0.98-1.10<br>other dementia: IRR 1.03 95%CI 1.00-1.06<br><br>*(subgroup results in table C)*		
Kang	94	Dementia	The Clinical Dementia Rating CDR scale was administered in all subjects; dementia was diagnosed according to the DSM-5	Pre-famine: 56.48(1.23), famine: 53.42(1.13), post-famine: 50.50(1.20)	Chi-squared test or Fisher's exact test to analyze the differences among the groups	-	Prenatal famine exposure	Famine vs post-famine: 0.72% vs 0.27%, p=0.0331<br>Famine vs pre-famine: 0.72% vs 0.67%, p=0.8733<br><br>*(subgroup results in table C)*	-	-No correction for age<br>-Participants were relatively young at outcome assessment
Lenz	95	Dementia or Alzheimer's disease as cause of death (national normalized death rates)	National point estimates for deaths (adjusted for the population size) in 2012, from the Global Health Estimates summary tables were used (WHO 2014)	Not reported and not relevant; looked at death rate irrespective of age	Pearson correlation; not normally distributed data were transformed into rankit normal scores<br><br>They adjusted p-values for multiple hypothesis testing with the false discovery rate (FDR) procedure	-	The second to fourth finger length ratio as an indication of prenatal sex hormone exposure (2D:4D)	Cross-national correlations for men:<br>r= -0.59<br>p= 0.0032<br>p(FDR)= 0.0361<br><br>For women the results showed the same correlations as in men (not shown)	-	-Comparison across nations not across individuals, very crude analysis<br>-Self-reported measurements from different population than population at risk for dying of dementia.<br>-Not accounting for other causes/confounding
Luo	98	Dementia (onset >60 years)	Clinical diagnosis by the research team, from the Swedish National Register or from the prescribed drug register as a proxy for dementia diagnosis. Using DSM-III-R or DSM-IV criteria or ICD codes	>60	Logistic regression and cox proportional hazard regression by sex	Crude model: birth year<br><br>Fully adjusted model: birth year, education, exercise, vascular risk, and postnatal hormone exposure	Presumed differential prenatal hormone exposure by comparing same- and opposite- sex dizygotic twin pairs	Logistic regression<br>Opposite-sex compared to same-sex<br><br>Women<br>OR 0.37 95%CI 0.25-0.54 p<0.0001<br>Men<br>OR 0.47 95%CI 0.31-0.70 p<0.0001<br><br>Cox regression<br>Women<br>HR 0.83 95%CI 0.65-1.06 p=0.141	Similar results<br><br>Interaction between twin type and APOE e4 was NS	-All participants were twins (less generalizable)<br>-Indirect measure of prenatal hormone environment

	Men<br>HR 0.96 95%CI 0.74-1.25<br>p=0.751<br><br>Diagnosis after 70<br>Women<br>HR 0.64 95%CI 0.48-0.87<br>p=0.004<br>Men NS<br><br>Diagnosis after 80<br>Women<br>HR 0.38 95%CI 0.21-0.68<br>p=0.001<br>Men NS

Information and results from studies regarding other factors.

AD=Alzheimer's disease

CDR=Clinical Dementia Rating Scale

DSM=Diagnostic and Statistical Manual of Mental Disorders

ICD=International Classification of Diseases.

NINCDS-ADRDA=National Institute of Neurological and Communicative Disorders and Stroke and the Alzheimer's Disease and Related Disorders Association (now known as the Alzheimer's Association)

NS=Not significant

Birth characteristics

Author	Ref.	Outcome: Type	Outcome: Measurement/criteria	Outcome: Age; mean (SD; range)	Statistical test	Covariates accounted for in model or by matching	Exposure	Results (by exposure): Unadjusted/crude model	Results (by exposure): Fully adjusted model	Key limitations
**Birth size**										
Cohort studies										
Matshusima	99	Dementia	Self-reported; ever diagnosed with dementia	Cases 65.0 (6.7), controls 63.3 (6.6) at outcome assessment	Regression with linear probability model (binary outcome)	Included in adjusted model: sex, birth year, birth before the end of WWII, residential municipality, educational attainment, household SES during childhood, smoking and owning a home with land in adulthood	Low birth weight (including premature birth)	-	0.024 (0.013)<br>R-squared: 0.04<br>NS	-Self-reported exposure and outcome<br>-Limited reporting<br>-Multiple testing<br>-Limited number of cases/small sample size
Mosing	65	Dementia (clinical diagnosis or cause of death)	Register-based dementia diagnoses as well as date of first diagnosis were derived from the NPR and CDR. ICD 7-10 codes for AD, vascular dementia and other dementia	74.3 (2.6; 55-88)	Cox proportional hazard analysis; corrected for correlation within twin pairs	Included in fully-adjusted model: sex, year of birth (10 year interval), parity, age of mother, birth SES, education level	Birth weight	BW (100g): HR=0.98, 95%CI=0.97-1.00, p=0.016<br>LBW: HR=1.19, 95%CI=1.04-1.36, p=0.011<br>BWGA: HR=0.92, 95%CI=0.86-0.99, p=0.017<br>SGA: HR=1.19, 95%CI=0.81-1.76, p=0.380	BW (100g) 0.98 (0.97-0.99) p=0.004<br>LBW 1.22 (1.07-1.40) p=0.004<br>BWGA 0.91 (0.85-0.98) p=0.008<br>SGA 1.19 (0.80-1.76) p=0.420	-Registry data might miss a lot of dementia diagnosis<br>-Only twins were included (population not representative)
							Head circumference at birth	HC (mm): HR 1.00, 95%CI 0.99-1.00, p=0.304<br>HCGA 0.97 95%CI 0.90—1.04, p=0.401<br>SHCGA 1.67 95%CI 1.15-2.41 p=0.007	HC (mm) 1.00 (0.99-1.00) 0.220<br>HCGA 0.97 (0.90-1.04) 0.415<br>SHCGA 1.65 (1.14-2.39) 0.008	
							Birth length	BL (cm): HR=0.98, 95%CI=0.96-1.01, p=0.136<br>BLGA: HR=0.96, 95%CI=0.89-1.02, p=0.195<br>SBLGA: HR=1.40, 95%CI=0.99-1.98, p=0.058	BL (cm) 0.98 (0.95-1.00) 0.073<br>BLGA 0.95 (0.89-1.02) 0.167<br>SBLGA 1.40 (0.98-1.98) 0.062	

							Gestational age	GA (weeks) 0.99 (0.96-1.02) p=0.461<br>Preterm 0.96 (0.82-1.13) p=0.652	GA (weeks) 0.99 (0.96-1.02) 0.396<br>Preterm 0.97 (0.82-1.14) 0.729	
							Birth SES	NS*<br><br>*(subgroup results in table C)*		
Syddall	100	Alzheimer's disease and dementia (as cause of death)	Death certificate according to ICD-9; AD: 331.0, dementia: 290 and 297	All ages (death certificates); separate analysis by age 65 and 75	Cox proportional hazards model; hazard ratio presented per standard deviation increase in birth weight	Birth year was included in all cox proportional hazard models	Birth weight	Only sex-specific results were presented<br><br>*(subgroup results in table C)*	-	-Limited reporting<br>-Only presented sex-specific analysis<br>-Low number of dementia cases

Information and results from studies regarding birth characteristics.

*Reporting was not complete or not clear.

AD=Alzheimer's disease

CDR=Clinical Dementia Rating Scale

ICD=International Classification of Diseases.

NPR=National patient registry

NS=Not significant

SES=Socioeconomic status

Table C – Subgroup results

Parental age and birth order

Author	Ref.	Exposure	Sex-specific analysis		Other sub-group analyses	
			Description and statistical test	Results (by exposure)	Description and statistical test	Results (by exposure)
**Parental Age and birth order**						
Case-control studies						
Bertram	45	Maternal age	-	-	Independent student's t-test;<br>Low major AD gene probability (L) vs control	(L) mean(SD) 29.4(6.6) vs 28.0(6.8) p=NS<br>(H) mean(SD) 29.1(5.7) vs 28.0(6.8) P=NS
		Paternal age			High major AD gene probability (H) vs control	(L) mean(SD) 35.7 (8.1) vs 32.6 (6.8) p=0.04<br>(H) mean(SD) 31.3(6.9) vs 32.6(6.8) p=NS
Clarnette	46	Maternal age	-	-	Means were reported for possible AD, probable AD and possible/probable AD with a family history of AD	Controls (spouses) mean 27.9 SE 0.57<br>Possible AD mean 28.1 SE 1.01<br>Probable AD mean 30.5 SE 0.92<br>AD with family history of AD mean 29.7 SE 1.25
		Paternal age				Controls (spouses) mean 31.1 SE 0.73<br>Possible AD mean 31.9 SE 1.10<br>Probable AD mean 34.3 SE 1.17<br>AD with family history of AD mean 33.4 SE 1.35
Corkin	42	Maternal age	-	-	Means reported for AD onset <65 or ≥65 years	Cases <65 years 27.5 (19-44)<br>≥65 years 28.2 (18-35)<br>Controls 27.4 (17-40)
		Paternal age				<65 years 30.4 (21.50)<br>≥65 years 31.2 (25-42)<br>Controls 29.4 (18-39)
English	47	Maternal age	-	-	Chi-square test for trend; 95% CIs for odds ratios calculated by Cornfields method; early (<65) and late (65+) onset AD cases	Distribution of early onset (<65) and late onset cases (65+) were very similar; values not reported

Farrer 1991	33	Maternal age	-	-	Paired comparison t-test and conditional logistic regression (5- year age interval)<br><br>Early onset (<67.2) vs late onset (>67.2)<br>Family history (familial AD) vs no family history of AD (sporadic AD)<br><br>Parental and maternal age were included in one model*	Late onset (>67.2), mean maternal age, cases 27.0 controls 28.3, p=0.035<br>Late onset, sporadic AD, mean maternal age, cases 26.5, controls 28.1, p=0.050<br>Family history: no difference in mean maternal age<br>Early onset (<67.2) OR 1.07 95%CI 0.83-1.37<br>Late onset OR 1.01 95%CI 0.82-1.25<br>Early onset, familial AD, OR 1.30 95%CI 0.82-2.06<br>Early onset, sporadic AD, OR 0.97 95%CI 0.72-1.31<br>Late onset, familial AD, OR 1.18 95%CI 0.82-1.70<br>Late onset, sporadic AD, OR 0.93 95%CI 0.71-1.21
		Paternal age				Late onset (>67.2), mean paternal age, cases 30.2 controls 31.5, p=0.003<br>Late onset, sporadic AD, mean paternal age, cases 29.8 controls 32.0, p=0.019<br>Family history: no difference in mean paternal age<br>Early onset (<67.2) OR 1.02 95%CI 0.81-1.29<br>Late onset OR 0.80 95%CI 0.67-0.96 p=0.019<br>Early onset, familial AD, OR 0.89 95%CI 0.55-1.42<br>Early onset, sporadic AD, OR 1.07 95%CI 0.82-1.40<br>Late onset, familial AD, OR 0.71 95%CI 0.51-0.99 p=0.045<br>Late onset, sporadic AD, OR 0.85 95%CI 0.68-1.06
Farrer 1997	59	Maternal age	-	-	Conditional logistic regression; grouped by combination of APOE genotype and two parental age categories (old vs young defined by de median of 28 and 31 for maternal and paternal age respectively	E3/e3 OR 1.13 95%CI 0.82-1.56<br>E2/e3 OR 2.26 95%CI 0.95-5.33<br>E2/e4 OR 1.38 95%CI 0.36-5.25<br>E3/e4 OR 1.62 95%CI 1.14-2.30<br>E4/e4 OR 2.06 95%CI 0.72-5.88<br>Least square mean, E3/e4 cases 29.5 controls 28.0 p=0.007 (adjusted for gender, age and year of birth)
		Paternal age				E3/e3 OR 1.34 95%CI 0.98-1.84<br>E2/e3 OR 1.16 95%CI 0.49-2.68<br>E2/e4 OR 0.54 95%CI 0.12-2.32<br>E3/e4 OR 1.79 95%CI 1.26-2.54<br>E4/e4 OR 1.26 95%CI 0.47-3.41

						Least square mean, E3/e4 cases 33.3 controls 30.8 p<0.0001 (adjusted for gender, age and year of birth)
Hofman	50	Maternal age	-	-	Conditional logistic regression (matched design) for sporadic AD cases (without a family history of dementia)	≤24 reference<br>25-29 RR 0.8 95%CI 0.4-1.7<br>30-34 RR 0.9 95%CI 0.4-1.9<br>35-39 RR 0.7 95%CI 0.3-1.9<br>40+ RR 0.9 95%CI 0.3-2.8<br>Difference in mean maternal age were similar for individuals with or without a family history of dementia
		Paternal age				≤24 reference<br>25-29 RR 1.1 95%CI 0.4-2.8<br>30-34 RR 1.0 95%CI 0.4-2.6<br>35-39 RR 0.7 95%CI 0.2-2.2<br>40+ RR 0.8 95%CI 0.2-2.5<br>Difference in mean paternal age were similar for individuals with or without a family history of dementia
		Birth order				NS for individuals with an without a family history of dementia
Whalley 1995	34	Maternal age	Conditional logistic regression; matched on: sex, birth registration district, date of birth (same year), father's occupational group	Women: p=0.20<br>Men: OR 1.13 95%CI 1.06-1.20<br>Both in a model with paternal age, for women effect was significant when testing maternal age separately	-	
		Paternal age	Included in adjusted model: parental and maternal age in one model	Women: p=0.19<br>Men: OR 1.13 95%CI 1.06-1.20 p<0.001<br>Both in a model with maternal age, for women effect was significant when testing maternal age separately		
Cohort studies						
Lahti	67	Maternal grand multiparity	Cox proportional hazards models, sex specific results<br><br>Model 1: Adjusted for year of birth (1934-1938 or 1939-1944), mother's and father's highest attained occupational statuses, birth weight, maternal body<br>mass index at childbirth	Women<br>Model 1 HR 0.73 95%CI 0.10–5.40, p=0.76<br>Model 2 HR 0.79 95%CI 0.10–6.05, p=0.82<br><br>Men<br>Model 1 HR 1.07 95%CI 0.25–4.45, p=0.93<br>Model 2 HR 1.32 95%CI 0.31–5.62, p=0.70	-	-

	Model 2: Adjusted for year of birth (1934-1938 or 1939-1944), mother's and father's highest attained occupational statuses, birth weight, maternal body mass index at childbirth, and maternal age at childbirth.

Subgroup results from studies regarding parental age and/or birth order.

AD=Alzheimer's disease

Season of birth

Author	Ref.	Exposure	Sex-specific analysis		Other sub-group analyses	
			Description and statistical test	Results (by exposure)	Description and statistical test	Results (by exposure)
**Season of birth**						
Case-control studies						
Dysken	68	Season of birth (by quarter)	Chi-squared analysis: observed vs expected births, individual quarter vs remaining quarters (yates correction); Edwards' procedure for estimating cyclic trends	No sex effects where observed in the chi-squared analyses.<br><br>Edwards' test for peak quarter was significant for women (x^2=7.0, df=2, p<0.05), peak period occurring early in the first quarter; no peak was established for AD men	Chi-squared analysis: observed vs expected births, individual quarter vs remaining quarters (yates correction); family history of AD	Family history chi-squared: 1.14 (NS)<br>No family history chi-squared: 1.93 (NS)<br>Individual quarter vs remaining quarters: NS
Henderson	69	Season of birth (by quarter)	-	-	Chi-squared test and Roger's methods for cyclic trends<br><br>Family history of dementia<br><br>Early (<75 years) or late-onset cases (≥75 years)<br><br>Southern hemisphere births	Per quarter<br>'Familial' cases<br>Cases vs controls: chi-squared: 4.22 p=0.24<br>Cases vs general population: chi-squared: 6.88 p=0.08<br>Roger's test: chi-square: 4.46 p=0.11<br><br>'Sporadic' cases<br>Cases vs controls: chi-squared: 1.91 p=0.59<br>Cases vs general population: chi-squared: 0.95 p=0.81<br>Roger's test: chi-square: 1.34 p=0.51<br><br>Early-onset cases (<75 years)<br>Cases vs controls: chi-squared: 2.54 p=0.67<br>Cases vs general population: chi-squared: 2.72 p=0.44<br>Roger's test: chi-squared: 1.95 p=0.38<br><br>Early-onset cases (<75 years)<br>Cases vs controls: chi-squared: 0.95 p=0.81<br>Cases vs general population: chi-squared: 2.86 p=0.41<br>Roger's test: chi-squared: 0.30 p=0.86<br><br>All aforementioned results were additionally shown for Southern hemisphere births only; Results were not statistically significant expect for

						the Roger’s test in ‘familial’ cases: 5.91 p=0.05; proportionally fewer births in the southern summer months of January and February
Philpot	73	Season of birth (per quarter)	-	-	Chi-squared test for observed vs expected births and Roger's method for testing cyclic trends and Edwards' method for determining the peak quarter of birth<br><br>With and without a family history of dementia for AD cases	With and without family history for AD patients<br>Family history: Chi-squared (df=3): 3.04 p>0.1<br>Without a family history: Chi-squared: 14.85 p<0.01<br><br>AD patients without family history of dementia, significance of cyclical trend (Roger's method) p<0.05; peak quarter (Edward's method) early 1st.<br><br>AD patients without family history of dementia, significance of cyclical trend p<0.4, peak quarter early 3rd<br><br>General population 1971 1% sample; cyclical trend P<0.0005; peak quarter May; as shown for comparison
Ptok 2001	70	Season of birth (quarterly birth rates)	-	-	Chi-squared test; comorbid AD and depression; ApoE4 negative or positive for AD and comorbid AD and depression	Comorbid AD and depression vs controls, chi-squared: 0.65 p=0.89<br>AD and co-morbid AD and depression - ApoE4 negative, chi-squared: 4.14 p=0.25<br>AD and co-morbid AD and depression - ApoE4 positive, chi-squared: 1.36 p=0.72
Vitiello	71	Season of birth (quarterly birth rates)	-	-	Chi-squared test; Edwards method for cyclic trend	Patients with AD with negative family history of AD compared to controls.<br>Chi-squared: 4.0 (df=3) p=0.26
Ding	78	Season of birth	-	-	Logistic regression; residence (urban or rural), geographic region (north or south); adjusted for: age, gender, education and annual household income per capita	Urban residence:<br>Spring (Mar, Apr, May) OR 0.64 95%CI 0.49-0.84 p=0.002<br>Summer Reference<br>Autumn (Sep, Oct, Nov) OR 0.80 95%CI 0.62-1.03 p=0.084<br>Winter (Dec, Jan, Feb) OR 0.63 95%CI 0.49-0.83 p=0.001<br><br>Rural residence:<br>Spring (Mar, Apr, May) OR 0.98 95%CI 0.80-1.21 p=0.868<br>Summer Reference

						Autumn (Sep, Oct, Nov) OR 0.95 95%CI 0.77-1.17 p=0.644<br>Winter (Dec, Jan, Feb) OR 0.84 95%CI 0.67-1.04 p=0.109<br><br>North:<br>Spring (Mar, Apr, May) OR 0.82 95%CI 0.64-1.05 p=0.117<br>Summer Reference<br>Autumn (Sep, Oct, Nov) OR 0.79 95%CI 0.62-1.01 p=0.063<br>Winter (Dec, Jan, Feb) OR 0.66 95%CI 0.51-0.86 p=0.002<br><br>South:<br>Spring (Mar, Apr, May) OR 0.88 95%CI 0.70-1.10 p=0.259<br>Summer Reference<br>Autumn (Sep, Oct, Nov) OR 0.97 95%CI 0.78-1.20 p=0.753<br>Winter (Dec, Jan, Feb) OR 0.83 95%CI 0.67-1.03 p=0.097
Cohort studies						
Hsu	81	Month of birth	Walter & Elwood’s seasonality test	Performing the analysis in men alone<br>P <0.001	Walter & Elwood’s seasonality test	Diagnosis occurred at least 3 times<br>P <0.001<br><br>Comorbidities ≤ 1<br>P <0.001<br><br>Younger onset (below average)<br>P <0.001<br><br>Higher urbanization<br>P <0.001<br><br>Income lower than average<br>P <0.001

Subgroup results from studies regarding season of birth.

AD=Alzheimer’s disease

Place of birth

Author	Ref.	Exposure	Sex-specific analysis		Other sub-group analyses	
			Description and statistical test	Results (by exposure)	Description and statistical test	Results (by exposure)
Place of birth						
Case-control studies						
Forster	48	Place of birth (Aluminium concentration in drinking water)	-	-	OR's calculated with McNemar's test (taking matching into account)<br><br>With and without a familial case of AD	No differences compared to overall results
Glymour	86	Place of birth (Stroke belt vs outside stroke belt)	-	-	Mortality rates; logistic regression<br>Crude model: adjusted for age, age-squared and sex<br><br>Stratified by adult stroke belt residence	Adult stroke belt residence (2000)<br>White<br>All cause dementia OR 1.22 95%CI 1.20-1.25<br>Alzheimer's disease OR 1.33 95%CI 1.27-1.35<br><br>African American<br>All cause dementia OR 1.21 95%CI 1.15-1.26<br>Alzheimer's disease OR 1.27 95%CI 1.17-1.37<br><br>No adult stroke belt residence (2000)<br>White<br>All cause dementia OR 1.25 95%CI 1.22-1.29<br>Alzheimer's disease OR 1.30 95%CI 1.24-1.36<br><br>African American<br>All cause dementia OR 1.29 95%CI 1.24-1.36<br>Alzheimer's disease OR 1.27 95%CI 1.17-1.37
Jean	82	Place of birth (rural and urban vs reference population)	Rural<br>Men OR=0.83<br>Women OR=1.79*<br><br>Urban<br>Men OR=0.38*<br>Women OR=1.33*<br><br>Total<br>Men OR=0.54*<br>Women OR=1.49*<br><br>*statistically significant	-	Analysis were repeated without kin pairs at a three generation depth	Rural<br>Men OR=0.67*<br>Women OR=1.81*<br><br>Urban<br>Men OR=0.39*<br>Women OR=1.41*<br><br>Total<br>Men OR=0.48*<br>Women OR=1.55*<br><br>*statistically significant

Cohort studies						
Gilsanz 2017	85	Place of birth (in high stroke mortality state)	-	-	Cox proportional hazard models with age as the time scale; cumulative risk of dementia<br><br>Crude model: adjusted for age and sex<br>Fully adjusted model: adjusted for age, sex, education, midlife (BMI, smoking duration, hypertension) and late life (diabetes, hypertension, heart failure, acute myocardial infarction and stroke) cardiovascular risk factors	Crude model:<br>Black participants HR 1.13 95%CI 0.94-1.35<br>Non-black participants HR 1.46 95%CI 1.23-1.74<br><br>Fully adjusted model:<br>Black participants HR 1.14 95% CI 0.95-1.36<br>Non-black participants HR 1.42 95% CI 1.19-1.69

Subgroup results from studies regarding place of birth.

AD=Alzheimer's disease

Other factors

Author	Ref.	Exposure	Sex-specific analysis		Other sub-group analyses	
			Description and statistical test	Results (by exposure)	Description and statistical test	Results (by exposure)
Other factors						
Cohort studies						
Cocoros	93	Prenatal 1918 influenza pandemic exposure	Poisson regression; incidence rate ratios (IRR); sex-specific analysis	Women:<br>All ages: 1.02 95%CI 0.99-1.05<br>62-72: 0.96 95%CI 0.86-1.07<br>72-82: 1.05 95%CI 1.00-1.11<br>82-92: 1.01 95%CI 0.97-1.04<br><br>AD 1.01 95%CI 0.96-1.06<br>Vascular dementia 1.04 95%CI 0.96-1.12<br>Other dementia 1.02 95%CI 0.98-1.06<br><br>Men:<br>All ages: 1.01 95%CI 0.97-1.05<br>62-72: 0.82 95%CI 0.74-0.92<br>72-82: 1.07 95%CI 1.01-1.13<br>82-92: 1.00 95%CI 0.95-1.06<br><br>AD 0.91 95%CI 0.85-0.97<br>Vascular dementia 1.04 95%CI 0.95-1.14<br>Other dementia 1.06 95%CI 1.01-1.11	Poisson regression; incidence rate ratios (IRR); included in the model: sex.<br><br>Sensitivity analysis<br>-By age<br>-Inpatient only<br>-restricted to those exposed during the first trimester compared to those born before June 1918 only	By age:<br>62-72: IRR 0.89 95%CI 0.82-0.96<br>72-82: IRR 1.06 95%CI 1.02-1.10<br>>82: IRR 1.01 95%CI 0.98-1.04<br><br>Inpatient only<br>Generally similar results; some significant results, however, point estimates were modest<br><br>First trimester compared to born before June 1918; generally similar results; some significant results, however, point estimates were modest<br><br>Any dementia 1.02 95%CI 0.96-1.08
Kang	94	Prenatal famine exposure		There was no difference among men and women subgroups		

Subgroup results from studies regarding other factors.

AD=Alzheimer's disease

Birth characteristics

Author	Ref.	Exposure	Sex-specific analysis		Other sub-group analyses	
			Description and statistical test	Results (by exposure)	Description and statistical test	Results (by exposure)
Birth size						
Cohort studies						
Mosing	66	Birth weight (z-score)<br><br>Head circumference (z-score)			Within-pair analyses in monozygotic twins and in monozygotic (MZ) twins and same-sex dizygotic (DZ) twin pairs; conditional cox regression models with family identification as the stratum variable	MZ twins: 0.81 95%CI 0.53-1.24<br>MZ and same-sex DZ: 0.90 95%CI 0.71-1.15<br><br>MZ twins: 0.83 95%CI 0.53-1.29<br>MZ and same-sex DZ: 0.92 95%CI 0.71-1.21
Syddall	100	Birth weight	Cox proportional hazards model; hazard ratio presented per standard deviation increase in birth weight; birth year was included in the models	Men:<br>Dementia HR 1.63 95%CI 1.10-2.43 p=0.02 (13 deaths)<br>AD HR 0.60 95%CI 0.32-1.13 p=0.12 (8 deaths)<br><br>Women:<br>Dementia 0.66 (0.23-1.91) p=0.44 (3 deaths)<br>AD 3.29, 95%CI 1.67-6.48 p= 0.001 (5 deaths)<br>Results were consistent in an analysis of mortality by age 75 years and 65 years<br><br>Excluding women who weighed more than 4 kg at birth attenuated the associations of birth weight with mortality from AD	-	-

Subgroup results from studies regarding birth characteristics.

AD=Alzheimer's disease
