# Supplementary material for: Do prenatal factors shape the risk for dementia?: A systematic review of the epidemiological evidence for the prenatal origins of dementia
Source: Soc Psychiatry Psychiatr Epidemiol. 2023 Apr 8;60(5):977–91. doi: 10.1007/s00127-023-02471-7 (PMC12119685; doi:10.1007/s00127-023-02471-7)
Supplement: Supplementary file 5 — Supplementary file5 (PDF 326 KB) [file 127_2023_2471_MOESM5_ESM.pdf]

Online Resource 5: Text regarding the subgroup results

Social Psychiatry and Psychiatric Epidemiology

Do prenatal factors shape the risk for dementia?: A systematic review of the epidemiological evidence for the prenatal origins of dementia

Aline Marileen Wiegersma*, Amber Boots, Miranda W. Langendam, Jacqueline Limpens, Susan D. Shenkin, Aniko Korosi, Tessa J. Roseboom, Susanne R. de Rooij

*Corresponding author: Amsterdam UMC location University of Amsterdam, Epidemiology and Data Science, Meibergdreef 9, Amsterdam, The Netherlands, e-mail: a.m.wiegersma@amsterdamumc.nl

Sub group results are also presented in Online Resource 4.

Parental age and birth order

Nine studies performed subgroup analyses. Bertram et al. performed an analysis separately for individuals with high or low major AD gene probability. The results regarding maternal age were both non-significant, however, in the low major gene probability group cases had significantly higher paternal age compared to controls [1].

Three studies reported subgroup results regarding family history of AD, defined as having a first-degree relative with AD [2–4]. Clarnette et al. reported mean results separately for AD cases with a family history of AD, however, they did not report any statistical results and it was therefore unclear if there were any differences among these groups. The results of Hofman et al. for parental age and birth order remained non-significant for the group with and without a family history of AD [4]. Farrer et al. (1991) showed subgroup results for both family history of AD and early (<67.2 years) or late (>67.2 years) onset of AD. The mean maternal age was significantly lower for both late onset cases and early onset sporadic AD cases compared to controls, however, results from conditional logistic analysis per 5-year maternal age increase were not statistically significant for these and other subgroups [3]. Furthermore, mean paternal age was also lower in both late onset cases and early onset sporadic AD cases. Logistic regression analysis showed lower AD risk for the late onset group and the late onset familial subgroup with higher paternal age [3].

Additionally, two other studies reported subgroup results for early (<65) and late onset (≥65) AD [5,6]. Corkin et al. only reported means and English et al. reported similar results for early and late onset AD.

Farrer et al. (1997) examined their results per APOE genotype group by two parental age categories (old vs young defined by a median of 28 and 31 for maternal and paternal age respectively). Persons with the high risk APOE genotype E3/e4 had higher risk of AD if their mother or father was older than the median maternal or paternal age at birth compared to individuals with the E3/e4 genotype with younger mothers and fathers [7].

Furthermore, Lahti et al. reported sex-specific results for being born as the sixth or later child and did not observe an association with dementia for either women or men [8]. Whalley et al. (1995) reported no significant associations in women between parental age and AD when adding maternal and paternal age to a single model, however, the associations were statistically significant when fitting these factors separately. In men, the risk of AD increased with higher paternal age, but decreased with higher maternal age when these factors were fitted in the same model.

Season of birth

Seven studies performed subgroup analyses. For Dysken et al., the chi-squared results remained non-significant in a sex-specific analysis, however, significantly more births were observed in the first quarter in women in a cyclic trend analysis without comparison to a control population [9].

Four studies included an analysis comparing those with or without a family history of dementia, for three studies the results remained non-significant in these groups [9–11]. For Philphot et al. the quarterly variation of births for AD cases was different than the expected births in individuals without a family history of dementia. Furthermore, a significant peak of births early in the first quarter was observed, not in line with the peak of births in the general population in May. Henderson et al. additionally examined subgroups with early (<75 years) or late onset (≥75) AD, but the results were not statistically significant in either group. Additionally, they repeated all analyses only including individuals born in the Southern hemisphere. These analyses remained non-significant, except for familial AD cases for whom proportionally fewer births in the southern summer months of January and February were observed with Roger's test [10].

Ptok et al. presented results for cases with comorbid AD and depression and ApoE4 positive or negative status. In these analyses, season of birth was not significantly different among comorbid AD and depression cases compare to controls. Furthermore, the results were also not significant considering ApoE4 status [12].

Ding et al. performed their analysis separately for individuals with rural or urban residence and by geographic region (north or south). For the urban residence group, winter and spring born individuals were significantly less likely to develop dementia compared to those born in summer [13]. For those living in the north of China, winter born individuals were less likely to develop dementia compared to those born in summer. No statistically significant differences were observed in the rural residence or southern groups [13].

Hsu et al. performed six sensitivity analyses. They performed their analyses: 1) in men alone; 2) only including patients with no more than one comorbidity; 3) in persons with earlier than average onset; 4) in persons living in highly urbanized areas; 5) persons with a below average income and 6) with stricter diagnosis criteria (diagnosis registered three times by psychiatrist). Their results remained unchanged in these subgroup analyses, showing statistically significant periodicity (by month of birth) for Alzheimer's disease, with a relatively higher risk for those born in the autumn or winter months [14].

Place of birth

Four studies performed subgroup analyses. Forster et al. looked at people with or without a family history of dementia. In both groups, the results remained non-significant.

Jean et al. performed a sex-specific analysis. There was an excess of women with AD compared to the reference population in both the urban and rural born groups. Furthermore, urban born men were less likely to develop AD compared to the reference population [15]. Jean et al. suggested it was as though the risk difference for AD between rural and urban subgroups was less important for women than for men.

Gilsanz et al. (2019) presented results separately for African American or white residents. Birth inside versus outside the highest quartile infant mortality rate states was associated with higher dementia risk for African American people and not for white people in a fully adjusted model [16]. Gilsanz et al. (2017) performed their analyses separately for black and non-black participants. For black people the hazard ratio for dementia after birth in a high-stroke mortality area was no longer significantly higher compared to those born outside high-stroke areas. For non-black participants the hazard was significantly higher when born in a high-stroke area, similar to the finding in the whole population [17]. The highest dementia risk was seen in black people born in high stroke mortality areas [17].

Glymour et al. presented their results separately for African American and white people. The increased odds for all cause dementia and AD after birth in the stroke belt compared to those born outside the stroke belt were present among both African American and white people [18]. Furthermore, Glymour et al. stratified their results by adult stroke belt residence, the results were similar across both groups and did thus not depend on adult stroke belt residence.

Other prenatal factors

Cocoros et al. evaluated their results separately for women and men, for different age groups and for inpatient dementia cases only [19]. In general, their results were similar across these subgroups, there were some statistically significant results for very specific comparisons, however, point estimates were modest. Kang et al. reported that there was no difference among men and women in their study regarding prenatal famine exposure and risk for dementia [20].

Birth characteristics

Mosing et al. only included twins and performed a within-pair analyses in monozygotic twins and in monozygotic twins and same-sex dizygotic twin pairs for z-scores of birth weight and head circumference. The within-pair analyses did not show a significant relationship between birth weight or head circumference and dementia diagnosis [21]. Sydall et al. reported sex-specific results for the relationship between birth weight and mortality from dementia or AD. In men, higher birth weight was associated with higher dementia mortality. For women, higher birth weight was associated with higher risk of AD mortality. Results in women were no longer significant when excluding women who weighted more than four kilograms at birth [22].

References

1. Bertram L, Busch R, Spiegl M, Lautenschlager NT, Muller U, Kurz A. Paternal age is a risk factor for Alzheimer disease in the absence of a major gene. Neurogenetics. 1998;1:277–80.

2. Clarnette RM, Molloy DW, McIlroy WE, Lever J, Rees L. Maternal age and Alzheimer's disease. A case control study and literature review. Dementia. 1992;3:32–7.

3. Farrer LA, Cupples LA, Connor L, Wolf PA, Growdon JH. Association of decreased paternal age and late-onset Alzheimer's disease. An example of genetic imprinting? Arch Neurol. 1991;48:599–604.

4. Hofman A, van Duijn CM, Schulte W, Tanja TA, Haaxma R, Lameris AJ, et al. Is parental age related to the risk of Alzheimer's disease? Br J Psychiatry. 2018;157:273–5.

5. Corkin S, Growdon JH, Rasmussen SL. Parental age as a risk factor in Alzheimer's disease. Ann Neurol. 1983;13:674–6.

6. English D, Cohen D. A case-control study of maternal age in Alzheimer's disease. J Am Geriatr Soc. 1985;33:167–9.

7. Farrer LA, Cupples LA, Kukull WA, Volicer L, Wells JM, Kurz A, et al. Risk of alzheimer disease is associated with parental age among apolipoprotein E epsilon4 heterozygotes. Alzheimer's Res. 1997;3:83–91.

8. Lahti M, Eriksson JG, Heinonen K, Kajantie E, Lahti J, Wahlbeck K, et al. Maternal Grand Multiparity and the Risk of Severe Mental Disorders in Adult Offspring. PLoS One. 2014;9:e114679.

9. Dysken MW, Kuskowski M, Skare SS, Roessmann U, Noronha A, Frey 2nd WH. Seasonal distribution of births in Alzheimer's disease. Int Psychogeriatrics. 1991;3:53–8.

10. Henderson AS, Korten AE, Jorm AF, McCusker E, Creasey H, Broe GA. Season of birth for Alzheimer's disease in the Southern Hemisphere. Psychol Med. 1991;21:371–4.

11. Vitiello B, Hill JL, Molchan SE, Martinez RA, Martinson HJ, Sunderl. Lack of seasonal variation in the births of patients with dementia of the Alzheimer type. Psychiatry Res. 1991;39:21–4.

12. Ptok U, Papassotiropoulos A, Maier W, Heun R. Seasonal distribution of births in patients with Alzheimer's disease and elderly depressive patients. Eur Psychiatry J Assoc Eur Psychiatr. 2001;16:157–61.

13. Ding R, He P, Song X, Zheng X. Season of birth and dementia: Findings from Chinese elderly based on a nationwide data. Am J Hum Biol. 2020;32:e23319.

14. Hsu CW, Tseng PT, Tu YK, Lin PY, Hung CF, Liang CS, et al. Month of birth and mental disorders: A population-based study and validation using global meta-analysis. Acta Psychiatr Scand. 2021;

15. Jean H, Emard JF, Thouez JP, Houde L, Robitaille Y, Mathieu J, et al. Alzheimer's disease: preliminary study of spatial distribution at birth place. Soc Sci Med. 1996;42:871–8.

16. Gilsanz P, Mayeda ER, Glymour MM, Quesenberry Jr. CP, Mungas D, DeCarli CS, et al. Birth in High Infant Mortality States and Dementia Risk in a Cohort of Elderly African American and White Health Care Members. Alzheimer Dis Assoc Disord. 2019;33:1–6.

17. Gilsanz P, Mayeda ER, Glymour MM, Quesenberry CP, Whitmer RA. Association Between Birth in a High Stroke Mortality State, Race, and Risk of Dementia. JAMA Neurol. 2017;74:1056–62.

18. Glymour MM, Kosheleva A, Wadley VG, Weiss C, Manly JJ. Geographic distribution of dementia mortality: elevated mortality rates for black and white Americans by place of birth. Alzheimer Dis Assoc Disord. 2011;25:196–202.

19. Cocoros NM, Ording AG, Horvath-Puho E, Henderson VW, Sorensen HT. In utero exposure to the 1918 pandemic influenza in Denmark and risk of dementia. Influ Other Respir Viruses. 2018;12:314–8.

20. Kang Y, Zhang Y, Feng Z, Liu M, Li Y, Yang H, et al. Nutritional Deficiency in Early Life Facilitates Aging-Associated Cognitive Decline. Curr Alzheimer Res. Bentham Science Publishers Ltd.; 2017;14:841–9.

21. Mosing MA, Lundholm C, Cnattingius S, Gatz M, Pedersen NL. Associations between birth characteristics and age-related cognitive impairment and dementia: A registry-based cohort study. PLoS Med. 2018;15:e1002609.

22. Syddall HE, Sayer AA, Simmonds SJ, Osmond C, Cox V, Dennison EM, et al. Birth weight, infant weight gain, and cause-specific mortality: the Hertfordshire Cohort Study. Am J Epidemiol. 2005/05/20. 2005;161:1074–80.
